# Text Mining and Natural Language Processing Approaches for Automatic Categorization of Lay Requests to Web-Based Expert Forums

**DOI:** 10.2196/jmir.1123

**Published:** 2009-07-22

**Authors:** Wolfgang Himmel, Ulrich Reincke, Hans Wilhelm Michelmann

**Affiliations:** ^1^Department of General Practice / Family MedicineGeorg-August-UniversityGöttingenGermany; ^2^Competence Centre Enterprise IntelligenceSAS Institute GmbHHeidelbergGermany; ^3^Department of Obstetrics and Gynaecology (Study Group of Reproductive Medicine)Georg-August-UniversityGöttingenGermany

**Keywords:** Text mining, qualitative research, natural language processing, consumer health informatics, Internet, remote consultation, infertility

## Abstract

**Background:**

Both healthy and sick people increasingly use electronic media to obtain medical information and advice. For example, Internet users may send requests to Web-based expert forums, or so-called “ask the doctor” services.

**Objective:**

To automatically classify lay requests to an Internet medical expert forum using a combination of different text-mining strategies.

**Methods:**

We first manually classified a sample of 988 requests directed to a involuntary childlessness forum on the German website “Rund ums Baby” (“Everything about Babies”) into one or more of 38 categories belonging to two dimensions (“subject matter” and “expectations”). After creating start and synonym lists, we calculated the average Cramer’s V statistic for the association of each word with each category. We also used principle component analysis and singular value decomposition as further text-mining strategies. With these measures we trained regression models and determined, on the basis of best regression models, for any request the probability of belonging to each of the 38 different categories, with a cutoff of 50%. Recall and precision of a test sample were calculated as a measure of quality for the automatic classification.

**Results:**

According to the manual classification of 988 documents, 102 (10%) documents fell into the category “in vitro fertilization (IVF),” 81 (8%) into the category “ovulation,” 79 (8%) into “cycle,” and 57 (6%) into “semen analysis.” These were the four most frequent categories in the subject matter dimension (consisting of 32 categories). The expectation dimension comprised six categories; we classified 533 documents (54%) as “general information” and 351 (36%) as a wish for “treatment recommendations.” The generation of indicator variables based on the chi-square analysis and Cramer’s V proved to be the best approach for automatic classification in about half of the categories. In combination with the two other approaches, 100% precision and 100% recall were realized in 18 (47%) out of the 38 categories in the test sample. For 35 (92%) categories, precision and recall were better than 80%. For some categories, the input variables (ie, “words”) also included variables from other categories, most often with a negative sign. For example, absence of words predictive for “menstruation” was a strong indicator for the category “pregnancy test.”

**Conclusions:**

Our approach suggests a way of automatically classifying and analyzing unstructured information in Internet expert forums. The technique can perform a preliminary categorization of new requests and help Internet medical experts to better handle the mass of information and to give professional feedback.

## Introduction

Both healthy and sick people increasingly use electronic media to obtain medical information and advice [1]. Internet users actively exchange information with others about subjects of interest or send requests to Web-based expert forums, or so-called “ask the doctor” services [2,3]. They want to understand specific diseases, to be informed about new therapies, or to ask for a second opinion before they decide on a treatment [4-6]. In addition, these expert forums also represent seismographs for medical and/or psychological needs, which are apparently not met by existing health care systems [5, 7].

In the past, emails, e-consultations, and requests for medical advice via the Internet have been manually analyzed using quantitative or qualitative methods [1-6]. To facilitate the work of medical experts and to make full use of the seismographic function of expert forums, it would be helpful to classify visitors’ requests automatically. By doing so, specific requests could be directed to the appropriate expert or even answered semiautomatically, thereby providing comprehensive monitoring. By generating “frequently asked questions (FAQs),” similar patient requests and their corresponding answers could be collated, even before the expert replies. Machine-based analyses could help both the lay public to better handle the mass of information and medical experts to give professional feedback. In addition, this method could be used to help policy makers recognize the health needs of the population [8].

Text mining [9] is a method for the automatic classification of large volumes of documents, which could be applied to the problem at hand. This technique usually consists of finite steps, such as parsing a text into separate words, finding terms and reducing them to their basics ("truncation") followed by analytical procedures such as clustering and classification to derive patterns within the structured data, and finally evaluation and interpretation of the output. Typical text-mining tasks include, besides others, text categorization, concept/entity extraction, sentiment analysis, and document summarization. This technique has been successfully applied, for example, in automatic indexing, ascertaining and classifying consumer complaints, and handling changes of address requests sent to companies by email. Text mining is also used in genome analysis, media analysis, and indexing of documents in large databases for retrieval purposes [8-11].

An automatic classification of lay requests to medical expert Internet forums is a challenge because these requests can be very long and unstructured as a result of mixing, for example, personal experiences with laboratory data. Very often, people simply require psychological help or are looking for emotional reassurance. Such heterogeneous samples of requests appear in the section “Wish for a Child” on the German Rund ums Baby (Everything about Babies) website [12], which provides information for parents, potential parents, and infertile couples.

Although involuntary childlessness is not the focus of this paper, some introductory notes on this condition may be helpful. Infertility leading to involuntary childlessness is defined as the inability of a couple to achieve conception or bring a pregnancy to term after a year or more of regular, unprotected sexual intercourse. Infertile couples may pass through different stages of reactions and feelings, which include shock, surprise, anger, helplessness, and loss of control. Feelings of failure, embarrassment, shame, and stigmatization may lead to social isolation and to a breakdown in communication between the couple, including depressive reactions, anxiety, emotional in­stability, diminished self-confidence, sexual problems, and conflicts [13].

The vast majority of cases of male infertility are due to a low sperm count, often associated with poor motility and a high rate of abnormal sperm. However, in a large number of patients (25% to 30%), it is not possible to determine the cause of the problem. The main causes of female infertility are ovarian dysfunctions and disorders of the fallopian tubes and uterus. Frequently, two or even all three causes can be found in one patient. Before 1980, infertility due to low sperm quality was treated by performing insemination with the patient’s own sperm or donor sperm. This was followed by in vitro fertilization (IVF) in the early 1980s and intracytoplasmic sperm injection (ICSI) in the early 1990s. ICSI only requires one living sperm cell [14].

Like many other conditions, involuntary childlessness is often not caused by just one factor, nor can it always be cured with a single treatment regimen. Patients and doctors alike are often confronted with the fact that they cannot find a reason for childlessness and that a treatment for a particular case is not helpful for a person or couple with a similar problem [15]. In addition to the cause itself, other factors, such as the age of the woman or problems shared by both partners, might also influence the choice of treatment. So it seems consequent that patients/couples suffering from involuntary childlessness use the Internet to get information about their infertility [6].

Requests addressed to medical expert forums such as “Wish for a Child” can be classified according to (1) the subject matter or (2) the sender’s expectation (eg, to receive a summary of the current treatment options [second opinion], to get general information about a certain disease or biological process, or to ask for advice about where to seek adequate medical help). While the first aspect is of great importance to medical experts so that they can understand the contents of requests, the latter is of interest to public health experts to allow the analysis of information needs within the population.

We carried out an initial trial to automatically classify these requests using standard text-mining software such as that provided by SAS [16,17]. However, the results of our first trial were rather disappointing since the quality of classification, expressed in terms of precision and recall, did not exceed 60% [18].

To make full use of text mining with complex data, different strategies and a combination of these strategies may refine automatic classification. The aim of this paper is to present a method for an automatic classification of requests to a medical expert forum and to evaluate its performance quality. A special focus of this method should be its flexibility to allow a precise and content-related input of expert knowledge.

## Methods

### Setting and Data

The analysis is based on a sample of requests collected from the section “Wish for a Child” on the German Rund ums Baby website [12]. In this section, visitors can participate in a medical expert forum and ask questions about involuntary childlessness. Requests and answers are openly published on the website. The structure of these dialogues resembles, for example, The Heart Forum of the Cleveland Clinic Foundation [19].

Visitors to the website ask questions directly to a group of medical experts via a Web-based interface. The expert team consists, at the moment, of eight persons who are experts in gynecology, urology, andrology, and/or embryology. Some of them work in outpatient departments, some in reproductive clinics, and some in university hospitals. So, the expert forum is well equipped to give medical advice in difficult situations, to provide help to make the correct decision, to offer a second opinion, or, in some instances, even to meet psychological needs not covered by doctors. The experts work on an honorary (unpaid) basis.

To date, more than 12,000 requests have been sent to the expert forum and have been published on the site. From these requests, we selected a random sample of 988 and classified them manually to provide a sound basis for training and evaluation.

### Manual Classification

Similar to Shuyler and Knight [20], who analyzed questions to an orthopedic website in several dimensions (topics, purpose, relationship), we decided to classify the requests into two dimensions. The first dimension (“subject matter”) comprised 32 categories (eg, assessment of pregnancy symptoms or information about artificial insemination). The second dimension (“expectations”) comprised six different categories that characterize the goals or the purpose of the sender (eg, emotional reassurance or a recommendation about treatment options).

From the very beginning of the classification process, it became apparent that many requests belong to one subject matter category but fit into more than one category of the second dimension (“expectations”). For example, a visitor asked the experts to comment on the results of a semen analysis and, at the same time, wanted some advice about whether he or she should change doctors. We decided to provide as many categories per request as appropriate. In the first dimension (“subject matter”), this request could be categorized as “semen analysis,” and, in the second dimension (“expectations”), as “discussion of results” as well as “treatment options.”

Two of the authors (HWM, WH) independently classified the first set of 100 requests manually. Because of a high rate of differing results, we defined the categories more precisely, added and removed some categories, and agreed upon the use of multiple categories. We then classified another 100 requests. This time, strong classification discrepancies, such as each author classifying the text into a different category, occurred in only 12 cases. Some minor discrepancies also occurred, such as agreement in all categories except one additional category that was suggested by one author but not the other. This resulted in a degree of agreement of 0.69 according to the kappa statistic for overlapping categories [21]. Complete agreement was achieved after further discussion and refinement of the categories and then HWM once more manually coded the first 200 and then the remaining 788 requests. The final categories of both dimensions used for classification are shown in Table 2, presented in the Results section.

**Table 1:** Terms and parents

Terms^a^	Parents
month	month
months	month
monthly	month
all months (eg, January, February)	month
all abbreviations (eg, Jan., Feb.)	month
uterus	uterus
uterus milleu	uterus
utterus	uterus
womb	uterus
in utero	uterus
uterine	uterus
adrenal gland	adrenal gland
temperature	temperature
temperture	temperature
temp.	temperature
body temperature	temperature
thermometry	temperature
all temperature degrees (eg, 37.3°C)	temperature
ultrasound	ultrasound
ultra	ultrasound
ultrasonic	ultrasound
u-sound	ultrasound
sound	ultrasound
scan	ultrasound

^a^Examples for single words, multi-word terms, synonyms, abbreviations, misspellings, etc are translated from the original German data.

### Preparation for Automatic Classification

For automatic classification, we created a dataset that contained the text from each request as a separate observation. The text was then parsed into separate words or noun groups. “Parsing” entails several techniques: (1) separation of the text into terms (eg, “uterus”) or multi-word terms (eg, “uterus milleu”), (2) normalization of different formats for dates (eg, 26/02/2008; Feb. 26, 2008) and data (eg, various degrees of temperature), (3) recognition of synonyms, and (4) stemming of verbs, nouns, or (in German) adjectives to their root form (eg, “transfer,” “transferred,” “transferring”). Programs, such as SAS Text Miner, perform this automatically and provide a complete list of all words, noun groups, and so on appearing in the text. The two authors who categorized the requests first by hand formed a detailed starting list [16] of about 10,500 relevant terms in order to include all relevant content words―even misspelled words and abbreviations. Since we focused on these words, greetings and function words such as "hello," "the," or "of" are not included and have therefore no effect upon the classification. As a next step, we clustered similar terms to create 4109 groups of terms called “parents” (for examples, see Table 1). The final dataset was a large table consisting of 998 rows (one row for each document analyzed) and 4109 columns (one column for each parent). The words in each document were analyzed to register how often each parent was represented in the text.

### Text-Mining Strategies

To reduce the final dataset consisting of 988 rows and 4109 columns, we used three techniques (as different text-mining procedures): (1) indicator variables on the basis of Cramer’s V, (2) principle component analysis (PCA), and (3) singular value decomposition (SVD). The first strategy was developed by the authors. The second strategy used the indicator variables from the first strategy as input for PCA. The third strategy made use of a standard procedure from statistic software for SVD, SAS Text Miner (SAS, Carey, NJ, USA).

#### Cramer’s V

We calculated the average Cramer’s V statistic for the association of each of the 4109 “parents” with each category and the subsequent generation of indicator variables that sum for each category all Cramer’s V coefficients over the significant words. Cramer’s V is a chi-square-based measure of association between nominal variables, with “1” indicating a complete positive association and “0” indicating no association at all. The coefficients were normalized according to the length of the texts (ie, the number of words). The selection criterion for including a parent term’s Cramer’s V was the error probability of the corresponding chi-square test. Its significance level was alternatively set at 1%, 2%, 5%, 10%, 20%, 30%, and 40%, leading to seven indicator variables per category.

#### Principle Component Analysis

We conducted PCA to reduce the seven indicator variables of varying significance levels per category into five orthogonal dimensions. PCA transforms a number of correlated variables into a few uncorrelated variables [17]. Each principal component is a linear combination of the original variables, with coefficients equal to the eigenvectors of the correlation. PCA can be used for dimensionality reduction in a dataset by retaining those characteristics of the dataset that contribute most to its variance. The data are transformed to a new coordinate system such that the greatest variance by any projection of the data comes to lie on the first coordinate (called the principal component), the second greatest variance on the second coordinate, and so on [22].

#### Singular Value Decomposition

The 500 dimensional SVD was based on the standard settings of the SAS Text Miner software [23]. To understand SVD, the whole text of all requests can be visualized as being a document by term matrix, as described above. The text from each individual request (rows) is divided into its parent terms (columns) by listing the frequency of each term in a given text. Documents are represented as vectors with length m, where m is the number of unique terms indexed in the text. The original document by term matrix is transformed, or decomposed, into smaller matrices, thus, creating a factor space. An SVD projection is a linear combination of the singular values in a row or column of the term × document frequency matrix. A high number of SVD dimensions usually summarizes the data in a better way but requires significant computing resources [24,25].

### Statistical Analyses

The sample was split into 75% training data and 25% test data. On the basis of our predictor variables (ie, 38×7 Cramer’s V indicators per category, 38×5 principle components per category, and approximately 500 SVDs), we trained logistic regression models to predict the categories. However, if all these predictor variables would be used in a regression model, it would be rather unlikely to detect any significant variables since many of these are highly correlated. Therefore, we chose a more appropriate modelling approach, a stepwise logistic regression. The choice of predictive variables was carried out by an automatic procedure.

To assess the most appropriate model for a classification, we used the following selection methods: (1) Akaike Information Criterion, (2) Schwarz Bayesian Criterion, (3) cross validation misclassification of the training data (leave one out), (4) cross validation error of the training data (leave one out), and (5) variable significance based on an individually adjusted variable significance level for the number of positive cases. For a more detailed description of most of these selection criteria, see Beal [26].

We trained for each target category, each selection criterion, and each type of input variable (Cramer’s Vs, principle components, SVDs) one logistic regression. This resulted in 1369 logistic regression models. The detailed notes and the table in the Multimedia Appendix make this procedure more transparent. For the final regression, we used meta-models, which proved the best for each of the 38 categories.

The complete training process produced an automatic method to evaluate both requests from the training sample and new requests. The corresponding software program is called score code. This score code is a function that generates, for any text (request), the probability of belonging to each of the 38 different categories.

To assess the accuracy of our approach, we calculated recall and precision as standard statistics in information retrieval and text mining for each of the 38 categories. Precision is the percentage of positive predictions that are correct (ie, a sort of specificity), whereas recall is the percentage of documents of a given category that were retrieved (sensitivity). We calculated recall and precision at the maximum F-measure [27]. To determine whether our approach yielded better results for precision and recall in the subject matter dimension or the expectation dimension, we compared the macroaverage values for precision and recall between both dimensions [28]. All statistical analyses were performed with SAS 9.1 (SAS, Carey, NJ, USA).

## Results

### Manual Classification

Table 2 shows the results of our manual classification of the 988 documents. A total of 102 (10%) documents fell into the category “in vitro fertilization (IVF)”, 81 (8%) into the category “ovulation,” 79 (8%) into “cycle,” and 57 (6%) into “semen analysis.” These were the four most frequent categories in the subject matter dimension (consisting of 32 categories). The expectation dimension comprised six categories; we classified 533 documents (54%) as “general information” and 351 (36%) as a wish for “treatment recommendations.”

**Table 2:** Quality of automatic classification

Dimension	Requests No.	Training/Validation	Validation Data	
		Ratio	Precision%^a^	Recall%^a^
**Subject Matter**				
abortion	40	30:10	91	100
abrasion	13	9:4	100	100
birth control pill	23	17:6	100	100
charges	25	18:7	100	100
clomifen	26	19:7	100	100
cryo transfer	13	9:4	100	75
cycle	79	59:20	80	86
cysts	16	12:4	100	100
endometriosis	11	8:3	75	100
examination of the oviduct	19	14:5	100	100
habitual abortion	17	12:5	100	100
hormones	36	27:9	78	78
insemination	29	21:8	100	100
intermenstrual bleeding	14	10:4	100	100
IVF	102	76:26	81	88
luteal phase defects	25	18:7	88	100
medical drugs	47	35:12	92	100
menstruation	35	26:9	90	100
multiples	7	5:2	100	100
naturopathy	33	24:9	90	100
nourishment	9	6:3	100	100
oviduct	16	12:4	100	100
ovulation	81	60:21	90	86
PCO	27	20:7	100	100
pregnancy symptoms	36	27:9	100	100
pregnancy test	30	22:8	88	88
pregnancy worries	49	36:13	100	92
semen analysis	57	42:15	88	93
sexual intercourse	14	10:4	100	100
sexual intercourse, problems	5	3:2	100	100
stimulation	40	30:10	63	100
thyroid glands	13	9:4	100	100
**Expectations^b^**				
current treatment	331	248:83	85	72
discussion of results	310	232:78	86	82
emotions	90	67:23	100	61
general information	533	399:134	92	84
interpretation of own situation	242	181:61	78	69
treatment options	351	263:88	82	81

^a^To calculate recall and precision, we first chose the best model according to the following selection criteria: Akaike’s Information Criterion, Schwarz Baysian Criterion, cross validation misclassification of the training data, cross validation error of the training data; then we determined the optimum compromise between recall and precision by the F-measure.

^b^Multiple categories possible.

### Automatic Classification

We used different selection criteria to find the best regression models for training and validation. In about half of the categories, the generation of indicator variables based on the chi-square analysis proved to be the best approach for automatic classification. Other categories were best predicted by using either PCA or SVD. Statistical details are shown in the Multimedia Appendix. A 100% precision and 100% recall was realized in 18 out of 38 categories on the validation sample (see Table 2). The lowest rates for precision and recall were 75% and 61%, respectively. The rates for precision and recall were, on average, somewhat lower in the expectations dimension (78.2% and 74.8%, respectively) compared to the subject matter dimension (93.6% and 96.4%, respectively).

Table 3 and Table 4 provide exemplary impressions of the power and the limits of the chi-square analysis. Table 3 lists the most significant words in the category “general information.” Interestingly, nearly all of the first 50 words for the category “general information” were negatively associated. This means that the word “injection,” for example, is a strong indicator that a document containing this word does not belong to this code. The 51st word (“fertile”) was the first one with a positive Cramer’s V; it represents a typical question about the fertile days of the menstrual cycle. For nearly all other categories, the most predictive words were positively associated with the respective category.

Table 4 lists the most significant words for the categories “oviduct” and “examination of the oviduct.” These categories have been listed separately because “oviduct” was mainly associated with lay requests about reproductive medicine in general while “examination of the oviduct” was used in conjunction with questions about specific treatments or treatment options. Some of the predictive words (eg, “tube,” “fallopian tube,” “level”) are the same in both categories. For example, the word “tube” appears in all requests that we categorized by hand as “oviduct” (n = 16), showing a strong predictive value (Cramer’s V of 0.44). However, this word also appears in 79% of the requests that were categorized as “examination of the oviduct” (n = 19). Again, Cramer’s V was high (0.37), signalling also the strong predictive value of this word for “examination of the oviduct”. In this situation, only the summary of the Cramer’s V statistic as an indicator variable guaranteed high precision and recall, and not a single word alone.

For some categories, the input variables also included variables from other categories, most often with a negative sign. For example, the meta-model for “pregnancy test” included a sample of words (as an indicator variable) predictive for the category “menstruation” with a negative sign. This means that absence of words predictive for “menstruation” was a strong indicator for the category “pregnancy test”.

For other categories, consideration of a sender’s expectation also contributed to a better classification of requests. For example, the meta-model for “hormones” included a sum of relevant terms (on the basis of Cramer’s V) as well as significant terms demonstrating the expectation to learn more about one’s own situation or to have laboratory data interpreted (both with negative signs, meaning that the absence of these expectations were, besides others, indicators for “hormones”).

**Table 3:** Most predictive words for the category “general information”

Word	Frequency, No. (%)		Cramer’s V	*P*
	In “General Information”	In Other Categories		
X-chromosome	70 (13)	143 (31)	− 0.22	< .001
injection	17 (3)	68 (15)	− 0.21	< .001
utrogest	7 (1)	45 (10)	− 0.19	< .001
clomifen	32 (6)	82 (18)	− 0.19	< .001
prescribe	10 (2)	45 (10)	− 0.17	< .001
write	21 (4)	59 (13)	− 0.16	< .001
med	45 (8)	88 (19)	− 0.16	< .001
drug	24 (5)	59 (13)	− 0.15	< .001
pill	20 (4)	53 (12)	− 0.15	< .001
value	48 (9)	88 (19)	− 0.14	< .001
[places 11-50]				
fertile	36 (7)	44 (10)	0.12	< .001

**Table 4:** Most predictive words for the categories “oviduct” (total requests = 16) and “examination of the oviduct” (total requests = 19)

Category	Requests in Which This Word Occurs^a^	Cramer’s V	*P*
Word	No. (%)		
**“Oviduct” (n = 16)**			
tube	16 (100)	0.44	< .001
fallopian tube	16 (100)	0.44	< .001
removed	8 (50)	0.40	< .001
exception	2 (13)	0.35	< .001
away	8 (50)	0.29	< .001
link	7 (44)	0.28	< .001
move	1 (6)	0.25	< .001
obliterate	1 (6)	0.25	< .001
inappropriate	1 (6)	0.25	< .001
sterilisation	1 (6)	0.25	< .001
secretion	1 (6)	0.25	< .001
scar	1 (6)	0.25	< .001
opportunity	1 (6)	0.25	< .001
patent	1 (6)	0.25	< .001
open	1 (6)	0.25	< .001
consider	1 (6)	0.25	< .001
extensive	1 (6)	0.25	< .001
attachment	1 (6)	0.25	< .001
abandon	1 (6)	0.25	< .001
cut	1 (6)	0.25	< .001
tubal pregnancy	4 (25)	0.24	< .001
endoscopy	7 (44)	0.21	< .001
level	8 (50)	0.21	< .001
**“Examination of the Oviduct” (n = 19)**			
tube	15 (79)	0.37	< .001
fallopian tube	15 (79)	0.37	< .001
laparoscopy	11 (58)	0.35	< .001
endoscopy	12 (63)	0.35	< .001
X-ray	3 (16)	0.34	< .001
angiography	2 (11)	0.32	< .001
examination	4 (21)	0.32	< .001
level	12 (63)	0.30	< .001
penetrable	4 (21)	0.27	< .001
stomach	11 (58)	0.27	< .001
hsg	2 (11)	0.26	< .001
structure	12 (63)	0.23	< .001
cycle	1 (5)	0.23	< .001
adhere	1 (5)	0.23	< .001

^a^Some words in this table occur only once or twice (eg, “move”), but not at all in any of the other subject categories. Therefore, they still have predictive power (with a significant Cramer’s V).

### Exemplary Comparison Between Automatic and Manual Classification

To give a more vivid picture of the results of our method, we present some of the visitors’ requests, including our own manual classification and the automatic classification with scoring values for the probability of falling into a particular category (see Table 5). The first example is a very short request in which the sender wants to know whether a short cycle could be caused by a particular hormone. The automatic classification did not find the central topic of the request, probably because the term “prolactinspiegel” (prolactin level) was not recognized as “hormones.” The subject category with the highest probability was “cycle,” with a probability of only 2%―meaning that no classification was automatically assigned. In the two other examples, all our manual codes were recognized by the automatic classification. This was also the case in most other requests, representing a high sensitivity of our approach.

In several instances, and also in two of the three examples presented in Table 5, the automatic classification sometimes gave a high score not only for the correct subject category (as determined by the authors) but also for additional subject categories. In the second example, there was a high score for “stimulation” (in addition to the correct “IVF”), and the categories “clomifen” and “stimulation” scored highly in the third example (together with the correct category “multiples”). Consequently, precision, which is a measure of specificity, was not always entirely satisfactory. Some of these additional classifications such as “stimulation” in the second and third examples are provoked by the word “stimulation” or other misleading words in the request. While the additional categories in the automatic classification are not entirely correct, they are also not completely wrong. In all three of these examples, our classification according to the expectation of the sender was confirmed by the automatic classification with different probabilities. Only in the last example did the automatic classification also select “treatment options,” which in fact is not entirely incorrect.

**Table 5:** Sample visitor requests and their classification

*After a very long first half of my cycle (14-20 days), the second half of the cycle only takes 8 days. Is this probably because of an elevated prolactin level (I still nurse)? Many thanks for your answer, [Name]* Expert classification: hormones; general information; current treatment Automated classification: cycle (2%); general information (99%), current treatment (97%)
*We are in the middle of our second IVF cycle. During our first follicular puncture (first IVF), only one fallopian tube could be punctured [sic]. The second was hidden behind the uterus. However, at that time, the stimulation regime[n] was quite high. In the current cycle, I was stimulated with consideration. Therefore, only 11 follicles grew. At first, my question regarding sports activities during stimulation was answered with “no problems.” After another inquiry I was told that I should stop playing badminton after the 8th day of stimulation. However, swimming would not be a problem. Because of badminton, torsion of the fallopian tube may happen. Today, follicular puncture took place. For the last time, I played badminton on day 8 of stimulation (only half out) but went swimming up to day 11 of stimulation (but not as “hard” as usual) because, supposedly, this should not have any effect.* Expert classification: IVF; general information; current treatment Automatic classification: IVF (99%); stimulation (68%); general information (99%); current treatment (97%)
*Right now, I am in the middle of my second insemination cycle (stimulation with Puregon 50 and Clomifen). Today, on day 12 of the cycle, 4 big follicles are visible. Now I shall decide whether to stop the cycle or to get inseminated. What are your thoughts about the risk of multiples? I would accept twins but not triplets. I am torn…. On one hand I would like to take the chance to get pregnant, but on the other hand I am afraid of multiples. Please tell me your opinion. Because of your experience you might be able to judge this matter much better. I appreciate your answer. Sincerely yours, [Name]* Expert classification: multiples; general information; current treatment Automatic classification: multiples (98%); clomifen (68%); stimulation (54%); general information (67%); current treatment (98%); treatment options (53%)

## Discussion

A combination of different text-mining strategies should classify requests to a medical expert forum into one or several of 38 categories, representing either the subject matter or the sender’s expectations. This combined strategy yielded rates of precision and recall above 80% in nearly all categories. Even in the worst classified categories, the rates were at least above 60%.

### Meaning of the Study

In order to evaluate these results, the exceptional character of this text-mining process should be considered. The documents to be classified were complex, sometimes rather long, and, most importantly, needed to be classified not only according to content but also to their (sometimes subliminal) expectations. We were able to show that a combination of different text-mining procedures was superior to a single method. Two factors have particularly contributed to this success: (1) an elaborated starting list and (2) a combination of chi-square statistics, PCA, and an SVD method. These factors mirror a recommendation and an experience reported by Balbi and Meglio [29], who built their specific text-mining strategy according to the “nature” of the data.

The creation of good starting (or stopping) lists is necessary to obtain valid and useful results, and comprehensive domain knowledge is essential for creating reasonable lists in the first place. The lists described here contain valuable expert knowledge in the field of involuntary childlessness. It seems reasonable to suppose that creating synonym lists in other medical areas could also be a powerful tool for successful text mining in other Internet forums. In their extensive paper on predictive data mining, Bellazzi and Zupan [30] stress the importance of additional knowledge that domain experts can make use of for the modeling methods. This starting list demonstrated its full potential when used to generate indicator variables that summed all Cramer’s V values for each request and each category over the significant words. This way, we escaped the danger of overestimating the predictive power of single words, especially if words are negated (eg, “I’m not interested in IVF” or “my cycle is not normal”).

Nearly all words predictive of the category “general information” were negatively associated in the chi-square statistic. This seems to be a “perfect” finding and evidence for our content-related approach since any treatment with injections, for example, would belong to the categories “treatment options,” “interpretation,” or “current treatment” rather than the category “general information.” It is precisely the lack of technical terms or results from prior investigations that defined this category.

Experts usually classify requests, such as the ones we analyzed, in a dichotomous way (ie, either they do or do not belong to a respective category). In contrast, automatic classification with a scoring system similar to the one presented in this paper gives a probability for any given request to fall into any of the categories. Especially in the case of complex texts, it seems appropriate to classify them into multiple dimensions and multiple categories. We defined a cutoff of 50% for our scoring system (ie, we defined a request to fall into a category if the respective score was over 50%). At the same time, it is possible to change the cutoff according to the purpose of an analysis. For example, if we are interested in recognizing possible health needs, a 50% cutoff may contribute to a high recall (sensitivity) so that we do not miss relevant requests. If we are interested in high precision (ie, specificity of classification) to sort out the requests and thus to support the experts’ work, a higher cutoff may be reasonable. Our analysis procedure permits an easy assignment of different cutoff values.

There is another reason why this scoring procedure seems adequate or even superior to a dichotomous expert classification. When we analyze the sender’s expectation, we are usually confronted with a mix of different expectations. In many cases, we classified a request into several expectation dimensions. This seems intuitively better represented by a scoring procedure such as the one presented in this paper. And even the subject matter classifications that we employed in our manual procedure as separated (disjunct) categories may not be as clear as they seem in many requests. It is rather likely that a given request may also fall into more than one subject matter, as demonstrated by the examples in Table 5, so that in these cases, a scoring procedure that also permits overlapping categories seems most appropriate [6,20]. In contrast, most studies, even if they have used a multidimensional categorizing scheme such as Shuyler and Knight [20], only permit one category per dimension.

As SVD is a powerful method for automatic classification, it seemed quite logical that this approach proved best to predict categories in about a quarter of instances. However, there is sometimes reluctance to use SVD-based classification strategies because this process can be controlled only to a limited degree [31]. In other words, text mining based on SVD is a procedure that cannot be consciously monitored. As a sort of black box, it automatically runs in the background and we have to rely on the validity of this procedure. In contrast, according to Reincke [31], the data-mining process should be mapped into a continuous IT flow that controls the entire information from the raw data, cleaning aggregation and transformation, analytical modeling, operative scoring, and last but not least, final deployment. In this sense, our analysis is actually far more transparent as demonstrated in the case of the predictive words given in Table 3. That is to say, our analysis not only yields good rates for precision and recall, but it also provides us with a complete view of the analytic process and thus contributes to face validity.

In the last decade, the medical profession has witnessed new developments whereby patients have become their own experts, often through the adoption of strategies to empower themselves [32] and often supported by the Internet [33,34] with email consultation services for electronic patient–caregiver communication [35]. A crucial factor to be able to make use of all of this potential information is time. The Internet is a rapid medium and when questions go unanswered for a few days, users are disappointed and may even resend their queries, as Marco et al [3] experienced in their Internet survey on AIDS and hepatitis. A complex technological solution such as that presented in this paper may effectively help medical experts to process the information needs of requests in advance and to accelerate response times. Once the information needs have been understood, it will also be possible to find similar previous requests, allowing experts to make efficient use of their earlier answers. This technology can therefore be used to both enable experts to answer requests promptly and to lighten their workload.

As a further advantage of our approach, we would like to emphasize our comprehensive list of categories. To date, analyses of email requests [5,6] have tried to categorize these requests in more or less simple categories, especially to learn more about information needs and the possible workload of experts. In contrast, we have been far more specific in the classification of the information needs with 32 categories representing the subject matter dimension. This detailed classification is exactly what experts need if machine-based analysis is to support their work.

### Limitations of the Study

The classification of requests according to the senders’ expectations could be improved. That this process is not optimal may be due to the somewhat vague definition of what exactly constitutes a certain patient’s expectation, and this requires improvement if health experts are to make conclusions about the health needs of a population. However, the overall performance of the subject classification seems to be sufficient, so much so that semiautomated answers to senders’ requests, in this medical area, may be a realistic option for the future.

### Future Considerations

We consider there to be three relevant applications of our text-mining procedures in the near future:

1. If our scoring procedure proves successful in further tests, it could be integrated into the Rund ums Baby website to facilitate semiautomated answer proposals to be used by the experts and, in cases when classification accuracy is high, direct automated answers to the patients [36]. A multidimensional classification of texts, as in our approach, may be especially appropriate for this purpose since we recognize not only the plain content (ie, subject matter) but also the sender’s expectations, something like a hidden subtext.
2. A retrospective application of the scoring procedure to all accumulated requests would allow their mapping into different categories, thus providing an objective historical seismograph and allowing a better understanding of medical and psychological needs that have yet to be met by the current health care system.
3. The scored database forms the basis for a sophisticated FAQ Internet page that does not address those questions and issues considered by experts to be the most important, as is usually the case, but one which is more oriented to the real needs of visitors and patients.

We are not aware of any studies that have tried to analyze similarly complex texts in Internet forums. Further studies are therefore needed to compare and refine our methodology. Then it should also be possible to decide which aspects of our text-mining strategies—the expert-based synonym list or the combination of different strategies—were most important for the success of our automatic classification.

### Conclusions

Our analysis suggests a way of classifying and analyzing complex documents to provide a significant as well as a valid information source for politicians, administrators, researchers, and/or counselors. In the case of involuntary childlessness, it will be possible to fulfill not only patients’ information and health needs with this Internet expert forum, but also to analyze and follow-up these needs over long periods of time. These techniques also seem promising for the analysis of large samples of documents from other Internet health forums, chat rooms, or email requests to doctors.

## Multimedia Appendix 1

Statistical details of the automatic classification (explanation)

## Multimedia Appendix 2

Statistical details of the automatic classification (table)

## Abbreviations

FAQ: frequently asked question
ICSI: intracytoplasmic sperm injection
IVF: in vitro fertilization
PCA: principle component analysis
SVD: singular value decomposition
