# Supplementary material for: Text Mining and Natural Language Processing Approaches for Automatic Categorization of Lay Requests to Web-Based Expert Forums
Source: J Med Internet Res. 2009 Jul 22;11(3):e25. doi: 10.2196/jmir.1123 (PMC2762848; doi:10.2196/jmir.1123)
Supplement: Supplementary file 1 [file jmir_v11i3e25_app1.pdf]

Multimedia Appendix 1 (

Statistical Details of the Automatic Classification

Explanation of the Appendix Table:

Multimedia Appendix 2 shows details of our automatic classification of a sample of requests in the section "Wish for a child" on www.rund-ums-baby.de. The following table includes 8 columns and 114 (38*3) rows. Each row corresponds to one of the 3 top classification models for a specific category with 3 different selection criteria used for the stepwise regression model. In the following, the 8 columns are shortly described to help the reader understand the large table at the end of the Appendix and to get a vivid impression of our approach.

Example from columns 1-3

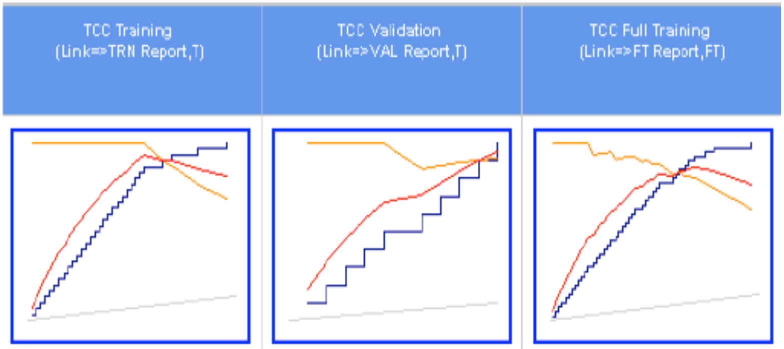

Columns 1-3 show the exact target concentration curve (see http://en.wikipedia.org/wiki/Receiver_operating_characteristic) for training (TRN), validation (VAL) and “full training” (FT) data. “Full training” refers to the union of our training and validation samples. These charts provide an immediate overview, on how good the classification works for each model in each category. The yellow line is the precision, the blue line the recall and the red line the F-statistic. Precision and recall are determined at the maximum F-measure.

Example from column 4

Category<br>Number of Doc<br>(TRN,VAL,FT)<br>Restriction
1.Abot<br>(30,10,40)<br>k_XMISC_30<br>Ginni_TRN:95<br>Ginni_VAL:95<br>Ginni__FT:95

Column 4 provides the name of the classification category (in this case “1.Abort” [=abortion]), followed by 3 number in brackets, indicating the number of positive documents in the training, validation and “full training” samples (30, 10, 40; see also Table 2 in the paper). In each third line the type of input variables and variable selection criterion used for the stepwise regression model are given: the first string can be “k”, “pc”, or “svd”, dependent on which type of input variable was used, with “k” = the chi square based k-indicator variable, “pc” = “principal component” and “svd” = singular value dimensions. The second string specifies the variable selection criterion in the stepwise regression model:

- _XMISC means crossvalidation (misclassification)
- _AIC means Akaike Information Criterion (AIC)
- _SBC means Schwarz Bayesian Criterion (SBC)
- XERROR means crossvalidation (error).

The third string in the third line lists the significance level used in the chi squared test for determining the significant words that contribute to a k-indicator variable (in this case, a 30 percent level). Finally line 5-7 show the Gini coefficient in percent (see http://en.wikipedia.org/wiki/Gini_coefficient) of the training, validation and full training data.

Example from column 5

Regression Model Training (Estimate,Chisq)
Intercept(-6.11,-3.66)
k_30_1(31.69,2.90)
k_30_11(-5.81,-3.02)
k_20_1(-20.82,-2.34)
k_10_2(-4.14,-2.11)
k_5_37(-3.42,-2.93)

Column 5 lists the full regression model on the training data, with the selected variables. The column begins with the intercept. The selected variables (in this case, 5 variables) are characterized by their estimated regression coefficient and the chi square statistic of significance in the regression model (all information in brackets). The variable names are made up of 2 or 3 strings: First the type of variable “k”, “pc”, or “s” as explained for column 4 then - in case of a “k”-type variable - if applicable the significance level used in the chi squared test for determining the significant words that contribute to a k-indicator variable, and finally the category for which the chi squared test was made. The example (k_30_1) shows a strong positive and significant effect of the k-indicator variables for abortion (=1) at the 30% significance level with a regression coefficient of 31.69, which is partially offset by all other variables in the model (negative and significant coefficients).

Example from column 6

Regression Model Full Traininig (Estimate,Chisq)
Intercept(-4.43,-4.90)
k_30_1(4.62,6.77)
k_10_18(-1.98,-1.41)
k_5_37(-1.55,-3.51)

Column 6 lists the full regression model on the “full training” data. The details are the same as outlined in column 5 for the training data.

Example from Column 7

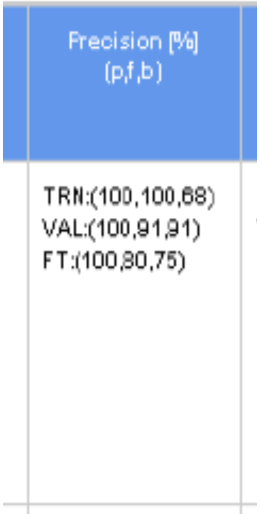

Column 7 shows the precision (according to the yellow line in column 1-3) on training (TRN), validation (VAL) and “full training” (FT) data in the three lines. The 3 numbers in brackets are the precision at different vertical cutoff values. The first number is the precision at a cutoff value where precision is at its maximum and recall (blue line in column 1-3) is “as high as possible” (without losing precision). The second number is the precision at a cutoff value, where the F Statistic (red line in column 1-3) is at its maximum. The third number is the precision at a cutoff value where recall is at its maximum and precision is “as high as possible” (without losing recall).

Example for column 8

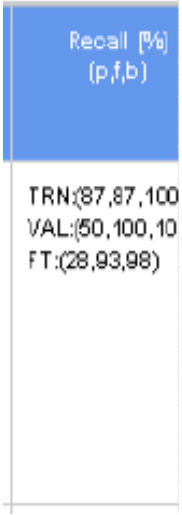

Column 8 is the same for recall as column 7 for precision. The legend would be the same as for column 7; it is necessary to replace “precision” with “recall” and vice versa.
