# Supplementary material for: Text Mining and Natural Language Processing Approaches for Automatic Categorization of Lay Requests to Web-Based Expert Forums
Source: J Med Internet Res. 2009 Jul 22;11(3):e25. doi: 10.2196/jmir.1123 (PMC2762848; doi:10.2196/jmir.1123)
Supplement: Supplementary file 2 [file jmir_v11i3e25_app2.pdf]

TCC Training (Link=>TRN Report,T)	TCC Validation (Link=>VAL Report,T)	TCC Full Training (Link=>FT Report,FT)	Category Number of Doc (TRN,VAL,FT) Restriction	Regression Model Training (Estimate,Chisq)	Regression Model Full Traininig (Estimate,Chisq)	Precision [%] (p,f,b)	Recall [%] (p,f,b)
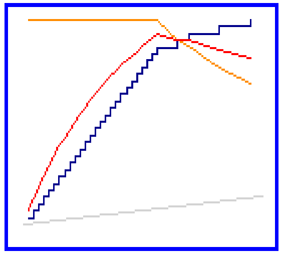	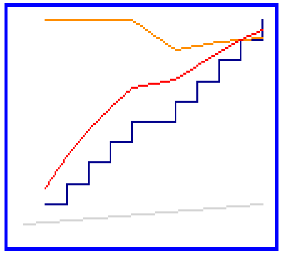	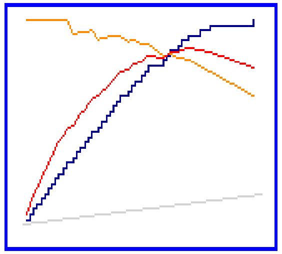	1. Abort<br>(30,10,40)<br>k_XMISC_30<br>Ginni_TRN: 95<br>Ginni_VAL: 95<br>Ginni__FT: 95	Intercept(-6.11,-3.66)<br>k_30_1(31.69,2.90)<br>k_30_11(-5.81,-3.02)<br>k_20_1(-20.82,-2.34)<br>k_10_2(-4.14,-2.11)<br>k_5_37(-3.42,-2.93)	Intercept(-4.43,-4.90)<br>k_30_1(4.62,6.77)<br>k_10_18(-1.98,-1.41)<br>k_5_37(-1.55,-3.51)	TRN:(100,100,68)<br>VAL:(100,91,91)<br>FT:(100,80,75)	TRN:(87,87,100<br>VAL:(50,100,10<br>FT:(28,93,98)
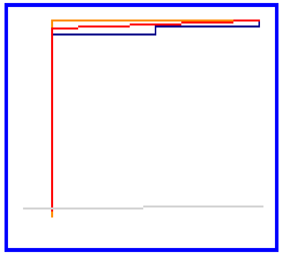	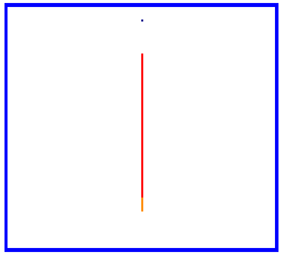	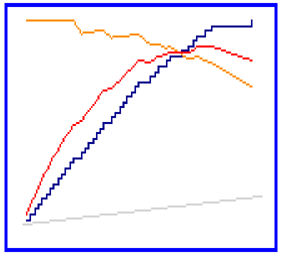	1. Abort<br>(30,10,40)<br>k_AIC_40<br>Ginni_TRN: 91<br>Ginni_VAL: 87<br>Ginni__FT: 95	Intercept(-592.99,-4.10)<br>k_40_1(5148.85,.)<br>k_30_11(-1107.1,.)<br>k_30_30(-1088.7,.)<br>k_20_1(-3593.0,.)<br>k_5_37(-325.29,-4.84)	Intercept(-4.43,-4.68)<br>k_40_1(4.69,6.64)<br>k_10_18(-2.14,-1.39)<br>k_5_37(-1.62,-3.52)	TRN:(100,100,100)<br>VAL:(71,71,71)<br>FT:(100,80,68)	TRN:(100,100,1<br>VAL:(100,100,1<br>FT:(28,98,100)
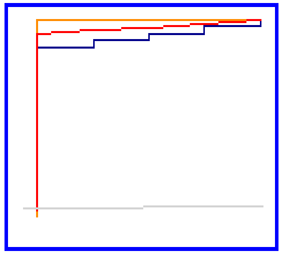	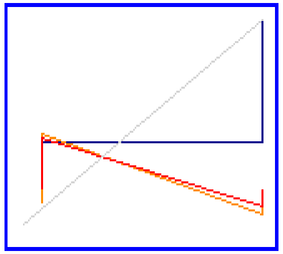	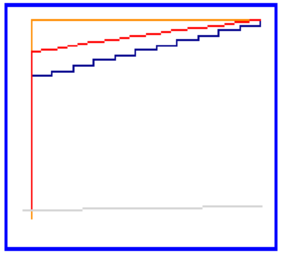	1. Abort<br>(30,10,40)<br>svd_AIC_40<br>Ginni_TRN: 92<br>Ginni_VAL: 37<br>Ginni__FT: 93	Intercept(-6125.2,-144.42)<br>s2(6544.01,3.99)<br>s3(-10521,-45.62)<br>s5(17455.5,.)<br>s9(26591.9,.)<br>s12(-19518,.)<br>s43(7684.73,.)<br>s46(-19990,.)<br>s59(11938.4,5.41)<br>s64(17114.7,.)<br>s167(-20415,-14.72)<br>s223(-3740.1,-4.94)<br>s289(2977.28,2.27)<br>s346(-5495.6,-4.43)<br>s360(6795.72,.)<br>s427(-7873.4,.)	Intercept(-1370.7,-0.78)<br>s1(1860.72,.)<br>s3(-1981.7,-0.46)<br>s5(1198.18,.)<br>s9(2461.75,.)<br>s15(1707.17,0.12)<br>s17(-3249.7,-0.15)<br>s26(2002.16,0.18)<br>s36(-1461.6,.)<br>s46(-2618.3,.)<br>s48(-1978.9,-0.06)<br>s55(2327.65,0.08)<br>s58(-1331.9,-0.06)<br>s124(-1062.6,.)<br>s147(2327.80,.)<br>s172(-2346.1,-0.07)<br>s198(1569.90,0.08)<br>s202(2194.99,0.11)<br>s304(-609.70,-0.07)<br>s346(-1101.2,-0.23)<br>s373(749.00,0.04)<br>s493(780.56,0.06)	TRN:(100,100,100)<br>VAL:(44,44,44)<br>FT:(100,100,100)	TRN:(87,100,10<br>VAL:(40,40,40)<br>FT:(78,100,100)

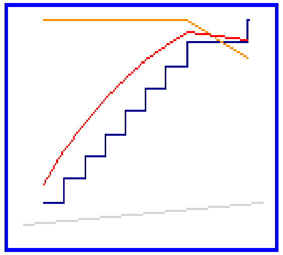	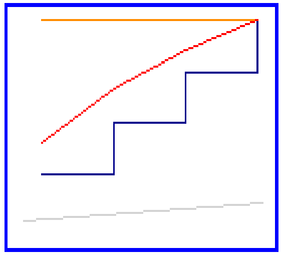	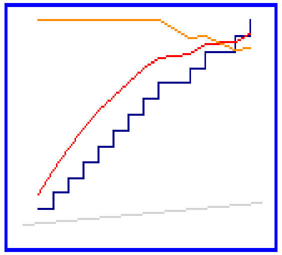	2. Ausschabung<br>(9,4,13)<br>k_XERROR_1<br>Ginni_TRN: 95<br>Ginni_VAL: 93<br>Ginni__FT: 95	Intercept(-6.79,-3.26)<br>k_1_2(7.25,2.92)	Intercept(-6.98,-3.87)<br>k_1_2(9.44,3.46)	TRN:(100,100,82)<br>VAL:(100,100,100)<br>FT:(100,87,87)	TRN:(89,89,100<br>VAL:(100,100,1<br>FT:(69,100,100
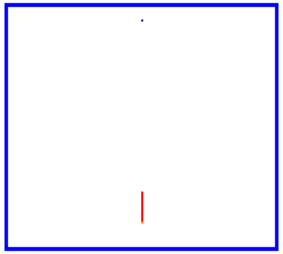	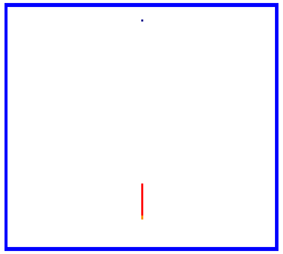	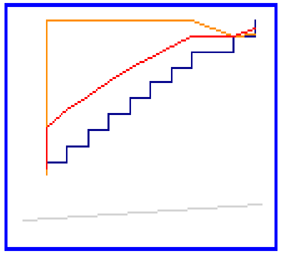	2. Ausschabung<br>(9,4,13)<br>k_XERROR_20<br>Ginni_TRN: 0<br>Ginni_VAL: 0<br>Ginni__FT: 95	Intercept(-2.38,-6.82)	Intercept(-26.02,-1.56)<br>k_20_2(22.48,1.57)	TRN:(8,8,8)<br>VAL:(11,11,11)<br>FT:(100,93,93)	TRN:(100,100,1<br>VAL:(100,100,1<br>FT:(85,100,100
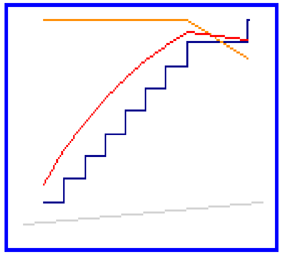	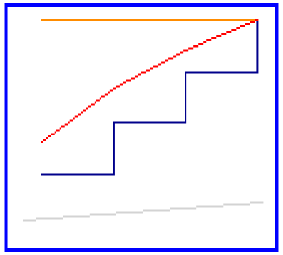	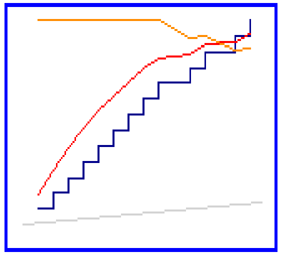	2. Ausschabung<br>(9,4,13)<br>k_XERROR_1<br>Ginni_TRN: 95<br>Ginni_VAL: 93<br>Ginni__FT: 95	Intercept(-6.79,-3.26)<br>k_1_2(7.25,2.92)	Intercept(-6.98,-3.87)<br>k_1_2(9.44,3.46)	TRN:(100,100,82)<br>VAL:(100,100,100)<br>FT:(100,87,87)	TRN:(89,89,100<br>VAL:(100,100,1<br>FT:(69,100,100

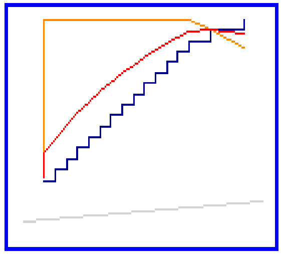	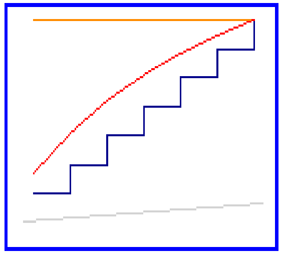	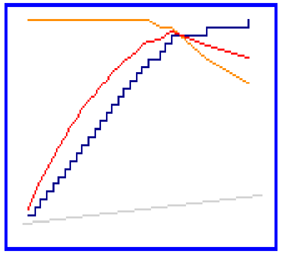	3. Clomifen<br>(19,7,26)<br>k_AIC_10<br>Ginni_TRN: 95<br>Ginni_VAL: 94<br>Ginni__FT: 95	Intercept(-15.63,-1.69)<br>k_5_30(-10.70,-1.53)<br>k_1_3(40.83,1.60)<br>k_1_12(-19.97,-1.42)	Intercept(-8.82,-3.70)<br>k_2_3(14.73,2.95)<br>k_1_36(-7.64,-1.37)	TRN:(100,95,86)<br>VAL:(100,100,100)<br>FT:(100,96,68)	TRN:(79,95,100<br>VAL:(100,100,1<br>FT:(81,92,100)
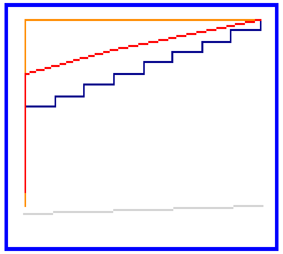	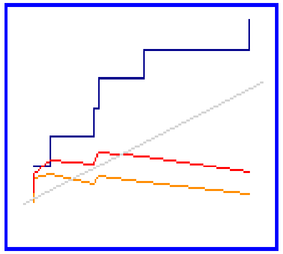	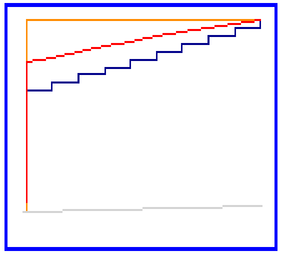	3. Clomifen<br>(19,7,26)<br>svd_XMISC_40<br>Ginni_TRN: 94<br>Ginni_VAL: 71<br>Ginni__FT: 93	Intercept(-386.62,-0.60)<br>s10(-1041.5,-0.24)<br>s18(1262.04,.)<br>s21(688.00,.)<br>s69(981.08,0.19)<br>s89(1794.53,0.71)<br>s103(-1039.4,-0.12)<br>s136(-1641.3,-0.21)<br>s259(-621.81,-0.54)<br>s321(696.41,0.44)<br>s369(354.24,0.15)<br>s482(-776.11,-0.29)	Intercept(-550.17,-1.53)<br>s10(-1325.0,-0.47)<br>s17(1011.35,.)<br>s21(2374.40,.)<br>s23(-1202.6,-0.56)<br>s24(1129.70,.)<br>s76(1977.89,1.36)<br>s83(1820.77,0.14)<br>s121(1536.97,.)<br>s166(-1880.1,-0.90)<br>s174(-1051.2,-0.46)<br>s223(2085.20,0.44)<br>s275(466.00,.)<br>s321(431.89,0.19)<br>s482(-595.69,-0.14)<br>s494(-944.98,-0.17)	TRN:(100,100,100)<br>VAL:(25,24,21)<br>FT:(100,100,100)	TRN:(79,100,10<br>VAL:(43,71,86)<br>FT:(100,100,10
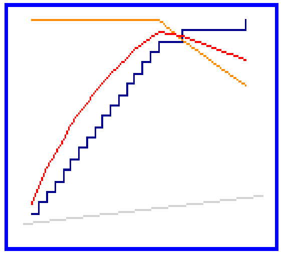	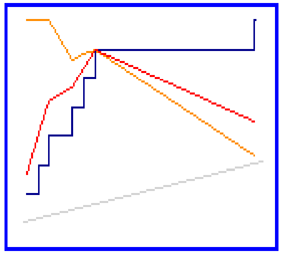	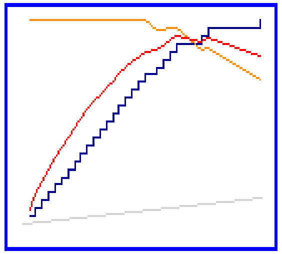	3. Clomifen<br>(19,7,26)<br>k_XERROR_1<br>Ginni_TRN: 95<br>Ginni_VAL: 91<br>Ginni__FT: 95	Intercept(-16.55,-2.85)<br>k_1_3(16.91,2.92)<br>k_1_8(-29.72,-2.25)	Intercept(-9.39,-3.51)<br>k_1_3(16.01,3.16)<br>k_1_36(-6.30,-1.44)	TRN:(100,100,68)<br>VAL:(100,86,86)<br>FT:(100,96,70)	TRN:(79,89,100<br>VAL:(43,86,86)<br>FT:(73,88,100)

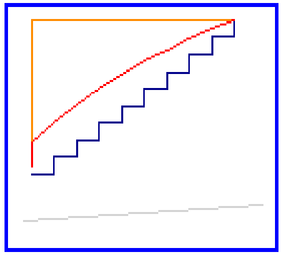	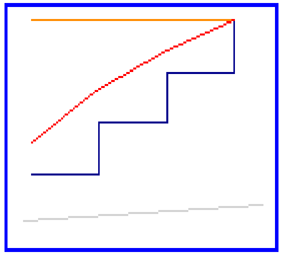	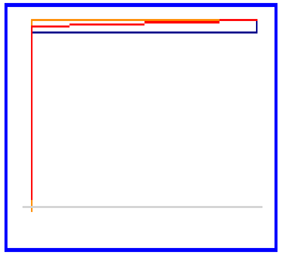	4. Eileiter<br>(12,4,16)<br>k_SBC_30<br>Ginni_TRN: 95<br>Ginni_VAL: 94<br>Ginni__FT: 91	Intercept(-15.29,-0.61)<br>k_30_4(17.55,0.22)<br>k_30_37(-11.39,-0.17)	Intercept(-111.76,-9.28)<br>k_30_4(227.92,.)<br>k_30_37(-172.96,.)	TRN:(100,100,100)<br>VAL:(100,100,100)<br>FT:(100,100,100)	TRN:(100,100,1<br>VAL:(100,100,1<br>FT:(100,100,10
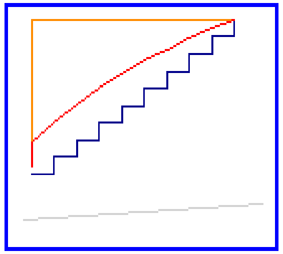	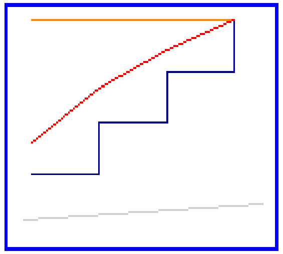	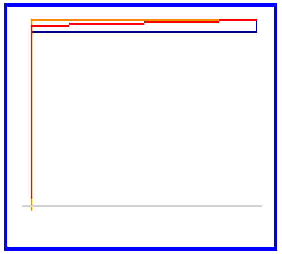	4. Eileiter<br>(12,4,16)<br>k_SBC_30<br>Ginni_TRN: 95<br>Ginni_VAL: 94<br>Ginni__FT: 91	Intercept(-15.29,-0.61)<br>k_30_4(17.55,0.22)<br>k_30_37(-11.39,-0.17)	Intercept(-111.76,-9.28)<br>k_30_4(227.92,.)<br>k_30_37(-172.96,.)	TRN:(100,100,100)<br>VAL:(100,100,100)<br>FT:(100,100,100)	TRN:(100,100,1<br>VAL:(100,100,1<br>FT:(100,100,10
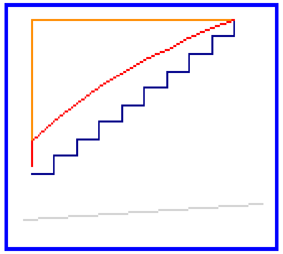	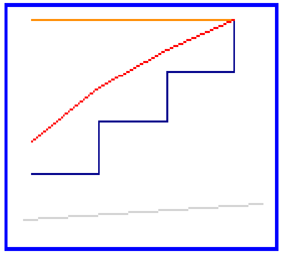	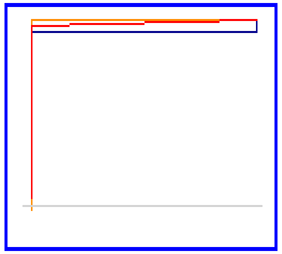	4. Eileiter<br>(12,4,16)<br>k_SBC_30<br>Ginni_TRN: 95<br>Ginni_VAL: 94<br>Ginni__FT: 91	Intercept(-15.29,-0.61)<br>k_30_4(17.55,0.22)<br>k_30_37(-11.39,-0.17)	Intercept(-111.76,-9.28)<br>k_30_4(227.92,.)<br>k_30_37(-172.96,.)	TRN:(100,100,100)<br>VAL:(100,100,100)<br>FT:(100,100,100)	TRN:(100,100,1<br>VAL:(100,100,1<br>FT:(100,100,10

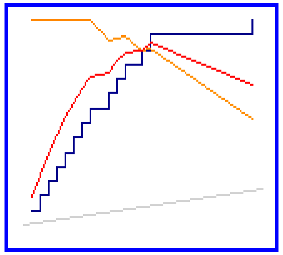	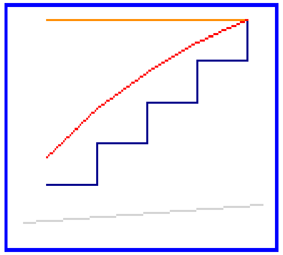	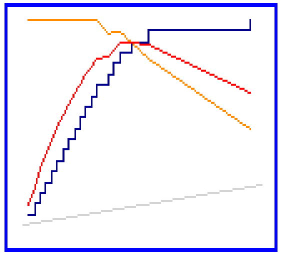	5. Eileiterprüfung<br>(14,5,19)<br>k_XERROR_1<br>Ginni_TRN: 94<br>Ginni_VAL: 94<br>Ginni__FT: 94	Intercept(-2.57,-2.79)<br>k_1_5(5.31,3.59)<br>k_1_37(-3.87,-2.90)	Intercept(-2.74,-3.18)<br>k_1_5(5.59,3.84)<br>k_1_37(-4.24,-3.23)	TRN:(100,87,87)<br>VAL:(100,100,100)<br>FT:(100,89,82)	TRN:(57,93,93)<br>VAL:(100,100,1<br>FT:(63,89,95)
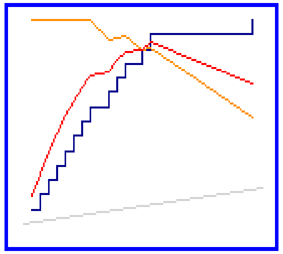	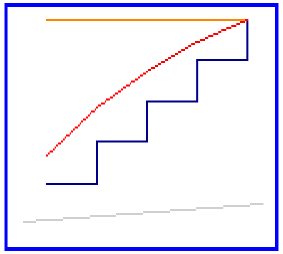	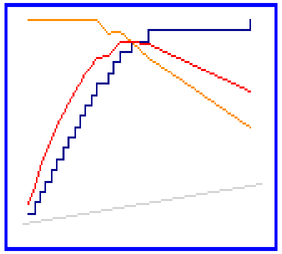	5. Eileiterprüfung<br>(14,5,19)<br>k_XERROR_1<br>Ginni_TRN: 94<br>Ginni_VAL: 94<br>Ginni__FT: 94	Intercept(-2.57,-2.79)<br>k_1_5(5.31,3.59)<br>k_1_37(-3.87,-2.90)	Intercept(-2.74,-3.18)<br>k_1_5(5.59,3.84)<br>k_1_37(-4.24,-3.23)	TRN:(100,87,87)<br>VAL:(100,100,100)<br>FT:(100,89,82)	TRN:(57,93,93)<br>VAL:(100,100,1<br>FT:(63,89,95)
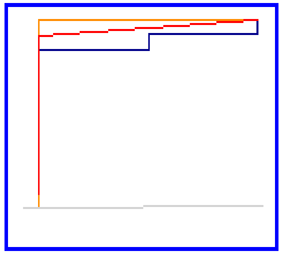	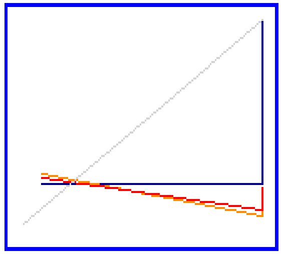	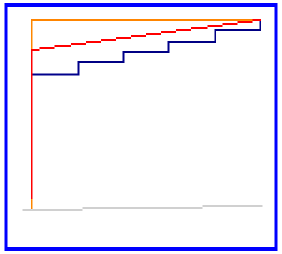	5. Eileiterprüfung<br>(14,5,19)<br>svd_XMISC_40<br>Ginni_TRN: 92<br>Ginni_VAL: 18<br>Ginni__FT: 93	Intercept(-2141.3,-29.13)<br>s8(-4730.9,-0.77)<br>s25(5838.32,2.07)<br>s74(-7129.5,.)<br>s170(-14406,.)<br>s182(10596.8,.)<br>s216(14265.3,.)<br>s225(-7982.8,.)	Intercept(-206.25,-0.02)<br>s2(-531.53,-0.00)<br>s8(-831.30,.)<br>s25(566.78,0.02)<br>s39(-454.00,-0.01)<br>s170(-954.27,.)<br>s177(-602.13,-0.01)<br>s182(696.56,.)<br>s211(841.75,0.11)<br>s216(614.51,0.01)	TRN:(100,100,100)<br>VAL:(25,25,25)<br>FT:(100,100,100)	TRN:(100,100,1<br>VAL:(20,20,20)<br>FT:(95,100,100

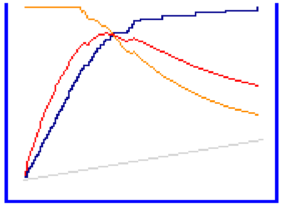	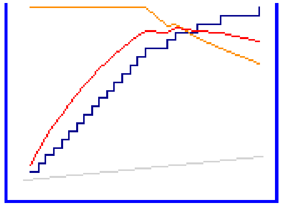	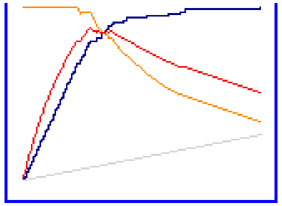	[illegible]<br>(60,21,81)<br>pc_SBC_40<br>Ginni_TRN: 94<br>Ginni_VAL: 95<br>Ginni__FT: 95	[illegible]<br>pC_6_1(2.24,6.35)<br>pC_6_2(-9.13,-5.10)<br>pC_6_3(11.44,3.52)<br>pC_6_7(19.61,2.58)<br>pC_20_3(-5.97,-4.19)<br>pC_26_1(-0.61,-3.61)<br>pC_28_2(5.98,3.14)<br>pC_30_6(-11.85,-3.22)<br>pC_35_5(12.24,2.52)	[illegible]<br>pC_6_1(2.40,7.12)<br>pC_6_2(-10.10,-6.13)<br>pC_6_3(11.81,4.09)<br>pC_6_7(32.13,4.19)<br>pC_14_7(33.60,3.07)<br>pC_16_5(-10.62,-2.96)<br>pC_20_3(-4.38,-3.69)<br>pC_26_1(-0.60,-3.85)<br>pC_28_2(4.52,2.68)	[illegible]<br>VAL:(100,90,68)<br>FT:(100,97,72)	[illegible]<br>VAL:(62,86,100<br>FT:(68,80,94)
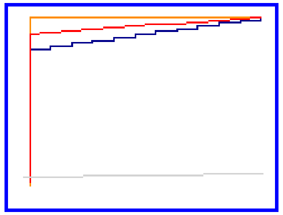	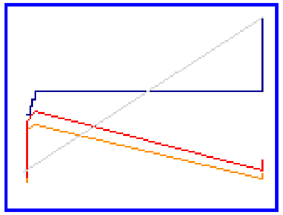	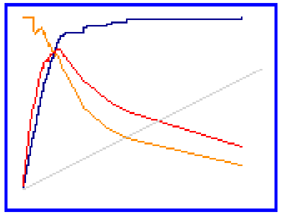	6. Eisprung<br>(60,21,81)<br>svd_XMISC_40<br>Ginni_TRN: 92<br>Ginni_VAL: 50<br>Ginni__FT: 93	Intercept(-4645.9,-0.82)<br>s2(15767.7,0.40)<br>s11(18677.2,0.34)<br>s18(-7433.3,-0.22)<br>s26(-5812.2,.)<br>s28(4780.62,0.12)<br>s41(8484.42,.)<br>s46(-9892.1,.)<br>s53(6797.73,.)<br>s60(5718.22,.)<br>s64(-6337.7,.)<br>s67(-7900.9,.)<br>s88(12607.8,0.55)<br>s106(12969.4,0.13)<br>s139(-7976.6,.)<br>s153(10098.5,.)<br>s195(-12020,-0.08)<br>s198(-10994,.)<br>s207(-6519.8,.)<br>s217(3796.37,0.05)<br>s225(-10069,-0.16)<br>s241(3438.19,0.08)<br>s246(-3010.7,.)<br>s259(3756.87,.)<br>s307(-1697.5,.)<br>s330(2359.89,.)<br>s345(-4170.1,.)<br>s387(4313.77,.)<br>s397(3304.44,0.12)<br>s398(-3325.8,-0.06)<br>s407(-4227.8,.)<br>s425(-4991.1,-0.21)<br>s469(5789.33,.)<br>s482(10461.4,.)<br>s492(-4046.2,.)	Intercept(-7.11,-9.06)<br>s2(21.34,7.22)<br>s8(-12.59,-4.18)<br>s11(19.56,5.79)<br>s14(-14.98,-4.97)<br>s26(-11.52,-4.22)<br>s40(9.12,2.85)<br>s53(13.82,4.03)<br>s64(-9.44,-2.99)<br>s67(-13.01,-3.99)<br>s88(12.68,3.57)<br>s90(-9.19,-2.91)<br>s106(11.65,3.40)<br>s121(10.76,2.93)<br>s139(-12.54,-3.63)<br>s165(15.81,4.36)<br>s198(-16.08,-4.01)<br>s216(12.06,3.01)<br>s225(-10.20,-2.91)<br>s227(-9.64,-2.55)<br>s241(6.28,3.94)<br>s262(-5.23,-3.20)<br>s330(7.03,3.83)<br>s387(7.21,3.95)<br>s394(-5.59,-3.22)<br>s421(6.29,3.03)<br>s425(-6.85,-3.31)<br>s482(12.34,4.86)	TRN:(100,100,100)<br>VAL:(38,38,38)<br>FT:(100,78,67)	TRN:(100,100,1<br>VAL:(57,57,57)<br>FT:(28,85,91)
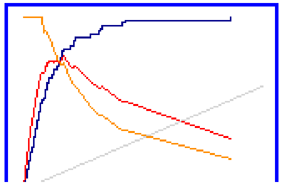	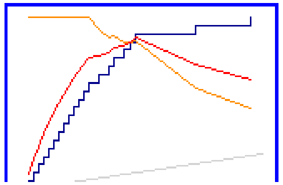	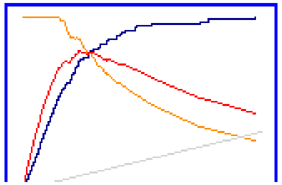	6. Eisprung<br>(60,21,81)<br>k_SBC_30<br>Ginni_TRN: 93<br>Ginni_VAL: 94<br>Ginni__FT: 94	Intercept(-2.35,-5.10)<br>k_30_6(10.59,6.30)<br>k_10_35(-1.09,-2.69)<br>k_5_38(1.57,3.67)<br>k_2_6(-8.13,-4.93)	Intercept(-2.14,-4.80)<br>k_30_6(11.20,7.07)<br>k_30_26(-1.53,-2.76)<br>k_30_31(-0.86,-2.57)<br>k_5_12(-1.52,-2.65)<br>k_5_38(1.72,3.63)<br>k_2_6(-7.66,-4.91)	TRN:(100,73,44)<br>VAL:(100,86,86)<br>FT:(100,87,55)	TRN:(50,82,95)<br>VAL:(62,90,90)<br>FT:(53,75,95)

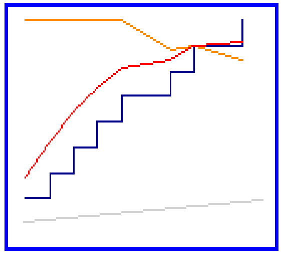	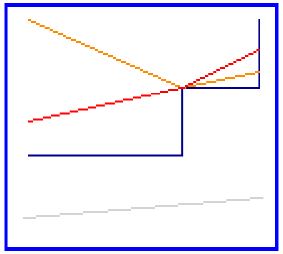	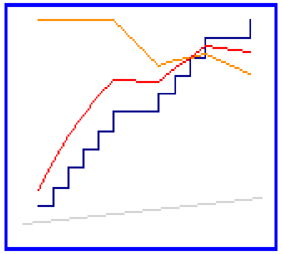	7. Endometriose<br>(8,3,11)<br>k_XERROR_10<br>Ginni_TRN: 94<br>Ginni_VAL: 91<br>Ginni__FT: 94	Intercept(-5.31,-4.09)<br>k_1_7(2.43,3.20)	Intercept(-5.47,-4.64)<br>k_1_7(2.35,4.14)	TRN:(100,80,80)<br>VAL:(100,75,75)<br>FT:(100,83,73)	TRN:(63,100,10<br>VAL:(33,100,10<br>FT:(55,91,100)
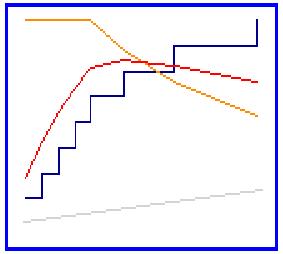	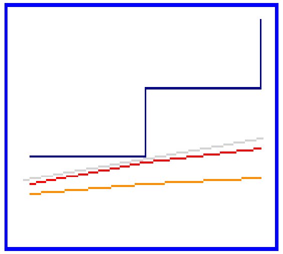	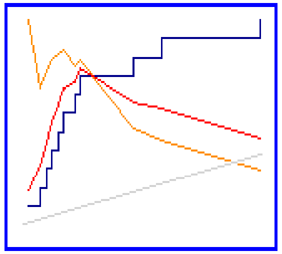	7. Endometriose<br>(8,3,11)<br>svd_XMISC_40<br>Ginni_TRN: 93<br>Ginni_VAL: 68<br>Ginni__FT: 90	Intercept(-9.46,-2.26)<br>s6(51.23,2.02)<br>s66(33.99,2.27)<br>s78(-53.26,-1.90)	Intercept(-4.39,-4.84)<br>s6(16.30,3.04)<br>s66(26.54,2.90)<br>s84(26.74,2.68)	TRN:(100,86,53)<br>VAL:(23,23,23)<br>FT:(100,80,42)	TRN:(63,75,100<br>VAL:(100,100,1<br>FT:(9,73,91)
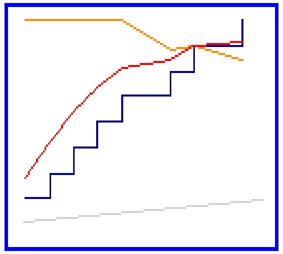	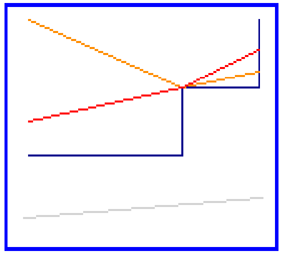	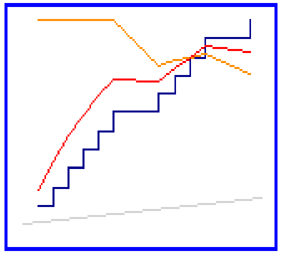	7. Endometriose<br>(8,3,11)<br>k_XERROR_10<br>Ginni_TRN: 94<br>Ginni_VAL: 91<br>Ginni__FT: 94	Intercept(-5.31,-4.09)<br>k_1_7(2.43,3.20)	Intercept(-5.47,-4.64)<br>k_1_7(2.35,4.14)	TRN:(100,80,80)<br>VAL:(100,75,75)<br>FT:(100,83,73)	TRN:(63,100,10<br>VAL:(33,100,10<br>FT:(55,91,100)

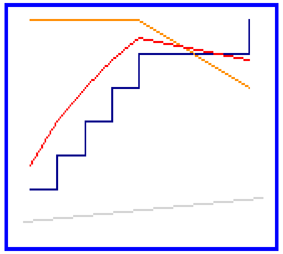	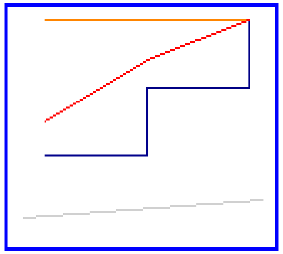	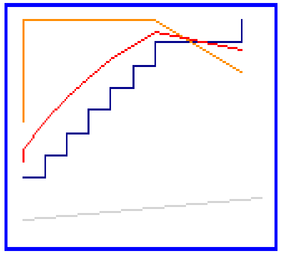	8. Ernährung<br>(6,3,9)<br>k_XMISC_1<br>Ginni_TRN: 95<br>Ginni_VAL: 92<br>Ginni__FT: 95	Intercept(-5.32,-2.03)<br>k_1_8(22.75,1.35)	Intercept(-5.49,-2.08)<br>k_1_8(23.68,1.38)	TRN:(100,100,67)<br>VAL:(100,100,100)<br>FT:(100,100,75)	TRN:(83,83,100<br>VAL:(100,100,1<br>FT:(89,89,100)
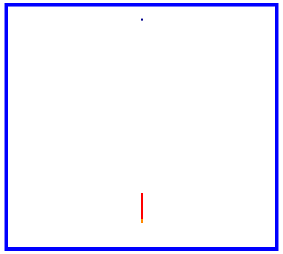	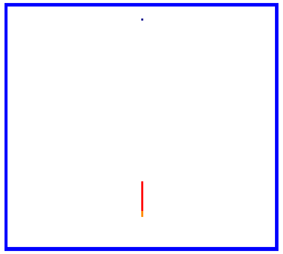	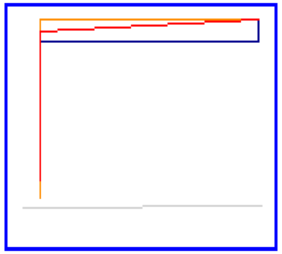	8. Ernährung<br>(6,3,9)<br>k_XERROR_10<br>Ginni_TRN: 0<br>Ginni_VAL: 0<br>Ginni__FT: 92	Intercept(-2.41,-5.66)	Intercept(-26.42,-0.32)<br>k_2_8(113.27,0.27)	TRN:(8,8,8)<br>VAL:(12,12,12)<br>FT:(100,100,100)	TRN:(100,100,1<br>VAL:(100,100,1<br>FT:(100,100,10
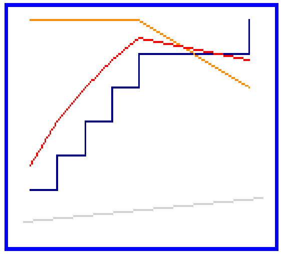	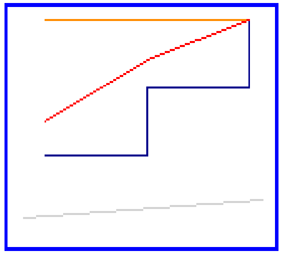	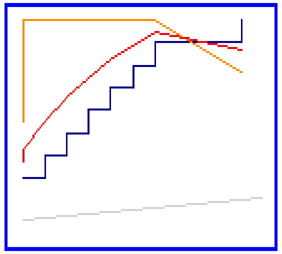	8. Ernährung<br>(6,3,9)<br>k_XMISC_1<br>Ginni_TRN: 95<br>Ginni_VAL: 92<br>Ginni__FT: 95	Intercept(-5.32,-2.03)<br>k_1_8(22.75,1.35)	Intercept(-5.49,-2.08)<br>k_1_8(23.68,1.38)	TRN:(100,100,67)<br>VAL:(100,100,100)<br>FT:(100,100,75)	TRN:(83,83,100<br>VAL:(100,100,1<br>FT:(89,89,100)

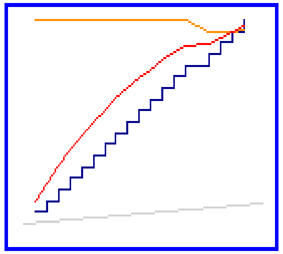	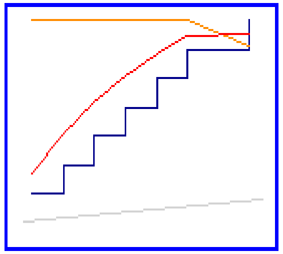	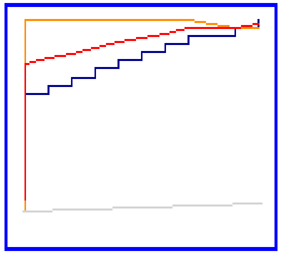	9. Gelbkörperschwäche<br>(18,7,25)<br>pc_SBC_40<br>Ginni_TRN: 95<br>Ginni_VAL: 94<br>Ginni__FT: 93	Intercept(-16.10,-1.67)<br>pC_9_1(2.94,1.73)<br>pC_29_4(39.30,1.53)	Intercept(-53.10,-0.80)<br>pC_4_4(280.91,0.75)<br>pC_9_1(11.17,0.78)<br>pC_18_2(-14.91,-0.96)	TRN:(100,95,95)<br>VAL:(100,88,88)<br>FT:(100,96,96)	TRN:(72,100,10<br>VAL:(86,100,10<br>FT:(92,100,100
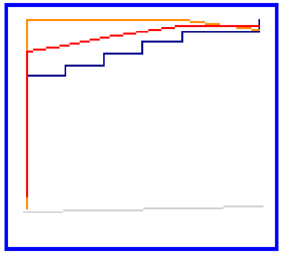	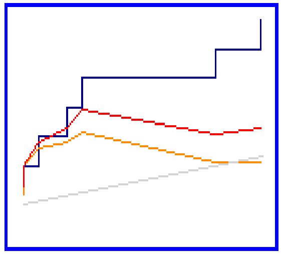	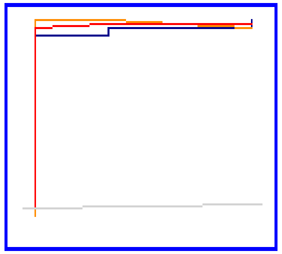	9. Gelbkörperschwäche<br>(18,7,25)<br>svd_XMISC_40<br>Ginni_TRN: 93<br>Ginni_VAL: 82<br>Ginni__FT: 92	Intercept(-583.73,-0.52)<br>s2(2149.29,.)<br>s17(891.25,.)<br>s31(-2177.4,.)<br>s55(-614.91,-0.04)<br>s64(-1799.9,.)<br>s75(-2467.7,.)<br>s152(-1634.8,-0.02)<br>s182(2807.59,0.04)<br>s193(2034.41,.)	Intercept(-3104.8,-4.19)<br>s2(12633.8,.)<br>s9(6668.95,.)<br>s14(8430.21,0.11)<br>s31(-6344.1,.)<br>s48(6756.92,.)<br>s60(-9964.4,.)<br>s75(-7458.2,.)<br>s101(-10482,.)<br>s110(6842.81,.)<br>s182(7787.80,0.09)	TRN:(100,95,95)<br>VAL:(45,45,30)<br>FT:(100,96,96)	TRN:(83,100,10<br>VAL:(71,71,100<br>FT:(92,100,100
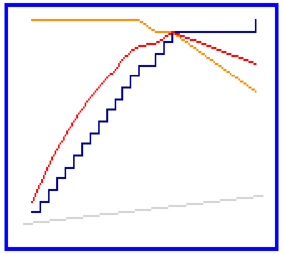	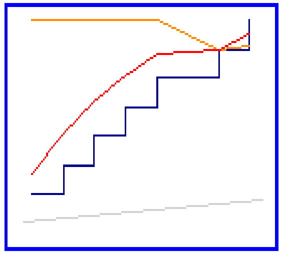	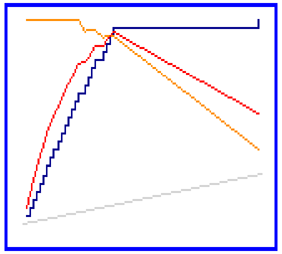	9. Gelbkörperschwäche<br>(18,7,25)<br>k_XERROR_1<br>Ginni_TRN: 95<br>Ginni_VAL: 94<br>Ginni__FT: 94	Intercept(-4.31,-2.83)<br>k_1_9(8.47,3.65)<br>k_1_37(-2.67,-1.93)	Intercept(-3.70,-4.01)<br>k_1_9(6.26,4.61)<br>k_1_38(-3.75,-2.55)	TRN:(100,94,64)<br>VAL:(100,88,88)<br>FT:(100,92,92)	TRN:(72,94,100<br>VAL:(71,100,10<br>FT:(64,96,96)

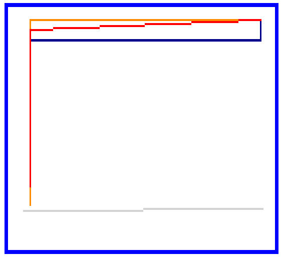	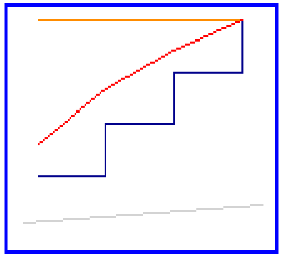	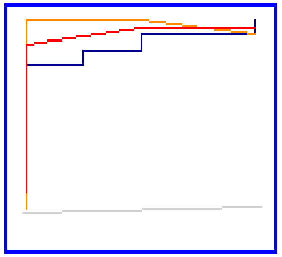	10. Geschlechtsverkehr<br>(10,4,14)<br>k_AIC_1<br>Ginni_TRN: 92<br>Ginni_VAL: 94<br>Ginni__FT: 93	Intercept(-41.80,-0.34)<br>k_1_10(71.09,0.31)	Intercept(-41.30,-1.00)<br>k_1_10(81.32,0.99)	TRN:(100,100,100)<br>VAL:(100,100,100)<br>FT:(100,93,93)	TRN:(100,100,1<br>VAL:(100,100,1<br>FT:(93,100,100
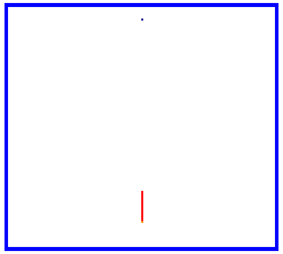	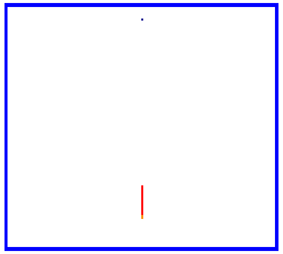	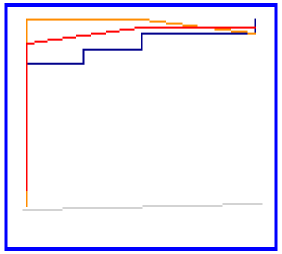	10. Geschlechtsverkehr<br>(10,4,14)<br>k_XERROR_1<br>Ginni_TRN: 0<br>Ginni_VAL: 0<br>Ginni__FT: 93	Intercept(-2.35,-7.11)	Intercept(-41.30,-1.00)<br>k_1_10(81.32,0.99)	TRN:(9,9,9)<br>VAL:(10,10,10)<br>FT:(100,93,93)	TRN:(100,100,1<br>VAL:(100,100,1<br>FT:(93,100,100
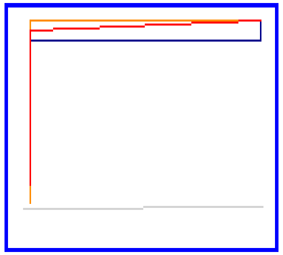	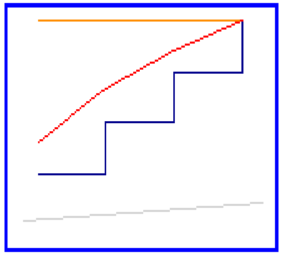	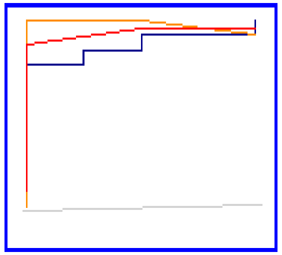	10. Geschlechtsverkehr<br>(10,4,14)<br>k_AIC_1<br>Ginni_TRN: 92<br>Ginni_VAL: 94<br>Ginni__FT: 93	Intercept(-41.80,-0.34)<br>k_1_10(71.09,0.31)	Intercept(-41.30,-1.00)<br>k_1_10(81.32,0.99)	TRN:(100,100,100)<br>VAL:(100,100,100)<br>FT:(100,93,93)	TRN:(100,100,1<br>VAL:(100,100,1<br>FT:(93,100,100

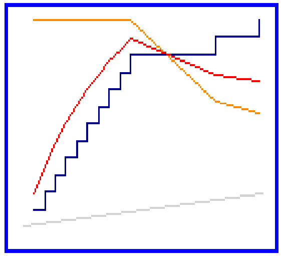	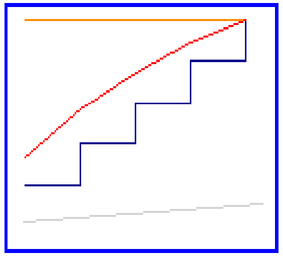	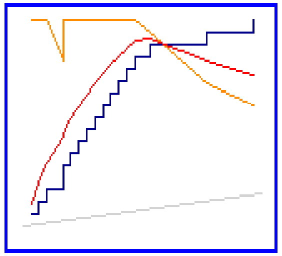	11. Habituell<br>(12,5,17)<br>k_XERROR_20<br>Ginni_TRN: 94<br>Ginni_VAL: 94<br>Ginni__FT: 95	Intercept(-7.04,-4.01)<br>k_20_11(3.88,3.51)	Intercept(-8.04,-4.20)<br>k_10_11(4.69,3.82)	TRN:(100,100,55)<br>VAL:(100,100,100)<br>FT:(100,94,59)	TRN:(83,83,100<br>VAL:(100,100,1<br>FT:(82,88,100)
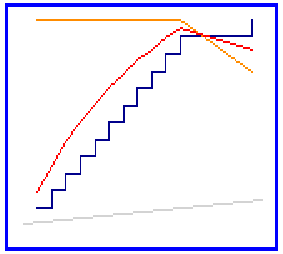	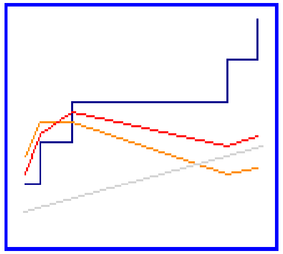	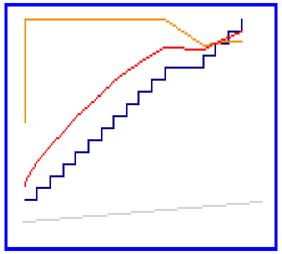	11. Habituell<br>(12,5,17)<br>svd_XMISC_40<br>Ginni_TRN: 95<br>Ginni_VAL: 80<br>Ginni__FT: 95	Intercept(-31.07,-1.71)<br>s3(-269.71,-1.73)<br>s21(115.53,1.65)<br>s125(78.68,1.99)<br>s164(-199.87,-1.67)	Intercept(-35.23,-1.58)<br>s3(-273.72,-1.54)<br>s20(-100.41,-1.59)<br>s23(-111.62,-1.59)<br>s49(89.60,1.59)<br>s170(-125.30,-1.55)<br>s196(181.68,1.58)	TRN:(100,100,75)<br>VAL:(50,50,28)<br>FT:(100,89,89)	TRN:(83,92,100<br>VAL:(60,60,100<br>FT:(71,100,100)
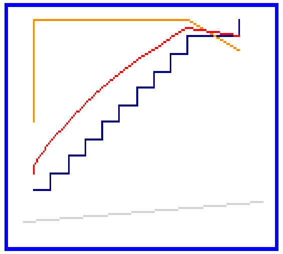	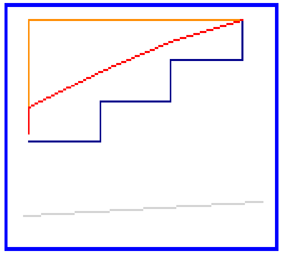	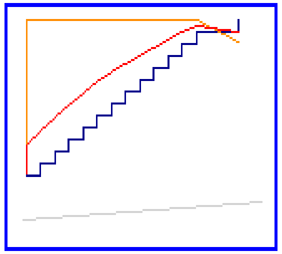	11. Habituell<br>(12,5,17)<br>k_AIC_1<br>Ginni_TRN: 95<br>Ginni_VAL: 93<br>Ginni__FT: 95	Intercept(-12.45,-1.42)<br>k_1_11(27.28,1.28)<br>k_1_38(-17.48,-1.20)	Intercept(-12.45,-1.42)<br>k_1_11(27.28,1.29)<br>k_1_38(-17.48,-1.20)	TRN:(100,100,86)<br>VAL:(100,100,100)<br>FT:(100,100,89)	TRN:(83,92,100<br>VAL:(100,100,1<br>FT:(82,94,100)

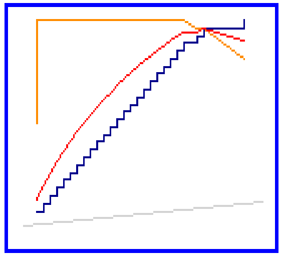	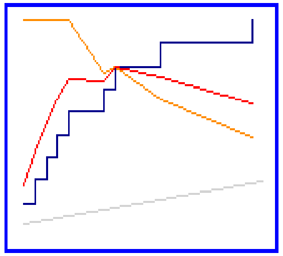	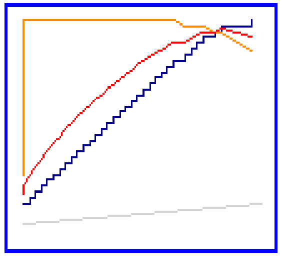	12. Hormone<br>(27,9,36)<br>k_SBC_40<br>Ginni_TRN: 95<br>Ginni_VAL: 93<br>Ginni__FT: 95	Intercept(-4.99,-1.63)<br>k_40_12(16.22,2.23)<br>k_20_35(-3.14,-2.12)<br>k_20_37(-54.00,-1.89)<br>k_10_37(47.60,1.79)	Intercept(-9.84,-2.61)<br>k_40_12(65.04,2.94)<br>k_30_33(6.55,2.91)<br>k_20_6(-3.81,-2.67)<br>k_20_12(-50.87,-2.83)<br>k_20_22(-10.88,-2.52)	TRN:(100,96,82)<br>VAL:(100,78,43)<br>FT:(100,95,86)	TRN:(89,96,100<br>VAL:(44,78,100<br>FT:(81,97,100)
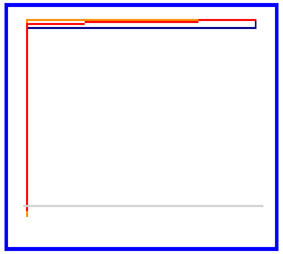	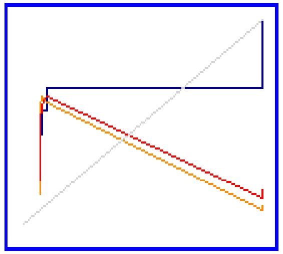	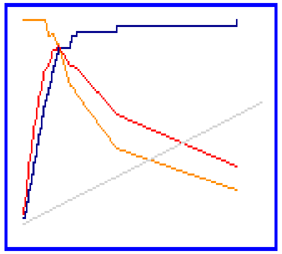	12. Hormone<br>(27,9,36)<br>k_XMISC_10<br>Ginni_TRN: 91<br>Ginni_VAL: 62<br>Ginni__FT: 93	Intercept(-621.29,-2.68)<br>k_10_6(-677.24,.)<br>k_10_12(2147.10,.)<br>k_10_18(-1189.6,-9.44)<br>k_10_38(-887.30,.)<br>k_5_30(-1151.6,-2.49)<br>k_5_35(-807.89,.)	Intercept(-5.33,-4.57)<br>k_10_12(7.58,5.66)<br>k_10_21(-1.20,-1.77)<br>k_10_33(2.89,3.43)<br>k_2_6(-2.40,-2.99)<br>k_2_22(-3.88,-3.07)	TRN:(100,100,100)<br>VAL:(63,60,60)<br>FT:(100,89,64)	TRN:(96,100,10<br>VAL:(56,67,67)<br>FT:(61,86,94)
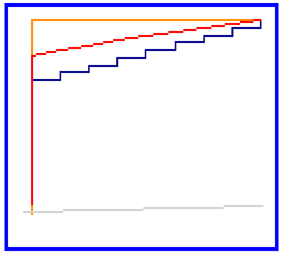	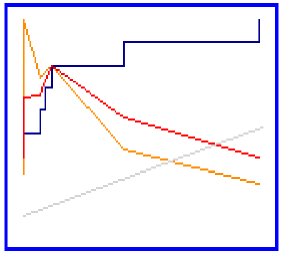	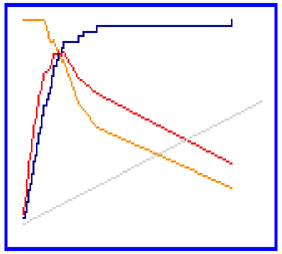	12. Hormone<br>(27,9,36)<br>k_SBC_10<br>Ginni_TRN: 93<br>Ginni_VAL: 88<br>Ginni__FT: 93	Intercept(-79.50,-0.40)<br>k_10_6(-60.50,-0.37)<br>k_10_12(197.41,0.34)<br>k_10_35(175.68,0.25)<br>k_10_38(-87.25,-0.32)<br>k_5_35(-259.34,-0.26)<br>k_2_30(-131.40,-0.33)	Intercept(-4.49,-4.72)<br>k_10_12(7.10,5.88)<br>k_10_33(3.45,4.07)<br>k_2_6(-2.08,-2.86)<br>k_2_22(-3.69,-3.13)	TRN:(100,100,100)<br>VAL:(100,78,78)<br>FT:(100,86,56)	TRN:(96,100,10<br>VAL:(44,78,78)<br>FT:(58,83,94)

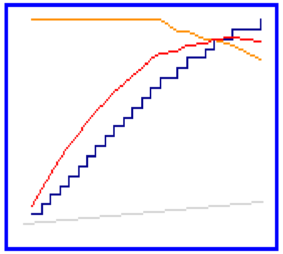	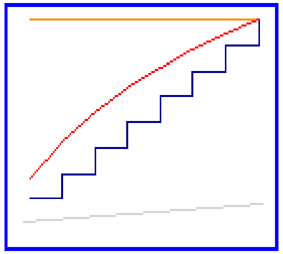	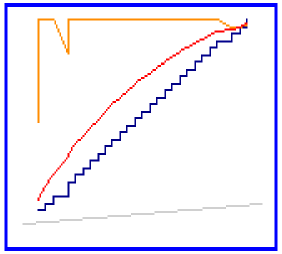	13. Insemination<br>(21,8,29)<br>k_XERROR_1<br>Ginni_TRN: 95<br>Ginni_VAL: 94<br>Ginni__FT: 95	Intercept(-8.89,-3.90)<br>k_1_13(9.87,3.84)	Intercept(-18.70,-2.06)<br>k_1_13(32.59,2.05)<br>k_1_37(-7.32,-1.79)	TRN:(100,87,81)<br>VAL:(100,100,100)<br>FT:(100,97,97)	TRN:(52,95,100<br>VAL:(100,100,1<br>FT:(86,100,100)
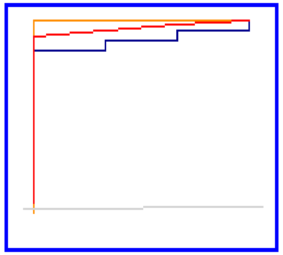	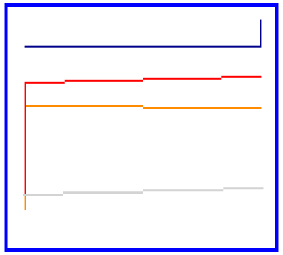	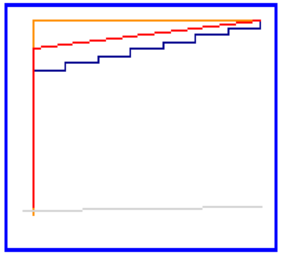	13. Insemination<br>(21,8,29)<br>svd_XMISC_40<br>Ginni_TRN: 92<br>Ginni_VAL: 85<br>Ginni__FT: 93	Intercept(-1480.5,-3.95)<br>s5(-6389.6,-0.42)<br>s17(6562.10,.)<br>s58(5682.77,.)<br>s75(4781.96,1.85)<br>s89(-6250.4,.)<br>s115(5857.87,0.67)<br>s189(6472.06,.)<br>s481(-1364.9,-0.68)<br>s489(2461.29,.)	Intercept(-1045.8,-2.08)<br>s5(-2494.8,.)<br>s17(4487.26,.)<br>s20(2883.10,0.23)<br>s52(3362.30,0.20)<br>s53(-1554.3,-0.13)<br>s61(1964.15,.)<br>s70(-3101.8,-0.68)<br>s103(4008.38,.)<br>s110(4920.88,0.34)<br>s116(2923.71,0.32)<br>s174(1728.99,0.22)<br>s360(-1179.6,-0.45)	TRN:(100,100,100)<br>VAL:(58,57,57)<br>FT:(100,100,100)	TRN:(100,100,1<br>VAL:(88,100,10<br>FT:(86,100,100)
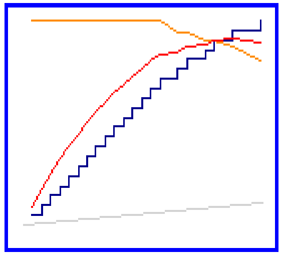	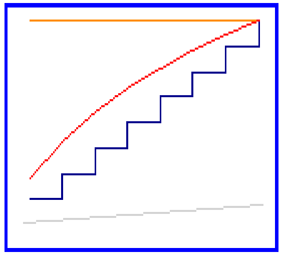	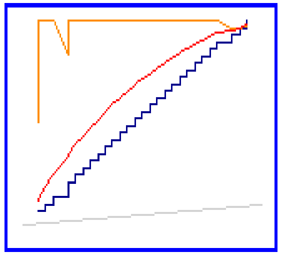	13. Insemination<br>(21,8,29)<br>k_XERROR_1<br>Ginni_TRN: 95<br>Ginni_VAL: 94<br>Ginni__FT: 95	Intercept(-8.89,-3.90)<br>k_1_13(9.87,3.84)	Intercept(-18.70,-2.06)<br>k_1_13(32.59,2.05)<br>k_1_37(-7.32,-1.79)	TRN:(100,87,81)<br>VAL:(100,100,100)<br>FT:(100,97,97)	TRN:(52,95,100<br>VAL:(100,100,1<br>FT:(86,100,100)

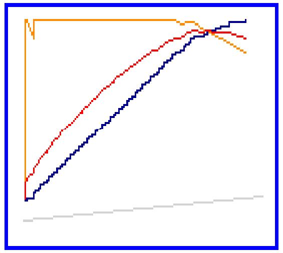	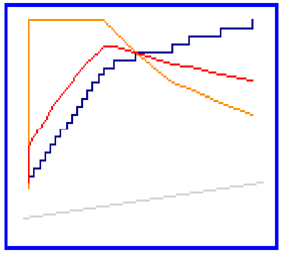	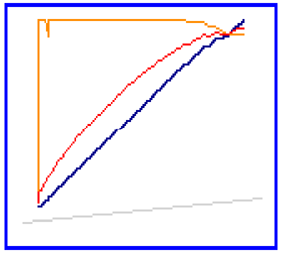	14. lvf<br>(76,26,102)<br>pc_XERROR_40<br>Ginni_TRN: 95<br>Ginni_VAL: 93<br>Ginni__FT: 95	Intercept(-25.75,-3.90)<br>pC_5_7(-112.29,-3.36)<br>pC_7_6(39.74,3.08)<br>pC_7_7(-61.32,-2.94)<br>pC_14_1(8.48,3.80)<br>pC_14_2(-21.54,-3.99)<br>pC_16_1(-1.77,-3.07)<br>pC_31_2(-14.18,-3.56)<br>pC_32_1(-1.30,-2.97)	Intercept(-23.66,-4.35)<br>pC_5_7(-56.80,-3.36)<br>pC_14_1(7.39,4.29)<br>pC_14_2(-23.56,-4.09)<br>pC_14_6(57.99,3.28)<br>pC_16_1(-0.90,-2.83)<br>pC_17_4(15.40,2.99)<br>pC_18_4(10.42,2.43)<br>pC_24_7(28.02,2.33)<br>pC_28_5(28.65,2.28)<br>pC_31_2(-11.85,-3.84)<br>pC_32_1(-1.54,-3.42)<br>pC_32_5(16.82,2.17)	TRN:(100,99,84)<br>VAL:(100,95,54)<br>FT:(100,93,93)	TRN:(84,92,100<br>VAL:(69,81,100<br>FT:(61,100,100)
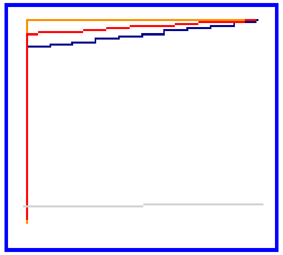	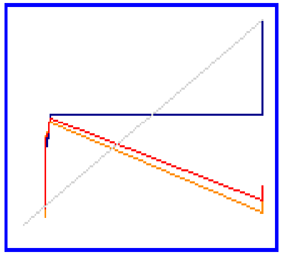	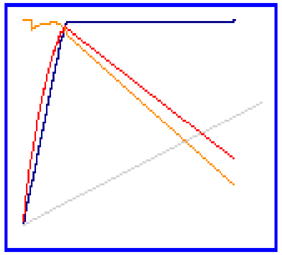	14. lvf<br>(76,26,102)<br>svd_XMISC_40<br>Ginni_TRN: 91<br>Ginni_VAL: 49<br>Ginni__FT: 94	Intercept(-4295.1,-2.40)<br>s2(-9851.2,.) s4(-10832,.)<br>s7(-12565,.)<br>s8(11074.1,2.46)<br>s15(-5500.1,-1.33)<br>s43(2582.61,.)<br>s45(7412.13,.)<br>s56(-4708.5,-0.30)<br>s77(-4359.2,-0.25)<br>s93(-2688.8,.)<br>s139(6442.54,.)<br>[illegible]	Intercept(-19.41,-6.35)<br>s2(-47.31,-6.11)<br>s4(-21.21,-4.24)<br>s5(-22.92,-4.23)<br>s6(-39.15,-5.19)<br>s7(-34.03,-5.21)<br>s8(42.90,5.55)<br>s12(38.56,4.76)<br>s22(-31.76,-5.36)<br>s23(19.67,3.82)<br>s45(40.76,5.10)<br>[illegible]	TRN:(100,100,100)<br>VAL:(50,50,50)<br>FT:(100,95,94)	TRN:(100,100,1<br>VAL:(54,54,54)<br>FT:(20,98,99)

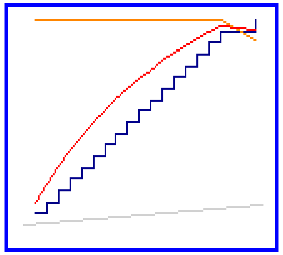	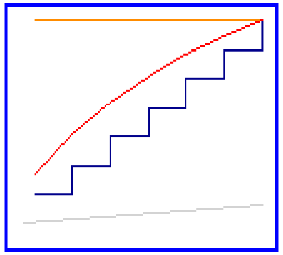	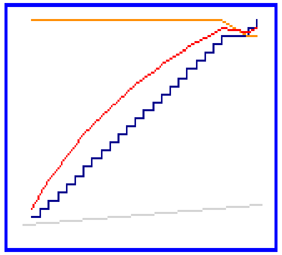	15. Kosten<br>(18,7,25)<br>k_XERROR_1<br>Ginni_TRN: 95<br>Ginni_VAL: 94<br>Ginni__FT: 95	Intercept(-2.92,-1.40)<br>k_1_15(3.93,3.09)<br>k_1_33(5.87,1.18)	Intercept(-2.25,-1.31)<br>k_1_15(4.11,3.41)<br>k_1_33(6.37,1.46)	TRN:(100,100,90)<br>VAL:(100,100,100)<br>FT:(100,93,93)	TRN:(83,94,100<br>VAL:(100,100,1<br>FT:(92,100,100
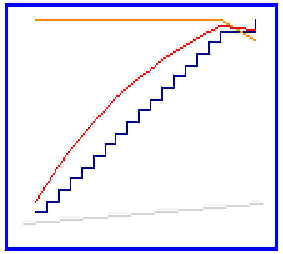	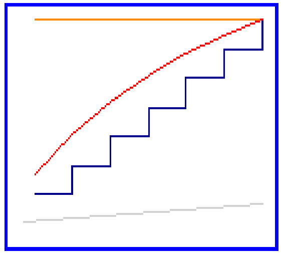	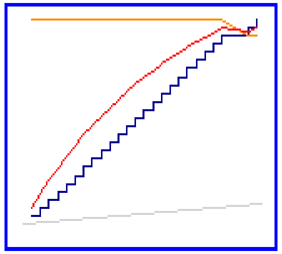	15. Kosten<br>(18,7,25)<br>k_XERROR_1<br>Ginni_TRN: 95<br>Ginni_VAL: 94<br>Ginni__FT: 95	Intercept(-2.92,-1.40)<br>k_1_15(3.93,3.09)<br>k_1_33(5.87,1.18)	Intercept(-2.25,-1.31)<br>k_1_15(4.11,3.41)<br>k_1_33(6.37,1.46)	TRN:(100,100,90)<br>VAL:(100,100,100)<br>FT:(100,93,93)	TRN:(83,94,100<br>VAL:(100,100,1<br>FT:(92,100,100
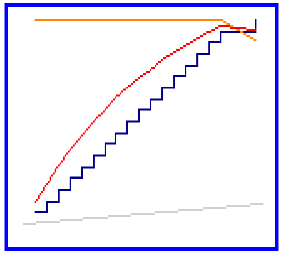	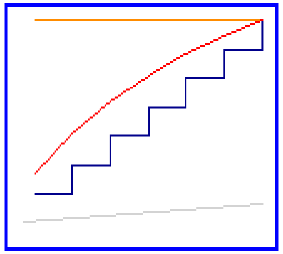	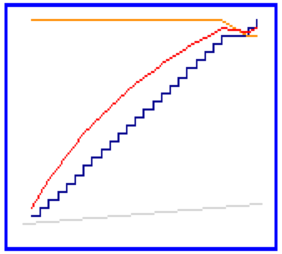	15. Kosten<br>(18,7,25)<br>k_XERROR_1<br>Ginni_TRN: 95<br>Ginni_VAL: 94<br>Ginni__FT: 95	Intercept(-2.92,-1.40)<br>k_1_15(3.93,3.09)<br>k_1_33(5.87,1.18)	Intercept(-2.25,-1.31)<br>k_1_15(4.11,3.41)<br>k_1_33(6.37,1.46)	TRN:(100,100,90)<br>VAL:(100,100,100)<br>FT:(100,93,93)	TRN:(83,94,100<br>VAL:(100,100,1<br>FT:(92,100,100

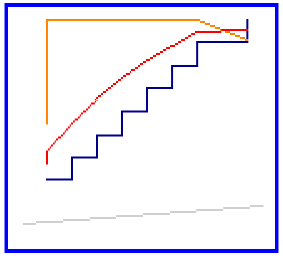	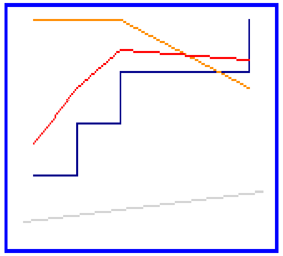	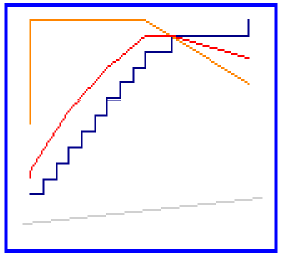	16. Kryotransfer<br>(9,4,13)<br>k_XERROR_1<br>Ginni_TRN: 95<br>Ginni_VAL: 92<br>Ginni__FT: 95	Intercept(-14.24,-1.13)<br>k_1_16(14.69,1.05)	Intercept(-7.87,-3.20)<br>k_1_16(8.07,2.43)	TRN:(100,90,90)<br>VAL:(100,100,67)<br>FT:(100,92,68)	TRN:(89,100,10<br>VAL:(75,75,100<br>FT:(85,92,100)
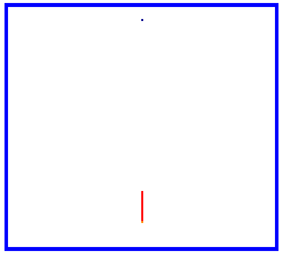	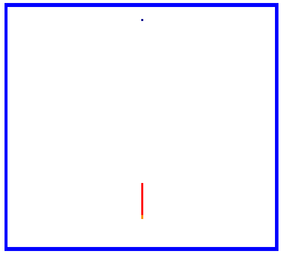	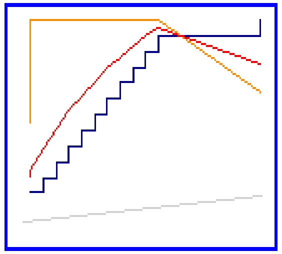	16. Kryotransfer<br>(9,4,13)<br>k_XERROR_10<br>Ginni_TRN: 0<br>Ginni_VAL: 0<br>Ginni__FT: 95	Intercept(-2.38,-6.82)	Intercept(-7.81,-3.40)<br>k_2_16(7.45,2.61)	TRN:(8,8,8)<br>VAL:(11,11,11)<br>FT:(100,100,65)	TRN:(100,100,1<br>VAL:(100,100,1<br>FT:(92,92,100)
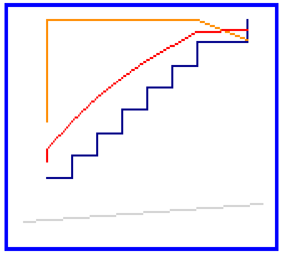	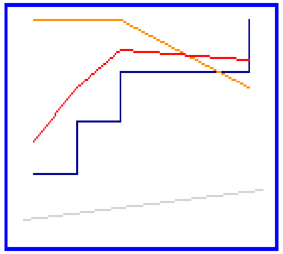	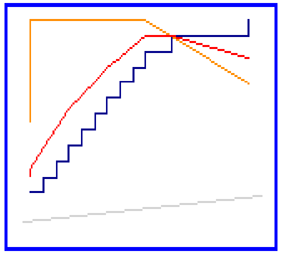	16. Kryotransfer<br>(9,4,13)<br>k_XERROR_1<br>Ginni_TRN: 95<br>Ginni_VAL: 92<br>Ginni__FT: 95	Intercept(-14.24,-1.13)<br>k_1_16(14.69,1.05)	Intercept(-7.87,-3.20)<br>k_1_16(8.07,2.43)	TRN:(100,90,90)<br>VAL:(100,100,67)<br>FT:(100,92,68)	TRN:(89,100,10<br>VAL:(75,75,100<br>FT:(85,92,100)

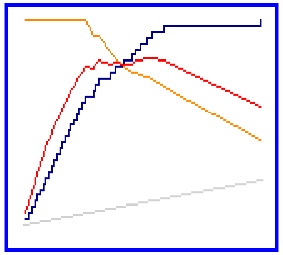	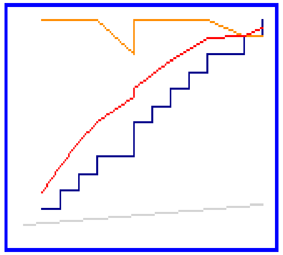	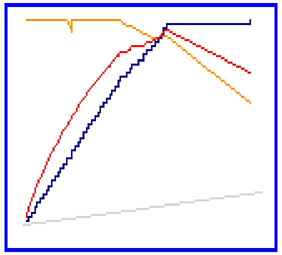	17. Medikamente<br>(35,12,47)<br>k_SBC_30<br>Ginni_TRN: 94<br>Ginni_VAL: 95<br>Ginni__FT: 95	Intercept(-3.98,-3.92)<br>k_30_17(18.39,4.44)<br>k_20_32(-3.12,-1.64)<br>k_5_17(-14.42,-3.65)<br>k_5_37(-1.79,-2.80)	Intercept(-6.91,-4.35)<br>k_30_17(35.01,4.24)<br>k_20_7(-8.69,-3.00)<br>k_20_21(-6.00,-2.66)<br>k_10_31(-2.38,-2.72)<br>k_5_17(-27.85,-3.93)<br>k_5_25(-7.06,-3.46)	TRN:(100,72,68)<br>VAL:(100,92,92)<br>FT:(100,92,92)	TRN:(63,94,97)<br>VAL:(83,100,10<br>FT:(72,98,98)
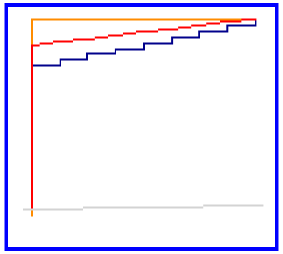	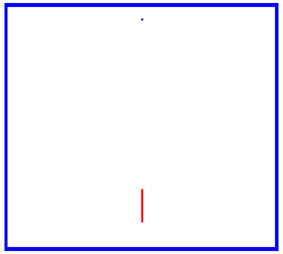	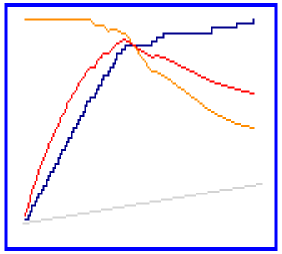	17. Medikamente<br>(35,12,47)<br>svd_XMISC_40<br>Ginni_TRN: 93<br>Ginni_VAL: 0<br>Ginni__FT: 95	Intercept(-2366.0,-52.27)<br>s10(-4402.8,.)<br>s13(4737.47,.)<br>s20(-4138.3,.)<br>s21(-7061.5,-10.96)<br>s34(-6520.0,-3.03)<br>s37(-7303.9,.)<br>s55(5908.25,3.70)<br>s64(-3306.4,-1.98)<br>s110(-1478.1,-1.53)<br>s142(-8348.5,.)<br>s144(-4444.1,.)<br>s176(2695.32,.)<br>s179(10752.7,.)<br>s232(-2126.8,.)<br>s235(4011.42,9.81)<br>s304(1078.80,.)<br>s364(-1961.2,-4.05)<br>s365(-4645.9,.)<br>s370(2760.43,.)<br>s391(-4744.0,.)<br>s416(3597.65,7.23)	Intercept(-22.43,-4.38)<br>s1(26.40,2.89)<br>s5(27.62,4.07)<br>s10(-44.03,-3.96)<br>s11(29.77,3.35)<br>s16(-24.35,-2.57)<br>s18(44.31,3.99)<br>s21(-30.43,-3.79)<br>s48(20.95,3.11)<br>s51(-42.50,-3.69)<br>s66(26.26,3.37)<br>s70(-27.78,-3.56)<br>s123(36.30,3.27)<br>s130(37.40,3.75)<br>s144(-20.59,-2.83)<br>s154(-21.90,-2.77)<br>s161(27.33,3.35)<br>s168(-23.85,-2.59)<br>s179(43.26,3.91)<br>s185(-33.59,-3.42)<br>s200(-32.63,-3.52)<br>s220(-32.43,-3.28)<br>s229(-24.78,-3.93)<br>s236(18.06,3.71)<br>s258(18.73,3.90)<br>s284(-22.60,-3.37)<br>s303(-9.72,-2.55)<br>s391(-20.30,-3.73)<br>s405(12.85,2.92)<br>s464(-32.43,-4.28)<br>s495(-21.31,-3.89)	TRN:(100,100,100)<br>VAL:(9,9,9)<br>FT:(100,93,72)	TRN:(100,100,1<br>VAL:(100,100,1<br>FT:(62,87,94)
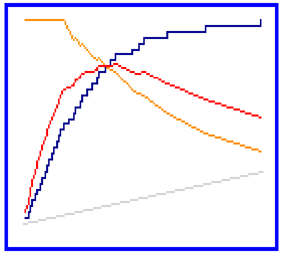	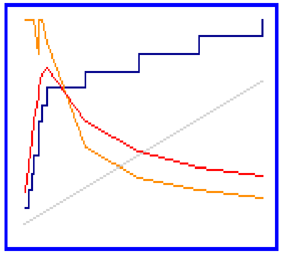	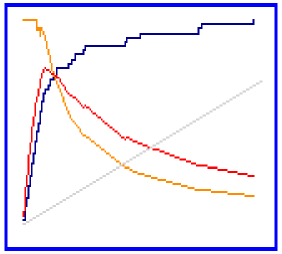	17. Medikamente<br>(35,12,47)<br>k_SBC_2<br>Ginni_TRN: 94<br>Ginni_VAL: 83<br>Ginni__FT: 90	Intercept(-1.55,-1.95)<br>k_2_17(31.08,4.54)<br>k_2_20(-7.09,-2.41)<br>k_2_21(-3.21,-2.13)<br>k_2_33(1.95,2.30)<br>k_2_37(-2.71,-3.29)<br>k_1_17(-22.86,-3.79)<br>k_1_19(-7.82,-3.07)	Intercept(-3.40,-6.03)<br>k_2_17(6.78,7.58)<br>k_2_33(1.86,3.73)<br>k_1_9(-3.18,-2.41)<br>k_1_20(-2.70,-1.82)	TRN:(100,74,54)<br>VAL:(100,89,89)<br>FT:(100,91,43)	TRN:(49,83,94)<br>VAL:(58,67,67)<br>FT:(43,66,87)

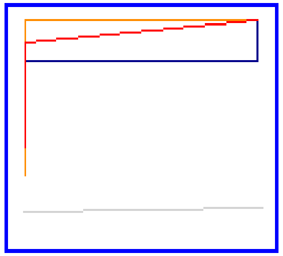	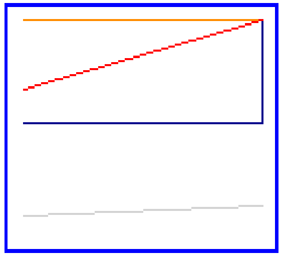	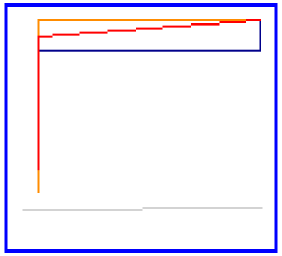	18. Mehrlinge<br>(5,2,7)<br>k_SBC_1<br>Ginni_TRN: 93<br>Ginni_VAL: 93<br>Ginni__FT: 92	Intercept(-24.12,-0.09)<br>k_1_18(24.27,0.11)	Intercept(-67.57,-0.01)<br>k_1_18(63.83,0.01)	TRN:(100,100,100)<br>VAL:(100,100,100)<br>FT:(100,100,100)	TRN:(100,100,1<br>VAL:(100,100,1<br>FT:(100,100,10
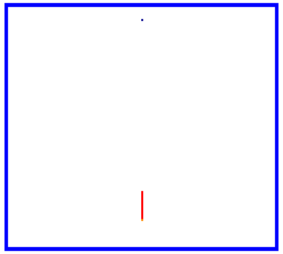	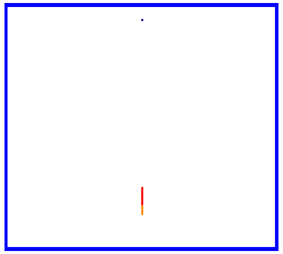	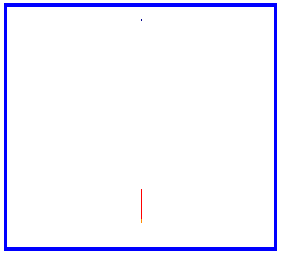	18. Mehrlinge<br>(5,2,7)<br>k_XERROR_1<br>Ginni_TRN: 0<br>Ginni_VAL: 0<br>Ginni__FT: 0	Intercept(-2.34,-5.00)	Intercept(-2.30,-5.81)	TRN:(9,9,9)<br>VAL:(10,10,10)<br>FT:(9,9,9)	TRN:(100,100,1<br>VAL:(100,100,1<br>FT:(100,100,10
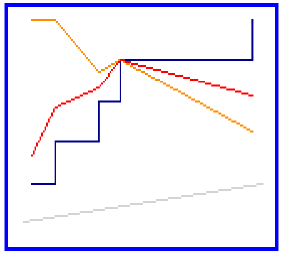	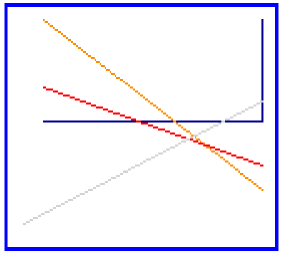	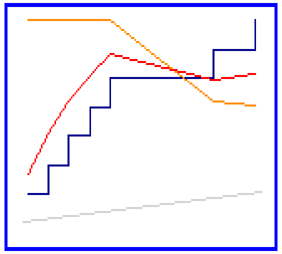	18. Mehrlinge<br>(5,2,7)<br>svd_XERROR_40<br>Ginni_TRN: 92<br>Ginni_VAL: 68<br>Ginni__FT: 93	Intercept(-5.75,-2.83)<br>s65(-38.28,-2.46)<br>s144(-41.55,-2.48)	Intercept(-10.23,-2.30)<br>s50(76.31,2.01)<br>s51(65.37,2.11)	TRN:(100,80,45)<br>VAL:(100,100,100)<br>FT:(100,100,58)	TRN:(40,80,100<br>VAL:(50,50,50)<br>FT:(57,71,100)

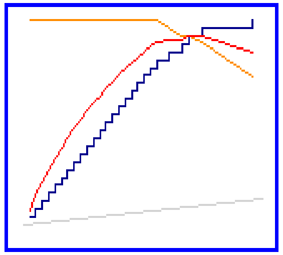	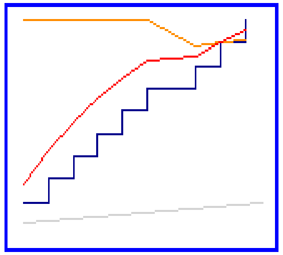	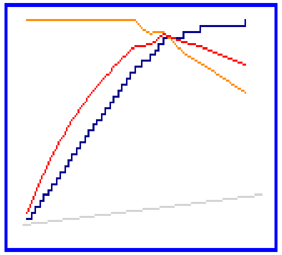	19. Menstruation<br>(26,9,35)<br>k_XERROR_10<br>Ginni_TRN: 95<br>Ginni_VAL: 95<br>Ginni__FT: 95	Intercept(-2.86,-2.13)<br>k_10_19(33.05,2.89)<br>k_10_30(-10.70,-2.86)<br>k_5_26(-7.50,-2.36)<br>k_2_6(2.67,2.28)<br>k_1_19(-14.00,-2.17)	Intercept(-1.79,-2.10)<br>k_10_19(18.15,4.65)<br>k_10_30(-6.36,-3.42)<br>k_1_3(-6.41,-2.48)<br>k_1_26(-6.95,-3.49)	TRN:(100,89,72)<br>VAL:(100,90,90)<br>FT:(100,94,65)	TRN:(81,96,100<br>VAL:(67,100,10<br>FT:(74,91,100)
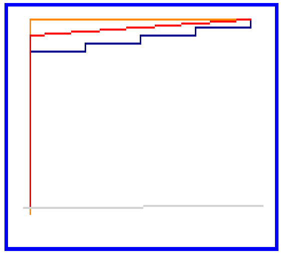	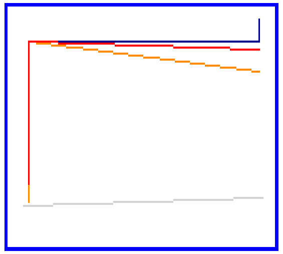	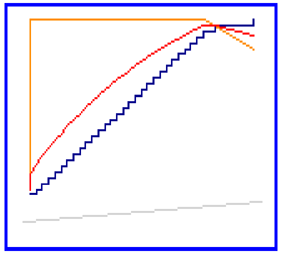	19. Menstruation<br>(26,9,35)<br>k_SBC_30<br>Ginni_TRN: 92<br>Ginni_VAL: 90<br>Ginni__FT: 95	Intercept(23.26,0.00)<br>k_30_19(487.75,.)<br>k_5_26(-219.25,-0.02)<br>k_5_31(-92.78,-0.12)<br>k_5_33(96.90,0.01)	Intercept(-2.56,-1.72)<br>k_30_19(47.69,1.60)<br>k_20_3(-13.27,-1.33)<br>k_20_10(13.18,1.50)<br>k_5_26(-21.08,-1.36)<br>k_1_31(-11.29,-1.65)	TRN:(100,100,100)<br>VAL:(89,89,75)<br>FT:(100,97,85)	TRN:(85,100,10<br>VAL:(89,89,100<br>FT:(74,97,100)
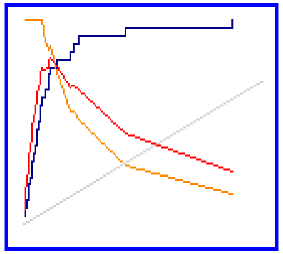	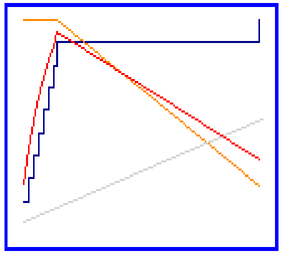	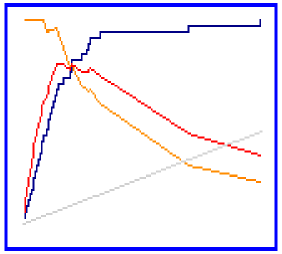	19. Menstruation<br>(26,9,35)<br>k_XERROR_1<br>Ginni_TRN: 91<br>Ginni_VAL: 90<br>Ginni__FT: 93	Intercept(-1.23,-1.75)<br>k_1_3(-4.03,-2.27)<br>k_1_6(1.15,2.00)<br>k_1_19(15.61,4.76)<br>k_1_26(-5.92,-2.99)<br>k_1_37(-3.21,-3.62)	Intercept(-2.61,-3.64)<br>k_1_3(-5.47,-2.78)<br>k_1_6(1.16,2.17)<br>k_1_11(-4.63,-2.08)<br>k_1_19(15.17,5.85)<br>k_1_26(-3.79,-3.10)<br>k_1_37(-1.78,-2.51)	TRN:(100,87,51)<br>VAL:(100,100,100)<br>FT:(100,78,59)	TRN:(58,77,92)<br>VAL:(78,89,89)<br>FT:(37,80,94)

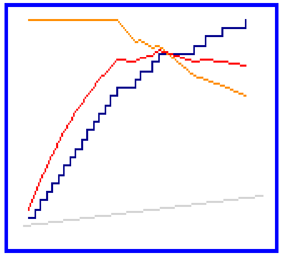	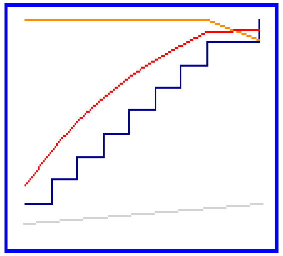	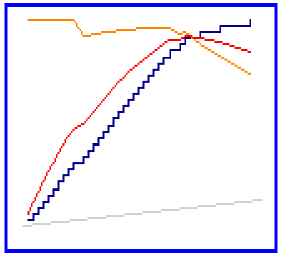	20. Naturheilkunde<br>(24,9,33)<br>k_SBC_10<br>Ginni_TRN: 95<br>Ginni_VAL: 94<br>Ginni__FT: 95	Intercept(-4.22,-4.86)<br>k_10_20(8.94,4.69)<br>k_2_35(-2.93,-2.11)	Intercept(-6.00,-5.26)<br>k_10_16(-5.03,-1.99)<br>k_10_20(10.42,5.12)<br>k_5_9(-3.41,-2.78)	TRN:(100,87,63)<br>VAL:(100,90,90)<br>FT:(100,94,73)	TRN:(54,83,100<br>VAL:(89,100,10<br>FT:(30,91,100)
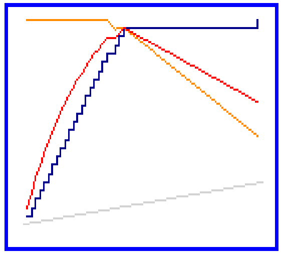	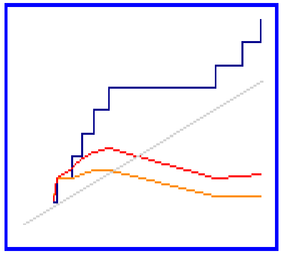	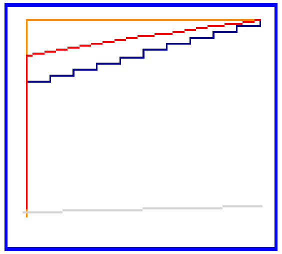	20. Naturheilkunde<br>(24,9,33)<br>svd_XMISC_40<br>Ginni_TRN: 95<br>Ginni_VAL: 68<br>Ginni__FT: 93	Intercept(-21.24,-3.38)<br>s5(34.30,3.14)<br>s10(16.65,2.23)<br>s15(-55.59,-2.95)<br>s23(-69.17,-3.31)<br>s27(-87.62,-2.85)<br>s39(70.66,3.30)<br>s54(84.85,2.99)<br>s90(90.70,2.87)<br>s186(48.65,2.78)<br>s188(83.90,2.95)<br>s191(81.00,3.05)<br>s471(30.41,2.87)	Intercept(-1527.7,-0.64)<br>s5(3775.22,0.06)<br>s8(2809.31,0.17)<br>s23(-4744.5,-0.21)<br>s27(-4083.9,.)<br>s41(2506.47,0.05)<br>s52(4110.50,0.19)<br>s107(-4491.9,-0.05)<br>s126(-3465.9,.)<br>s144(4116.73,.)<br>s157(-3037.0,-0.03)<br>s163(1708.20,.)<br>s188(5367.02,0.05)<br>s196(9625.33,.)<br>s216(3518.93,0.05)<br>s305(-1870.3,.)<br>s441(1942.10,0.18)<br>s449(1612.61,.)	TRN:(100,96,96)<br>VAL:(26,26,26)<br>FT:(100,100,100)	TRN:(54,96,96)<br>VAL:(56,67,67)<br>FT:(100,100,10
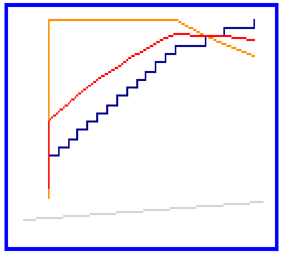	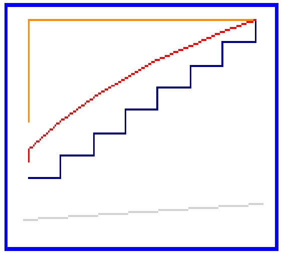	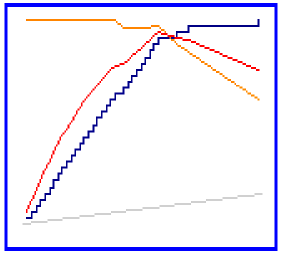	20. Naturheilkunde<br>(24,9,33)<br>k_XERROR_40<br>Ginni_TRN: 95<br>Ginni_VAL: 94<br>Ginni__FT: 95	Intercept(-8.05,-1.93)<br>k_40_20(32.27,1.54)<br>k_40_27(9.30,1.39)<br>k_20_35(-44.27,-1.38)<br>k_10_35(38.53,1.39)	Intercept(-5.64,-5.75)<br>k_40_20(8.33,5.01)<br>k_1_9(-3.87,-2.00)	TRN:(100,100,83)<br>VAL:(100,100,100)<br>FT:(100,97,84)	TRN:(88,88,100<br>VAL:(100,100,1<br>FT:(64,91,97)

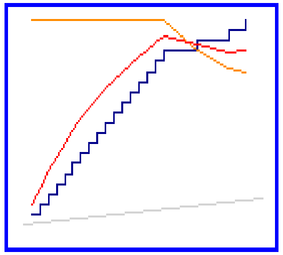	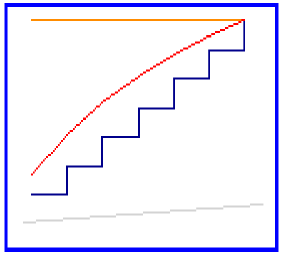	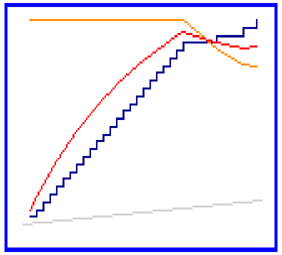	21. Pco<br>(20,7,27)<br>k_XERROR_1<br>Ginni_TRN: 95<br>Ginni_VAL: 95<br>Ginni__FT: 95	Intercept(-6.30,-5.04)<br>k_1_21(3.74,4.72)	Intercept(-6.81,-5.17)<br>k_1_21(4.04,5.05)	TRN:(100,100,74)<br>VAL:(100,100,100)<br>FT:(100,100,77)	TRN:(70,85,100<br>VAL:(100,100,1<br>FT:(89,89,100)
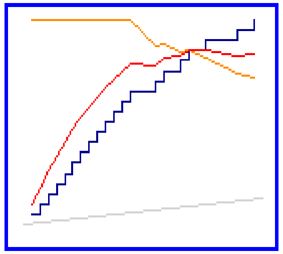	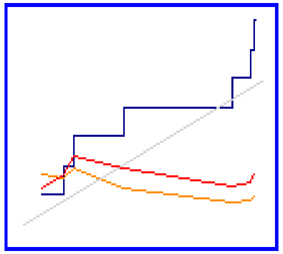	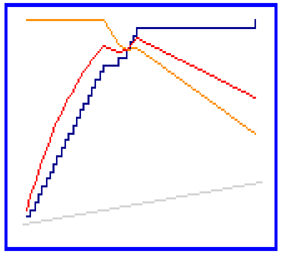	21. Pco<br>(20,7,27)<br>svd_XERROR_40<br>Ginni_TRN: 95<br>Ginni_VAL: 63<br>Ginni__FT: 95	Intercept(-15.48,-3.17)<br>s19(-65.39,-2.94)<br>s68(71.59,3.00)<br>s103(36.43,2.64)<br>s147(46.25,2.45)<br>s166(93.72,3.09)<br>s199(-34.88,-2.55)<br>s282(-19.92,-2.83)<br>s431(-39.69,-2.64)	Intercept(-15.60,-3.26)<br>s11(32.30,2.60)<br>s19(-48.97,-3.38)<br>s68(42.66,2.87)<br>s102(-61.02,-2.72)<br>s199(-44.97,-2.71)<br>s282(-25.34,-2.95)<br>s344(-45.25,-2.84)<br>s380(-46.53,-2.83)<br>s412(-15.61,-2.59)<br>s447(-15.47,-2.41)<br>s452(30.39,2.94)	TRN:(100,82,71)<br>VAL:(27,27,14)<br>FT:(100,87,87)	TRN:(35,90,100<br>VAL:(43,43,100<br>FT:(48,96,96)
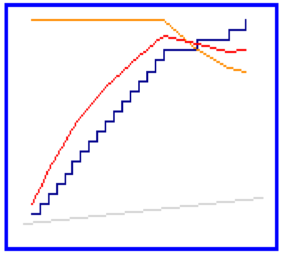	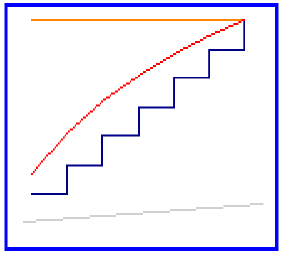	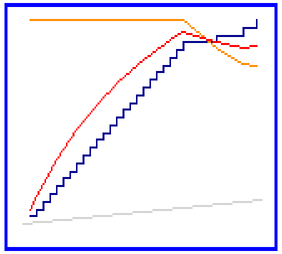	21. Pco<br>(20,7,27)<br>k_XERROR_1<br>Ginni_TRN: 95<br>Ginni_VAL: 95<br>Ginni__FT: 95	Intercept(-6.30,-5.04)<br>k_1_21(3.74,4.72)	Intercept(-6.81,-5.17)<br>k_1_21(4.04,5.05)	TRN:(100,100,74)<br>VAL:(100,100,100)<br>FT:(100,100,77)	TRN:(70,85,100<br>VAL:(100,100,1<br>FT:(89,89,100)

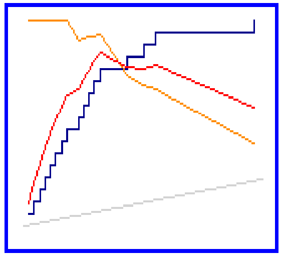	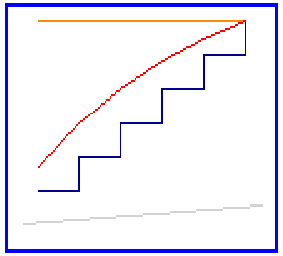	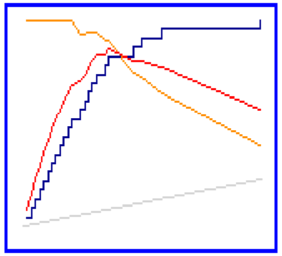	22. Pille<br>(17,6,23)<br>k_XMISC_1<br>Ginni_TRN: 94<br>Ginni_VAL: 95<br>Ginni__FT: 94	Intercept(-3.57,-3.54)<br>k_1_22(6.48,4.39)<br>k_1_37(-3.48,-2.55)	Intercept(-3.48,-3.65)<br>k_1_22(7.03,4.88)<br>k_1_37(-4.31,-3.03)	TRN:(100,93,67)<br>VAL:(100,100,100)<br>FT:(100,90,65)	TRN:(47,76,94)<br>VAL:(100,100,1<br>FT:(43,83,96)
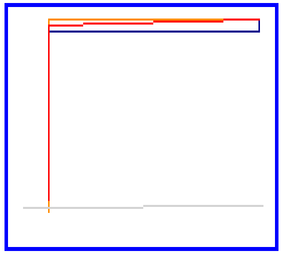	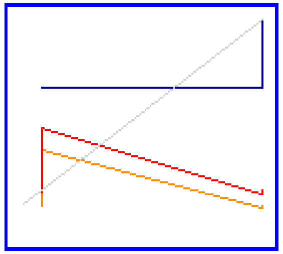	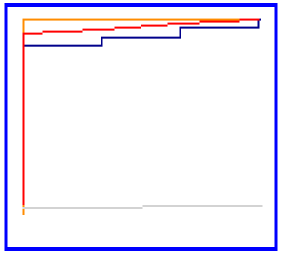	22. Pille<br>(17,6,23)<br>svd_XMISC_40<br>Ginni_TRN: 92<br>Ginni_VAL: 55<br>Ginni__FT: 92	Intercept(-4896.3,-178.59)<br>s5(17325.2,7.53)<br>s8(14848.4,.)<br>s17(-21044,.)<br>s199(8237.46,.)<br>s399(-8647.3,.)<br>s474(11452.7,21.42)	Intercept(-7290.7,-98.70)<br>s5(19409.0,.)<br>s8(21026.0,.)<br>s17(-34573,.)<br>s51(20313.9,33.76)<br>s207(-13142,-16.35)<br>s297(-8953.3,-4.36)<br>s334(-7016.6,.)<br>s388(7998.22,6.36)<br>s429(11691.1,10.47)	TRN:(100,100,100)<br>VAL:(36,36,36)<br>FT:(100,100,100)	TRN:(100,100,1<br>VAL:(67,67,67)<br>FT:(100,100,10
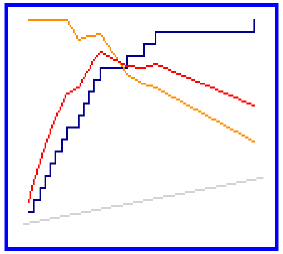	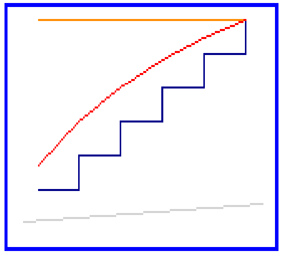	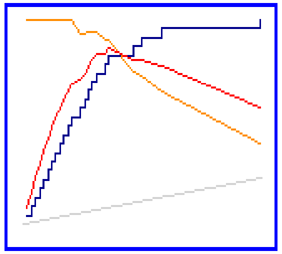	22. Pille<br>(17,6,23)<br>k_XMISC_1<br>Ginni_TRN: 94<br>Ginni_VAL: 95<br>Ginni__FT: 94	Intercept(-3.57,-3.54)<br>k_1_22(6.48,4.39)<br>k_1_37(-3.48,-2.55)	Intercept(-3.48,-3.65)<br>k_1_22(7.03,4.88)<br>k_1_37(-4.31,-3.03)	TRN:(100,93,67)<br>VAL:(100,100,100)<br>FT:(100,90,65)	TRN:(47,76,94)<br>VAL:(100,100,1<br>FT:(43,83,96)

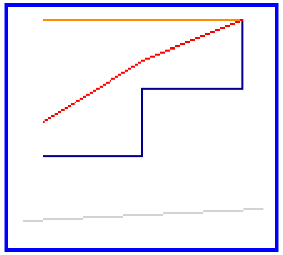	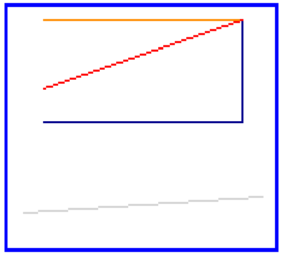	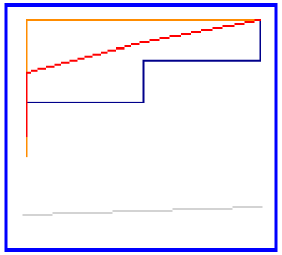	23.<br>Probleme_geschlechtsverk.<br>(3,2,5)<br>k_XMISC_10<br>Ginni_TRN: 95<br>Ginni_VAL: 90<br>Ginni__FT: 93	Intercept(-16.89,-0.05)<br>k_10_23(14.31,0.07)	Intercept(-36.98,-0.01)<br>k_10_23(30.52,0.00)	TRN:(100,100,100)<br>VAL:(100,100,100)<br>FT:(100,100,100)	TRN:(67,100,10<br>VAL:(100,100,1<br>FT:(100,100,10
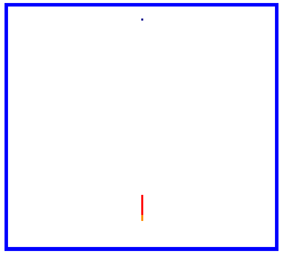	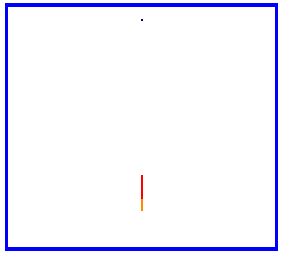	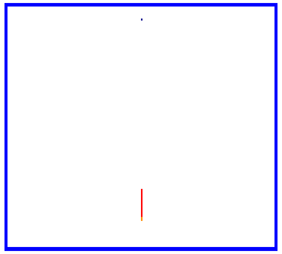	23.<br>Probleme_geschlechtsverk.<br>(3,2,5)<br>k_XERROR_1<br>Ginni_TRN: 0<br>Ginni_VAL: 0<br>Ginni__FT: 0	Intercept(-2.51,-4.19)	Intercept(-2.30,-4.91)	TRN:(8,8,8)<br>VAL:(13,13,13)<br>FT:(9,9,9)	TRN:(100,100,1<br>VAL:(100,100,1<br>FT:(100,100,10
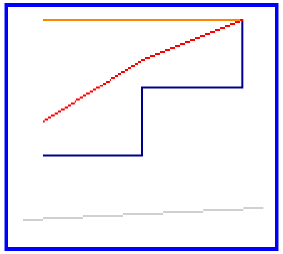	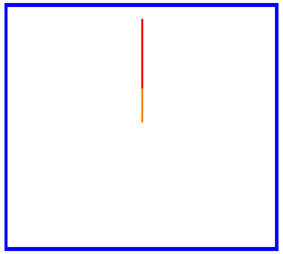	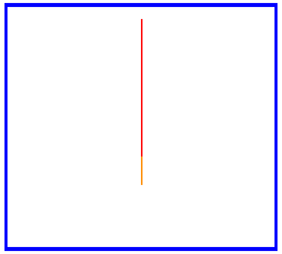	23.<br>Probleme_geschlechtsverk.<br>(3,2,5)<br>k_AIC_1<br>Ginni_TRN: 95<br>Ginni_VAL: 87<br>Ginni__FT: 91	Intercept(-17.48,-0.07)<br>k_1_23(17.45,0.09)	Intercept(-128.05,-3.75)<br>k_1_23(117.14,.)	TRN:(100,100,100)<br>VAL:(100,100,100)<br>FT:(100,100,100)	TRN:(67,100,10<br>VAL:(100,100,1<br>FT:(100,100,10

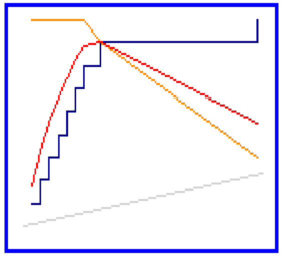	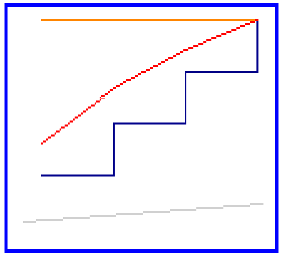	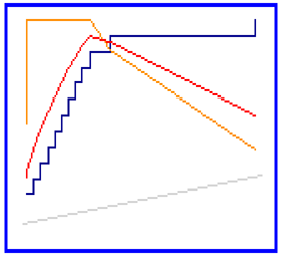	24. Schilddrüse<br>(9,4,13)<br>k_XERROR_1<br>Ginni_TRN: 93<br>Ginni_VAL: 93<br>Ginni__FT: 94	Intercept(-6.36,-3.84)<br>k_1_24(4.17,2.73)	Intercept(-5.99,-4.83)<br>k_1_24(3.31,3.56)	TRN:(100,89,89)<br>VAL:(100,100,100)<br>FT:(100,100,86)	TRN:(78,89,89)<br>VAL:(100,100,1<br>FT:(85,85,92)
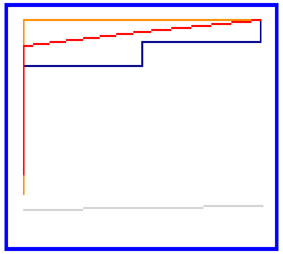	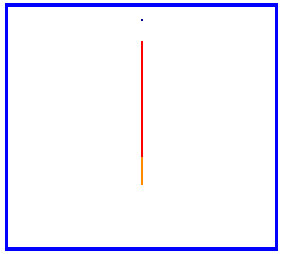	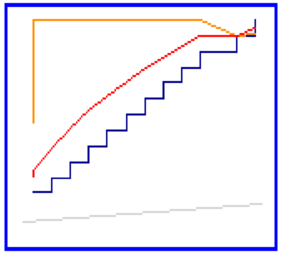	24. Schilddrüse<br>(9,4,13)<br>k_SBC_20<br>Ginni_TRN: 93<br>Ginni_VAL: 86<br>Ginni__FT: 95	Intercept(-19.87,-0.01)<br>k_20_24(84.16,0.01)<br>k_5_37(-64.54,-0.00)	Intercept(-2.47,-1.59)<br>k_20_24(4.97,2.70)<br>k_5_37(-4.23,-1.95)	TRN:(100,100,100)<br>VAL:(80,80,80)<br>FT:(100,93,93)	TRN:(100,100,1<br>VAL:(100,100,1<br>FT:(85,100,100
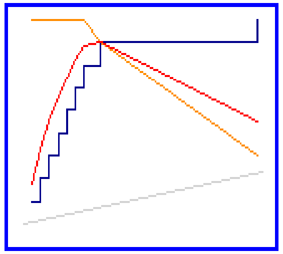	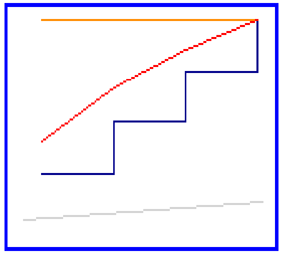	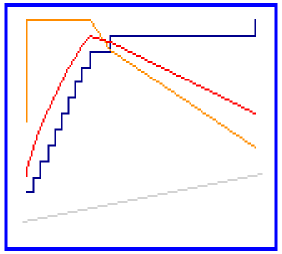	24. Schilddrüse<br>(9,4,13)<br>k_XERROR_1<br>Ginni_TRN: 93<br>Ginni_VAL: 93<br>Ginni__FT: 94	Intercept(-6.36,-3.84)<br>k_1_24(4.17,2.73)	Intercept(-5.99,-4.83)<br>k_1_24(3.31,3.56)	TRN:(100,89,89)<br>VAL:(100,100,100)<br>FT:(100,100,86)	TRN:(78,89,89)<br>VAL:(100,100,1<br>FT:(85,85,92)

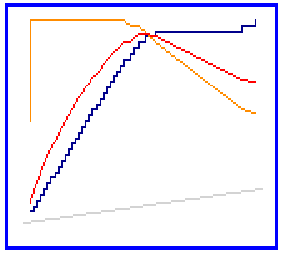	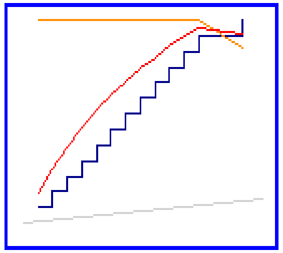	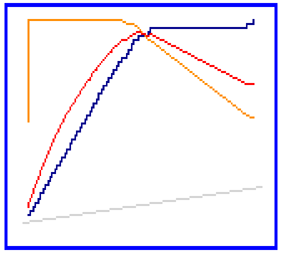	25.<br>Schwangerschaftssorgen<br>(36,13,49)<br>k_SBC_40<br>Ginni_TRN: 95<br>Ginni_VAL: 95<br>Ginni__FT: 95	Intercept(-5.10,-4.97)<br>k_40_25(8.13,4.84)<br>k_20_1(-1.98,-2.27)	Intercept(-5.70,-5.62)<br>k_40_25(8.81,5.42)<br>k_1_1(-2.58,-3.10)	TRN:(100,94,89)<br>VAL:(100,100,87)<br>FT:(100,96,90)	TRN:(75,92,94)<br>VAL:(92,92,100<br>FT:(82,92,96)
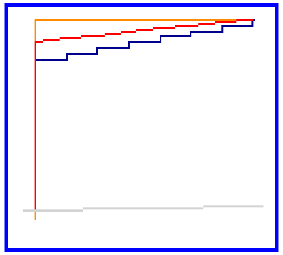	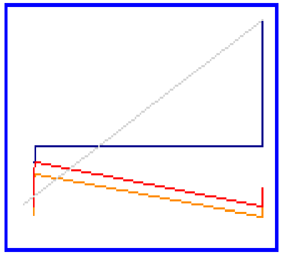	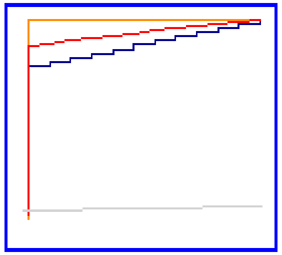	25.<br>Schwangerschaftssorgen<br>(36,13,49)<br>svd_AIC_40<br>Ginni_TRN: 93<br>Ginni_VAL: 33<br>Ginni__FT: 93	Intercept(-8005.7,-1.34)<br>s3(-12650,-1.48)<br>s9(26396.6,.)<br>s21(-11669,.)<br>s56(-16685,.)<br>s67(33051.4,0.34)<br>s78(18305.5,.)<br>s88(-23359,-2.14)<br>s106(22450.7,0.61)<br>s111(10459.8,.)<br>s134(15517.5,.)<br>s142(22563.1,0.71)<br>s278(-6937.3,-0.64)<br>s308(5209.30,0.11)<br>s311(-7267.0,.)<br>s323(-6992.2,-0.20)<br>s362(-12521,-0.99)<br>s412(-12181,-0.30)<br>s437(13462.6,1.03)<br>s490(19125.6,1.00)	Intercept(-717.64,-0.27)<br>s3(-868.94,-0.17)<br>s9(2079.10,0.09)<br>s26(2437.30,0.08)<br>s40(-1024.3,.)<br>s56(-1981.8,-0.11)<br>s67(1915.07,0.13)<br>s78(1338.72,0.06)<br>s118(1637.80,0.06)<br>s137(-1865.8,.)<br>s142(2790.42,0.09)<br>s144(2144.02,0.13)<br>s170(1418.37,0.09)<br>s182(-2128.3,-0.08)<br>s192(1141.93,.)<br>s207(2678.95,0.45)<br>s216(-1414.5,-0.15)<br>s217(1580.81,.)<br>s302(-496.34,-0.10)<br>s308(913.21,.)<br>s358(-475.48,.)<br>s393(602.67,0.07)<br>s454(665.91,0.07)<br>s474(755.38,0.12)<br>s483(1212.43,0.13)<br>s490(1091.48,0.08)	TRN:(100,100,100)<br>VAL:(25,25,25)<br>FT:(100,100,100)	TRN:(83,100,10<br>VAL:(38,38,38)<br>FT:(100,100,10
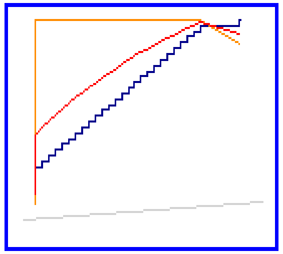	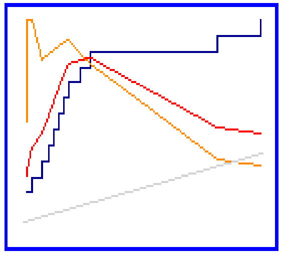	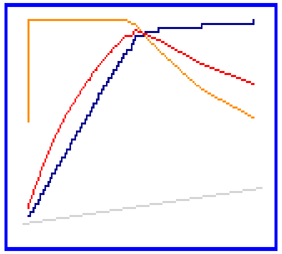	25.<br>Schwangerschaftssorgen<br>(36,13,49)<br>pc_XMISC_40<br>Ginni_TRN: 95<br>Ginni_VAL: 91<br>Ginni__FT: 95	Intercept(-27.10,-2.23)<br>pC_6_5(83.64,2.12)<br>pC_8_1(-0.90,-0.82)<br>pC_18_7(38.33,1.94)<br>pC_25_1(6.82,2.10)<br>pC_25_2(-18.64,-2.03)<br>pC_38_3(-37.36,-1.85)	Intercept(-10.05,-5.12)<br>pC_1_1(-0.77,-2.75)<br>pC_20_7(14.92,2.53)<br>pC_25_1(2.56,4.63)<br>pC_25_2(-2.58,-2.46)	TRN:(100,100,88)<br>VAL:(100,79,79)<br>FT:(100,98,85)	TRN:(83,97,100<br>VAL:(23,85,85)<br>FT:(86,92,96)

file:///E:/TM/himmel/neu_06_06/Report/modelle_best_ginni_precision_recall.html

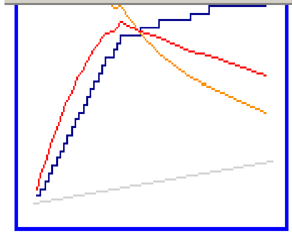	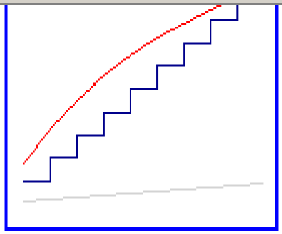	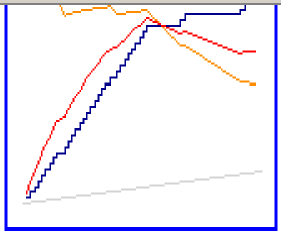	Schwangerschaftssymptome (27,9,36)<br>k_XMISC_20<br>Ginni_TRN: 94<br>Ginni_VAL: 95<br>Ginni__FT: 94	k_20_26(16.20,3.81)<br>k_10_19(-2.85,-2.84)<br>k_2_26(-12.56,-3.16)	k_20_26(13.52,4.48)<br>k_2_26(-10.59,-3.62)	VAL:(100,100,100)<br>FT:(100,94,58)	VAL:(100,100,1<br>FT:(25,86,100)
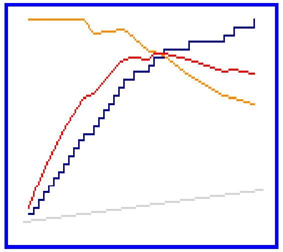	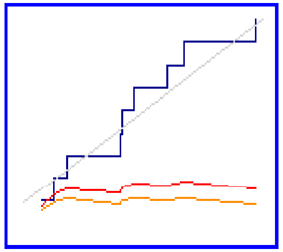	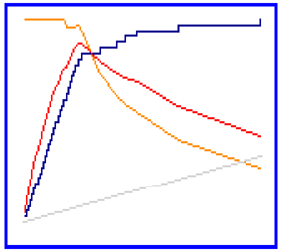	26.<br>Schwangerschaftssymptome (27,9,36)<br>svd_XERROR_40<br>Ginni_TRN: 94<br>Ginni_VAL: 51<br>Ginni__FT: 94	Intercept(-11.33,-4.14)<br>s2(46.28,3.76)<br>s64(-39.94,-3.41)<br>s127(-32.17,-3.18)<br>s138(-20.82,-2.67)<br>s181(43.01,3.38)<br>s193(39.39,2.73)<br>s306(19.91,3.45)<br>s401(20.41,3.29)<br>s428(22.74,3.69)<br>s439(24.32,3.44)	Intercept(-10.31,-5.71)<br>s2(26.71,4.03)<br>s9(11.15,2.23)<br>s11(-28.24,-4.28)<br>s14(-39.10,-4.17)<br>s19(14.17,2.75)<br>s43(18.22,2.69)<br>s52(-36.90,-4.21)<br>s145(-18.32,-2.65)<br>s195(23.52,2.78)<br>s251(14.53,4.27)<br>s320(-10.39,-3.25)<br>s334(-10.42,-3.23)<br>s360(15.88,4.25)<br>s401(17.19,3.94)<br>s421(-9.86,-2.94)<br>s440(23.79,4.70)	TRN:(100,82,59)<br>VAL:(12,11,11)<br>FT:(100,94,55)	TRN:(44,85,100<br>VAL:(67,89,89)<br>FT:(61,83,94)
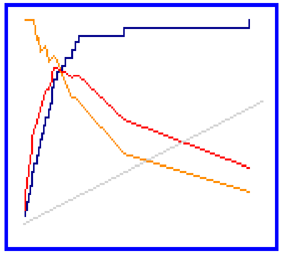	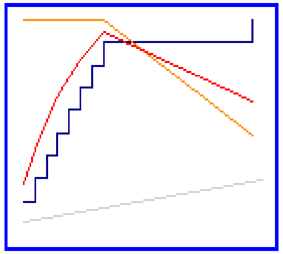	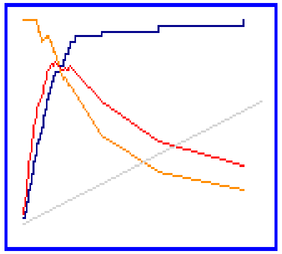	26.<br>Schwangerschaftssymptome (27,9,36)<br>k_XMISC_5<br>Ginni_TRN: 92<br>Ginni_VAL: 94<br>Ginni__FT: 92	Intercept(-4.76,-8.39)<br>k_5_26(3.72,6.79)	Intercept(-5.41,-8.47)<br>k_5_26(11.92,4.04)<br>k_2_26(-8.20,-2.82)	TRN:(100,80,60)<br>VAL:(100,100,100)<br>FT:(100,84,63)	TRN:(22,74,93)<br>VAL:(89,89,89)<br>FT:(33,75,92)

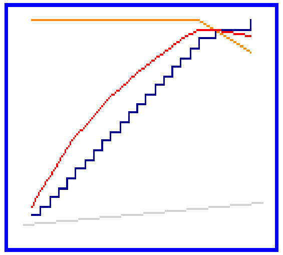	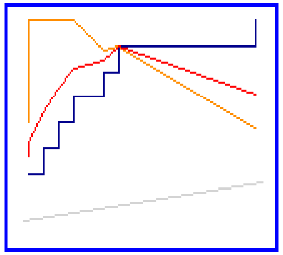	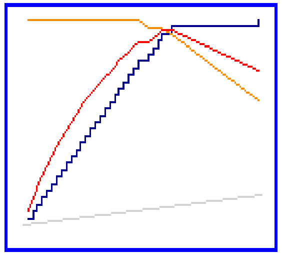	27. Schwangerschaftstest<br>(22,8,30)<br>k_XERROR_20<br>Ginni_TRN: 95<br>Ginni_VAL: 93<br>Ginni__FT: 95	Intercept(-13.13,-2.34)<br>k_20_27(27.44,2.26)<br>k_5_19(-15.58,-2.02)	Intercept(-4.64,-2.97)<br>k_20_26(-2.80,-2.59)<br>k_20_27(17.23,3.87)<br>k_20_36(-2.94,-1.87)	TRN:(100,95,85)<br>VAL:(100,88,47)<br>FT:(100,94,94)	TRN:(91,95,100<br>VAL:(63,88,100<br>FT:(70,97,97)
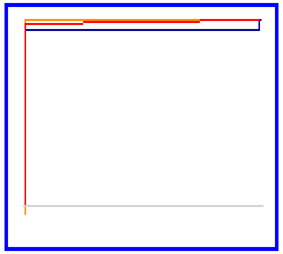	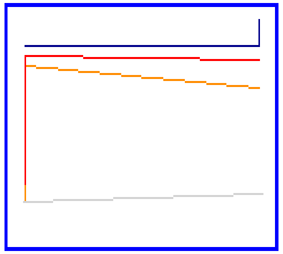	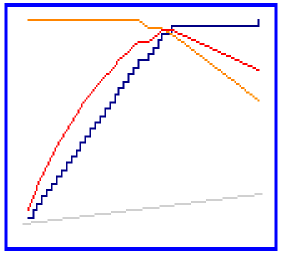	27. Schwangerschaftstest<br>(22,8,30)<br>k_SBC_40<br>Ginni_TRN: 91<br>Ginni_VAL: 89<br>Ginni__FT: 95	Intercept(-2070.5,-39.61)<br>k_40_31(291.66,1.93)<br>k_20_27(4192.19,.)<br>k_5_19(-2293.9,.)	Intercept(-4.78,-3.09)<br>k_40_36(-2.65,-1.85)<br>k_20_26(-2.73,-2.47)<br>k_20_27(17.23,3.86)	TRN:(100,100,100)<br>VAL:(78,78,67)<br>FT:(100,94,94)	TRN:(95,100,10<br>VAL:(88,88,100<br>FT:(70,97,97)
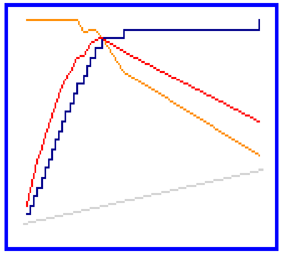	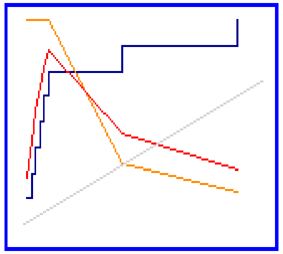	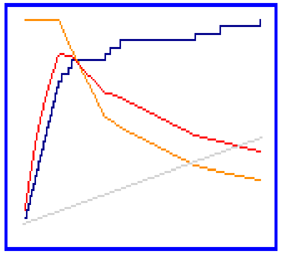	27. Schwangerschaftstest<br>(22,8,30)<br>k_SBC_2<br>Ginni_TRN: 94<br>Ginni_VAL: 85<br>Ginni__FT: 92	Intercept(-4.43,-3.03)<br>k_2_8(-12.11,-2.02)<br>k_2_27(13.58,4.43)<br>k_1_33(3.69,2.47)	Intercept(-3.30,-4.68)<br>k_2_37(-1.23,-2.53)<br>k_1_27(10.94,5.97)	TRN:(100,91,75)<br>VAL:(100,100,100)<br>FT:(100,96,47)	TRN:(64,91,95)<br>VAL:(63,75,75)<br>FT:(70,73,90)

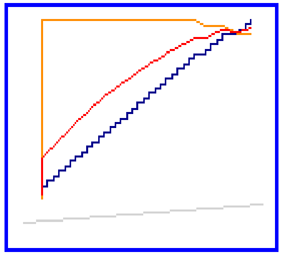	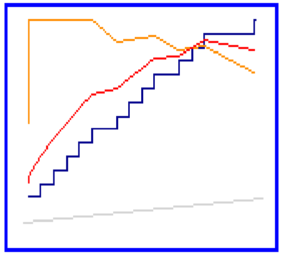	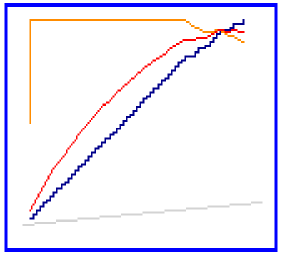	28. Spermiogramm<br>(42,15,57)<br>pc_XERROR_40<br>Ginni_TRN: 95<br>Ginni_VAL: 94<br>Ginni__FT: 95	Intercept(-22.07,-2.60)<br>pC_5_3(41.10,2.44)<br>pC_28_1(5.39,2.61)<br>pC_28_2(-12.31,-1.99)<br>pC_33_1(2.81,2.48)	Intercept(-13.82,-4.56)<br>pC_2_7(-23.41,-2.27)<br>pC_3_4(10.32,2.56)<br>pC_5_3(16.41,2.66)<br>pC_19_2(-3.98,-2.49)<br>pC_28_1(3.52,4.61)<br>pC_33_1(1.20,3.00)	TRN:(100,93,93)<br>VAL:(100,88,75)<br>FT:(100,92,89)	TRN:(62,100,10<br>VAL:(47,93,100<br>FT:(81,98,100)
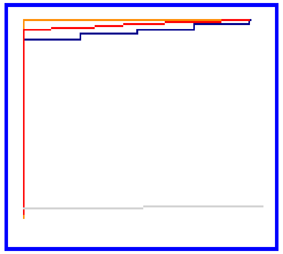	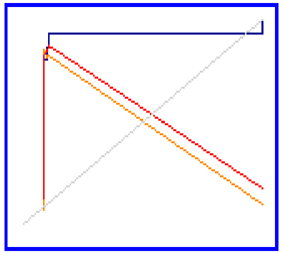	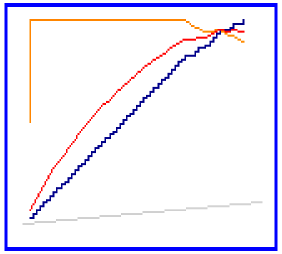	28. Spermiogramm<br>(42,15,57)<br>pc_XMISC_40<br>Ginni_TRN: 92<br>Ginni_VAL: 85<br>Ginni__FT: 95	Intercept(-2080.5,-3.19)<br>pC_5_3(3927.66,2.40)<br>pC_6_4(-3307.6,-3.21)<br>pC_28_1(492.74,.)<br>pC_28_2(-642.90,-1.46)<br>pC_33_1(208.65,0.86)	Intercept(-13.82,-4.56)<br>pC_2_7(-23.41,-2.27)<br>pC_3_4(10.32,2.56)<br>pC_5_3(16.41,2.66)<br>pC_19_2(-3.98,-2.49)<br>pC_28_1(3.52,4.61)<br>pC_33_1(1.20,3.00)	TRN:(100,100,100)<br>VAL:(86,82,82)<br>FT:(100,92,89)	TRN:(100,100,1<br>VAL:(80,93,93)<br>FT:(81,98,100)
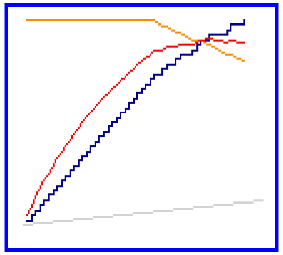	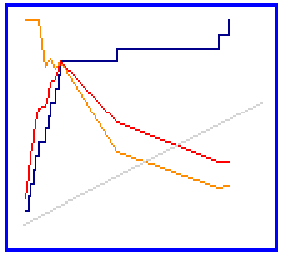	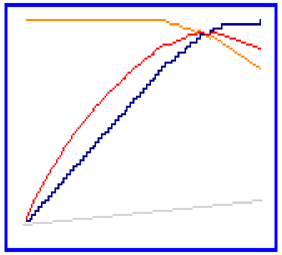	28. Spermiogramm<br>(42,15,57)<br>svd_XERROR_40<br>Ginni_TRN: 95<br>Ginni_VAL: 88<br>Ginni__FT: 95	Intercept(-13.23,-4.15)<br>s2(-36.15,-3.94)<br>s4(75.47,3.93)<br>s19(24.97,2.50)<br>s25(44.91,3.11)<br>s32(-27.09,-2.88)<br>s114(-28.98,-3.17)<br>s164(35.41,2.62)	Intercept(-0.03,-0.02)<br>s1(-80.14,-3.85)<br>s2(-51.22,-3.48)<br>s4(95.32,3.77)<br>s19(46.46,3.08)<br>s71(-50.75,-3.32)<br>s83(-27.31,-2.70)<br>s105(48.94,3.53)<br>s218(-39.66,-2.85)<br>s241(13.97,2.97)<br>s278(15.33,2.66)	TRN:(100,89,81)<br>VAL:(100,80,80)<br>FT:(100,92,89)	TRN:(62,93,100<br>VAL:(40,80,80)<br>FT:(79,96,98)

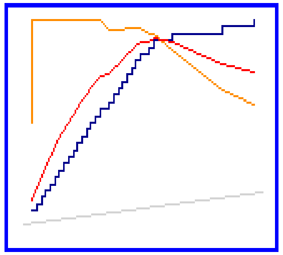	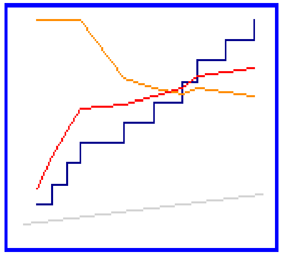	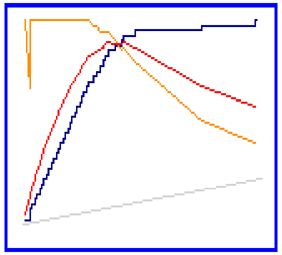	29. Stimulation<br>(30,10,40)<br>k_XERROR_10<br>Ginni_TRN: 95<br>Ginni_VAL: 93<br>Ginni__FT: 95	Intercept(-4.47,-4.74)<br>k_10_16(-6.29,-3.26)<br>k_10_29(20.05,3.88)<br>k_1_29(-9.63,-2.87)<br>k_1_35(-4.97,-2.95)	Intercept(-4.45,-4.77)<br>k_10_10(-7.85,-2.25)<br>k_10_13(-4.48,-2.15)<br>k_10_16(-6.17,-3.21)<br>k_10_29(19.77,4.70)<br>k_2_34(-4.14,-3.15)<br>k_1_29(-10.01,-3.30)	TRN:(100,93,59)<br>VAL:(100,63,63)<br>FT:(100,94,79)	TRN:(43,90,100<br>VAL:(40,100,10<br>FT:(70,85,95)
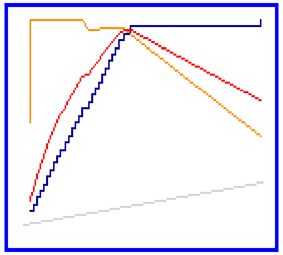	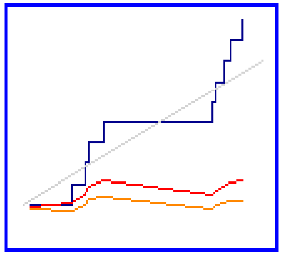	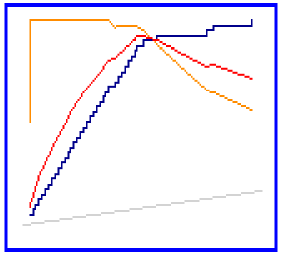	29. Stimulation<br>(30,10,40)<br>svd_XMISC_40<br>Ginni_TRN: 95<br>Ginni_VAL: 53<br>Ginni__FT: 95	Intercept(-12.43,-3.94)<br>s4(-70.13,-3.55)<br>s9(-50.59,-3.36)<br>s20(22.87,2.53)<br>s43(-34.12,-3.15)<br>s151(-49.52,-3.36)<br>s198(-36.07,-2.88)<br>s309(-22.03,-3.22)<br>s323(-15.94,-3.00)<br>s346(16.76,3.04)<br>s408(17.39,2.56)<br>s427(18.91,2.71)<br>s459(17.26,2.76)	Intercept(-19.57,-3.25)<br>s4(-72.52,-3.00)<br>s9(-90.06,-3.05)<br>s13(25.04,2.76)<br>s43(-47.44,-2.98)<br>s47(61.57,2.71)<br>s128(32.59,2.80)<br>s149(-48.63,-2.81)<br>s151(-39.84,-3.13)<br>s193(66.39,3.06)<br>s202(-59.47,-2.74)<br>s203(92.30,2.82)<br>s225(-79.83,-3.02)<br>s226(-46.53,-2.79)<br>s227(94.48,3.20)<br>s346(21.59,2.82)<br>s364(31.70,2.95)<br>s385(-12.03,-2.32)<br>s466(28.54,3.07)<br>s492(39.46,3.25)	TRN:(100,94,94)<br>VAL:(14,12,12)<br>FT:(100,95,65)	TRN:(43,97,97)<br>VAL:(50,100,10<br>FT:(55,90,98)
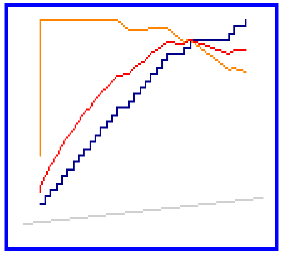	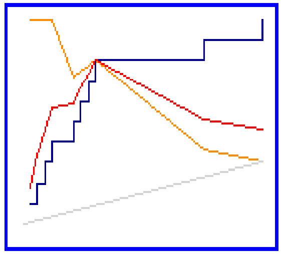	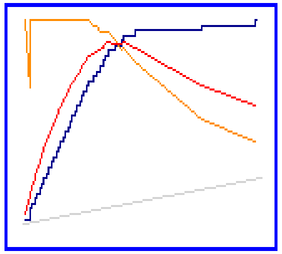	29. Stimulation<br>(30,10,40)<br>k_XMISC_10<br>Ginni_TRN: 95<br>Ginni_VAL: 91<br>Ginni__FT: 95	Intercept(-3.92,-4.07)<br>k_10_16(-5.81,-2.84)<br>k_10_29(23.30,3.87)<br>k_2_18(-10.21,-1.98)<br>k_1_29(-11.63,-2.88)<br>k_1_35(-5.13,-2.95)	Intercept(-4.45,-4.77)<br>k_10_10(-7.85,-2.25)<br>k_10_13(-4.48,-2.15)<br>k_10_16(-6.17,-3.21)<br>k_10_29(19.77,4.70)<br>k_2_34(-4.14,-3.15)<br>k_1_29(-10.01,-3.30)	TRN:(100,90,75)<br>VAL:(100,80,80)<br>FT:(100,94,79)	TRN:(43,90,100<br>VAL:(40,80,80)<br>FT:(70,85,95)

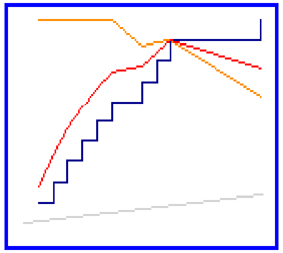	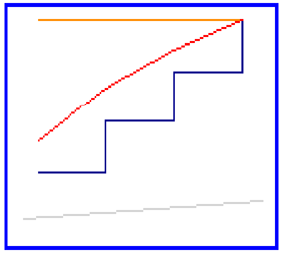	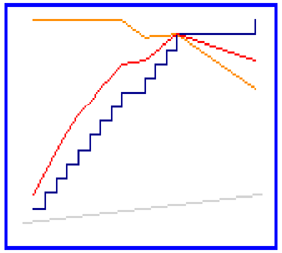	30. Zwischenblutung<br>(10,4,14)<br>k_XERROR_1<br>Ginni_TRN: 94<br>Ginni_VAL: 94<br>Ginni__FT: 95	Intercept(-7.72,-3.44)<br>k_1_30(5.45,3.12)	Intercept(-8.72,-3.60)<br>k_1_30(6.19,3.33)	TRN:(100,90,63)<br>VAL:(100,100,100)<br>FT:(100,93,67)	TRN:(50,90,100<br>VAL:(100,100,1<br>FT:(64,93,100)
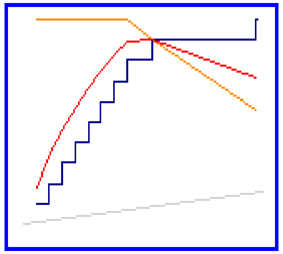	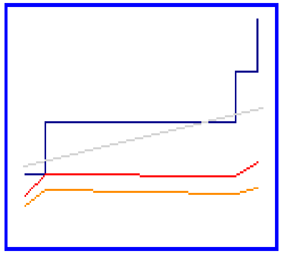	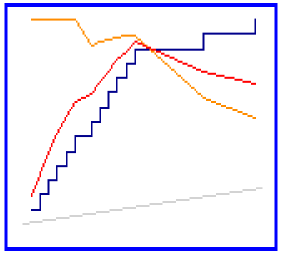	30. Zwischenblutung<br>(10,4,14)<br>svd_XERROR_40<br>Ginni_TRN: 94<br>Ginni_VAL: 58<br>Ginni__FT: 94	Intercept(-35.30,-1.60)<br>s67(-124.04,-1.52)<br>s82(-211.49,-1.58)<br>s237(-64.68,-1.63)<br>s297(133.92,1.59)<br>s423(153.77,1.49)	Intercept(-10.07,-3.49)<br>s11(-52.17,-3.31)<br>s115(-42.12,-2.76)<br>s139(41.90,2.51)<br>s155(47.86,2.80)<br>s339(-11.67,-2.41)<br>s354(-16.01,-2.66)<br>s400(-25.15,-2.86)	TRN:(100,90,56)<br>VAL:(18,18,18)<br>FT:(100,92,52)	TRN:(80,90,100<br>VAL:(100,100,1<br>FT:(43,86,100)
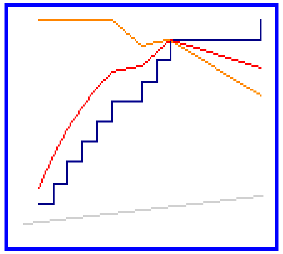	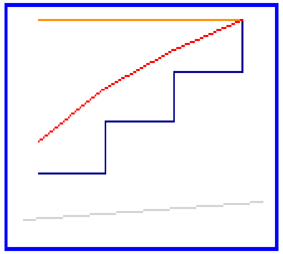	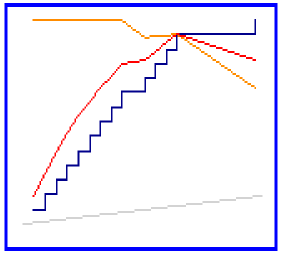	30. Zwischenblutung<br>(10,4,14)<br>k_XERROR_1<br>Ginni_TRN: 94<br>Ginni_VAL: 94<br>Ginni__FT: 95	Intercept(-7.72,-3.44)<br>k_1_30(5.45,3.12)	Intercept(-8.72,-3.60)<br>k_1_30(6.19,3.33)	TRN:(100,90,63)<br>VAL:(100,100,100)<br>FT:(100,93,67)	TRN:(50,90,100<br>VAL:(100,100,1<br>FT:(64,93,100)

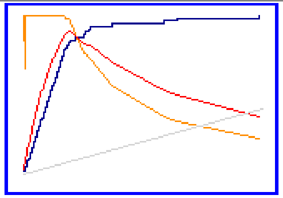	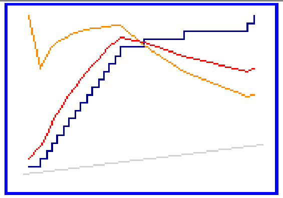	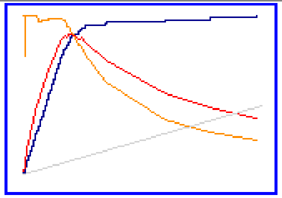	31. Zyklus<br>(59,20,79)<br>k_XERROR_30<br>Ginni_TRN: 94<br>Ginni_VAL: 94<br>Ginni__FT: 94	Intercept(-5.99,-6.41)<br>k_30_14(1.21,2.37)<br>k_30_31(13.20,6.12)<br>k_5_19(-5.09,-3.19)<br>k_5_20(-8.91,-4.07)<br>k_2_28(1.29,3.57)<br>k_1_6(-2.01,-2.98)<br>k_1_31(-7.23,-4.15)<br>k_1_38(-1.51,-2.73)	Intercept(-6.06,-8.35)<br>k_30_13(2.23,3.22)<br>k_30_14(1.46,3.47)<br>k_30_25(1.54,2.81)<br>k_30_31(15.58,7.37)<br>k_10_20(6.77,2.57)<br>k_5_20(-14.15,-3.72)<br>k_2_28(1.28,4.59)<br>k_1_26(-2.97,-3.04)<br>k_1_31(-10.23,-5.85)	TRN:(100,96,72)<br>VAL:(100,94,50)<br>FT:(100,90,76)	TRN:(78,85,93)<br>VAL:(5,80,100)<br>FT:(19,87,94)
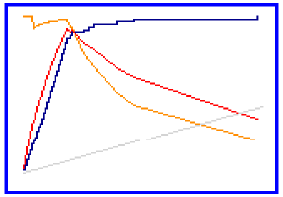	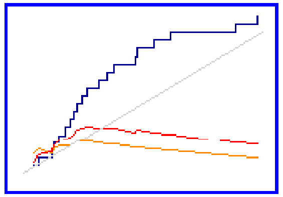	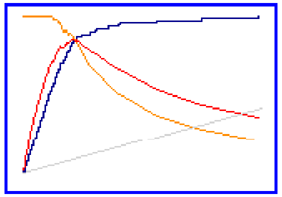	31. Zyklus<br>(59,20,79)<br>svd_XERROR_40<br>Ginni_TRN: 94<br>Ginni_VAL: 69<br>Ginni__FT: 94	Intercept(-11.35,-6.28)<br>s2(24.18,5.20)<br>s4(14.50,2.54)<br>s5(21.62,4.58)<br>s9(-16.34,-2.62)<br>s10(29.04,4.34)<br>s36(35.50,4.21)<br>s51(19.57,3.64)<br>s60(-20.09,-3.27)<br>s65(-15.04,-3.04)<br>s68(21.94,3.52)<br>s73(-22.20,-3.21)<br>s90(-15.89,-2.50)<br>s95(-34.55,-4.39)<br>s101(18.67,3.18)<br>s111(-34.07,-4.21)<br>s135(26.84,4.27)<br>s146(30.55,4.50)<br>s156(-12.31,-2.04)<br>s232(-9.20,-3.31)<br>s235(13.23,3.80)<br>s266(-8.11,-2.93)<br>s284(9.34,2.92)<br>s301(10.92,3.77)<br>s302(8.93,2.99)<br>s304(15.19,3.54)<br>s329(-11.88,-3.63)<br>s337(-8.34,-2.63)<br>s383(16.13,4.38)<br>s420(-9.53,-2.82)<br>s440(-11.75,-3.19)<br>s451(-10.65,-3.29)<br>s478(-17.14,-4.31)<br>s482(-7.95,-2.65)	Intercept(-10.14,-7.69)<br>s2(28.97,6.46)<br>s4(16.97,4.01)<br>s5(20.02,5.65)<br>s10(19.06,5.27)<br>s13(18.67,4.35)<br>s20(26.77,5.61)<br>s47(15.30,3.88)<br>s68(17.17,3.69)<br>s73(-18.99,-4.04)<br>s77(23.56,4.64)<br>s90(-15.15,-3.78)<br>s93(-19.94,-3.93)<br>s101(29.86,5.54)<br>s102(24.17,4.51)<br>s135(8.64,2.14)<br>s137(22.87,4.74)<br>s143(17.20,3.71)<br>s207(17.11,3.62)<br>s223(-21.14,-4.20)<br>s226(27.85,4.94)<br>s235(9.36,4.39)<br>s258(-9.02,-4.33)<br>s282(9.31,3.97)<br>s300(7.92,3.39)<br>s304(5.40,2.35)<br>s315(-4.81,-2.39)<br>s337(-8.29,-3.45)<br>s346(6.23,2.92)<br>s380(5.99,3.04)<br>s383(10.80,4.51)<br>s395(6.99,3.04)<br>s396(-6.84,-3.18)<br>s415(6.68,2.88)<br>s417(-8.88,-3.96)<br>s450(7.85,3.02)<br>s451(-13.11,-4.48)<br>s452(-8.62,-3.28)<br>s478(-9.42,-3.26)<br>s493(-9.99,-3.11)<br>s494(-8.59,-3.45)	TRN:(100,98,68)<br>VAL:(21,21,17)<br>FT:(100,88,65)	TRN:(19,86,95)<br>VAL:(50,55,80)<br>FT:(38,84,92)
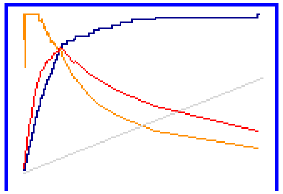	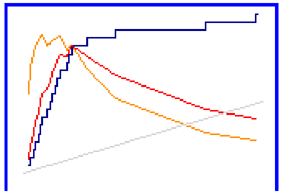	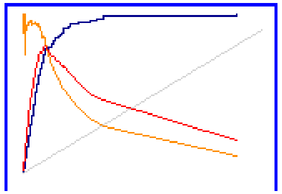	31. Zyklus<br>(59,20,79)<br>k_XERROR_5<br>Ginni_TRN: 92<br>Ginni_VAL: 91<br>Ginni__FT: 92	Intercept(-4.70,-7.40)<br>k_5_20(-6.93,-4.61)<br>k_5_31(14.96,6.28)<br>k_5_37(-1.27,-3.93)<br>k_2_19(-4.06,-3.36)<br>k_1_6(-1.39,-3.01)<br>k_1_31(-10.07,-4.61)	Intercept(-4.53,-8.33)<br>k_5_9(-2.28,-2.99)<br>k_5_20(-5.23,-4.42)<br>k_5_22(-2.43,-3.52)<br>k_5_31(14.38,7.03)<br>k_5_37(-0.83,-3.03)<br>k_1_6(-1.52,-3.66)<br>k_1_19(-4.00,-3.41)<br>k_1_31(-8.75,-4.70)	TRN:(100,78,00)<br>VAL:(88,80,65)<br>FT:(100,86,47)	TRN:(41,80,80)<br>VAL:(35,80,85)<br>FT:(5,76,94)

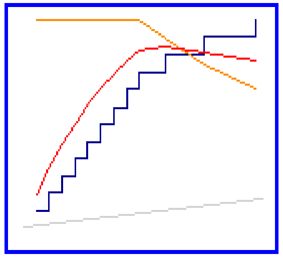	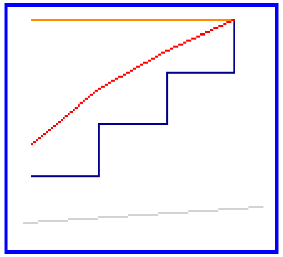	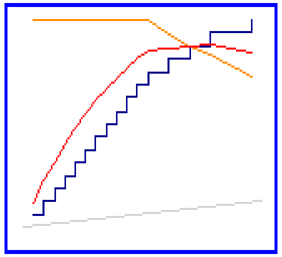	32. Zysten<br>(12,4,16)<br>k_XERROR_1<br>Ginni_TRN: 94<br>Ginni_VAL: 94<br>Ginni__FT: 95	Intercept(-7.60,-3.00)<br>k_1_32(8.37,2.49)	Intercept(-8.40,-3.26)<br>k_1_32(9.90,2.96)	TRN:(100,91,67)<br>VAL:(100,100,100)<br>FT:(100,83,73)	TRN:(75,83,100<br>VAL:(100,100,1<br>FT:(75,94,100)
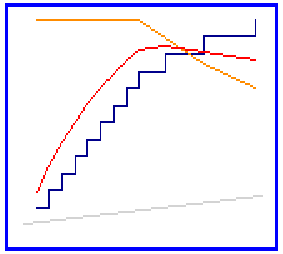	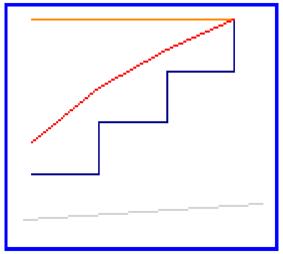	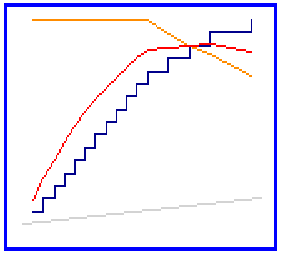	32. Zysten<br>(12,4,16)<br>k_XERROR_1<br>Ginni_TRN: 94<br>Ginni_VAL: 94<br>Ginni__FT: 95	Intercept(-7.60,-3.00)<br>k_1_32(8.37,2.49)	Intercept(-8.40,-3.26)<br>k_1_32(9.90,2.96)	TRN:(100,91,67)<br>VAL:(100,100,100)<br>FT:(100,83,73)	TRN:(75,83,100<br>VAL:(100,100,1<br>FT:(75,94,100)
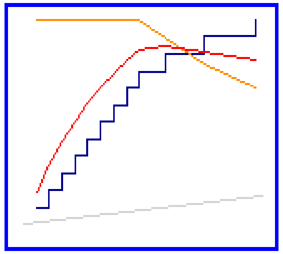	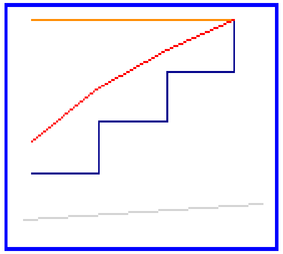	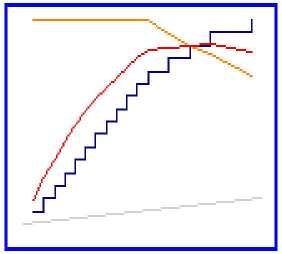	32. Zysten<br>(12,4,16)<br>k_XERROR_1<br>Ginni_TRN: 94<br>Ginni_VAL: 94<br>Ginni__FT: 95	Intercept(-7.60,-3.00)<br>k_1_32(8.37,2.49)	Intercept(-8.40,-3.26)<br>k_1_32(9.90,2.96)	TRN:(100,91,67)<br>VAL:(100,100,100)<br>FT:(100,83,73)	TRN:(75,83,100<br>VAL:(100,100,1<br>FT:(75,94,100)

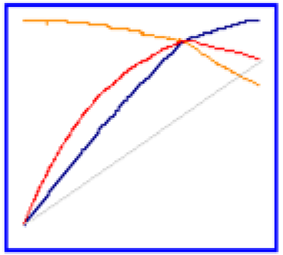	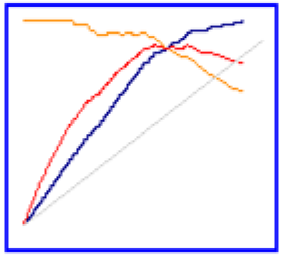	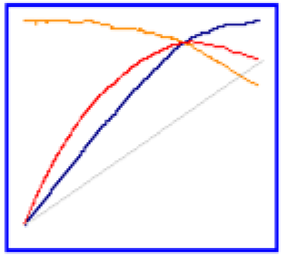	33. A, Allgemeine Fragen<br>(399,134,533)<br>k_XMISC_40<br>Ginni_TRN: 71<br>Ginni_VAL: 70<br>Ginni__FT: 71	Intercept(3.14,8.38)<br>k_40_33(23.39,8.25)<br>k_40_37(1.75,7.03)<br>k_30_33(-11.92,-4.51)<br>k_30_35(3.08,8.02)<br>k_20_36(0.93,3.86)<br>k_10_34(1.09,3.57)<br>k_5_33(-4.22,-4.86)<br>k_5_38(1.95,6.30)<br>k_2_5(1.46,4.64)	Intercept(3.11,9.65)<br>k_40_33(21.83,8.83)<br>k_40_37(1.79,7.54)<br>k_30_16(0.97,3.21)<br>k_30_32(-2.39,-2.56)<br>k_30_33(-9.27,-4.02)<br>k_30_35(3.05,9.11)<br>k_30_36(0.92,4.12)<br>k_20_38(1.54,6.30)<br>k_5_5(1.44,5.71)<br>k_5_12(0.79,2.74)<br>k_5_32(3.57,3.21)<br>k_5_33(-4.75,-5.88)<br>k_1_34(1.85,4.60)	TRN:(100,90,90)<br>VAL:(100,92,92)<br>FT:(100,88,88)	TRN:(27,90,90)<br>VAL:(32,84,84)<br>FT:(24,92,92)
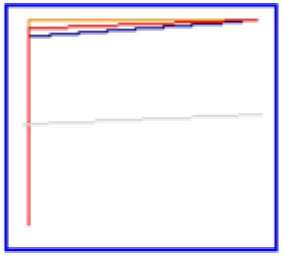	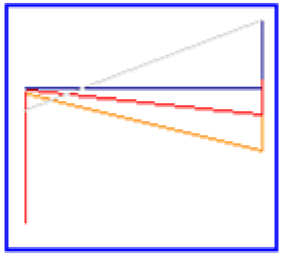	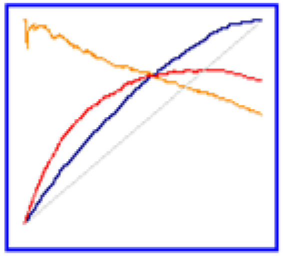	33. A, Allgemeine Fragen<br>(399,134,533)<br>svd_SBC_40<br>Ginni_TRN: 50<br>Ginni_VAL: 29<br>Ginni__FT: 63	Intercept(2.16,0.66)<br>s2(6011.27,1.20)<br>s3(10615.7,3.11)<br>s4(-8382.8,-0.43)<br>s5(18902.9,.)<br>s8(10797.5,.)<br>s10(26576.8,.)<br>s11(19521.5,.)<br>s12(11523.4,0.63)<br>s13(24870.5,1.59)<br>s15(-28355,-1.92)<br>s18(-27690,.)<br>s20(-12688,-0.89)<br>s22(-12115,.)<br>s34(18914.4,0.85)<br>s35(20354.2,0.69)<br>[illegible]	Intercept(0.01,0.10)<br>s3(5.40,3.54)<br>s5(2.75,3.55)<br>s6(-2.54,-2.63)<br>s9(3.28,3.48)<br>s10(3.59,3.51)<br>s13(3.34,3.13)<br>s15(-4.29,-3.97)<br>s18(-5.46,-5.19)<br>s23(2.49,2.39)<br>s24(-3.20,-3.17)<br>s38(-4.60,-4.02)<br>s64(-3.33,-2.96)<br>s70(3.09,2.67)<br>s96(5.16,4.31)<br>s155(4.06,3.20)<br>[illegible]	TRN:(100,100,100)<br>VAL:(64,54,64)<br>FT:(100,63,74)	TRN:(99,100,10<br>VAL:(67,100,67<br>FT:(1,95,73)
				s18(-27690,.)<br>s20(-12688,-0.89)<br>[illegible]	s38(-4.60,-4.02)<br>s64(-3.33,-2.96)<br>[illegible]		
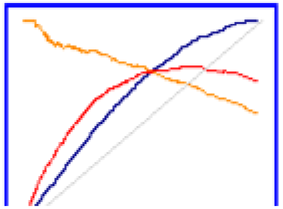	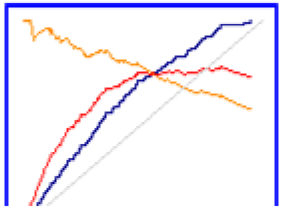	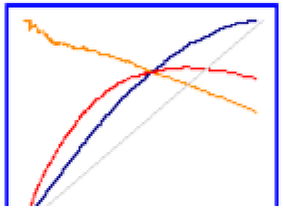	33. A, Allgemeine Fragen<br>(399,134,533)<br>k_SBC_1<br>Ginni_TRN: 64<br>Ginni_VAL: 63<br>Ginni__FT: 64	Intercept(2.45,11.28)<br>k_1_10(1.08,2.46)<br>k_1_12(0.82,3.72)<br>k_1_33(2.66,11.49)	Intercept(2.44,13.03)<br>k_1_10(1.04,2.74)<br>k_1_12(0.91,4.70)<br>k_1_20(-1.21,-3.33)<br>k_1_33(2.61,13.04)	TRN:(100,70,78)<br>VAL:(100,64,79)<br>FT:(100,69,80)	TRN:(9,87,72)<br>VAL:(6,98,70)<br>FT:(5,88,66)

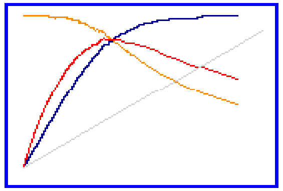	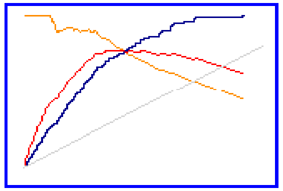	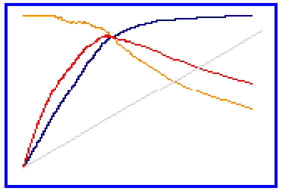	34. D,<br>Derzeitigebehandlung<br>(248,83,331)<br>pc_XMISC_40<br>Ginni_TRN: 00<br>Ginni_VAL: 78<br>Ginni__FT: 80	Intercept(-1.77,-9.66)<br>pC_2_5(3.46,2.29)<br>pC_8_6(-2.78,-2.74)<br>pC_11_7(7.96,2.73)<br>pC_10_6(4.75,3.00)<br>pC_15_3(-3.57,-2.59)<br>pC_16_1(-0.14,-2.63)<br>pC_18_2(1.16,3.21)<br>pC_19_1(-0.21,-3.18)<br>pC_24_5(9.66,4.20)<br>pC_25_2(0.97,2.69)<br>pC_26_1(-0.35,-5.52)<br>pC_26_7(-8.30,-3.01)<br>pC_29_1(-0.29,-3.84)<br>pC_34_1(2.18,11.87)<br>pC_34_2(-4.64,-9.08)<br>pC_34_3(6.78,6.45)<br>pC_34_7(10.24,3.23)<br>pC_35_1(-0.59,-6.43)<br>pC_36_6(5.48,2.67)<br>pC_37_1(-0.63,-6.87)<br>pC_37_7(14.68,2.48)	Intercept(-1.90,-11.58)<br>pC_8_1(0.11,2.29)<br>pC_8_6(-2.38,-2.65)<br>pC_9_3(3.24,4.24)<br>pC_10_1(0.20,3.05)<br>pC_10_4(2.74,2.80)<br>pC_13_6(2.50,1.84)<br>pC_14_3(4.30,3.38)<br>pC_19_1(-0.16,-2.44)<br>pC_22_7(6.81,2.96)<br>pC_23_3(-3.18,-4.13)<br>pC_24_5(8.79,4.12)<br>pC_25_6(-4.90,-2.85)<br>pC_26_1(-0.34,-5.50)<br>pC_26_5(5.22,3.90)<br>pC_26_7(-8.37,-3.49)<br>pC_27_7(-3.43,-2.37)<br>pC_28_5(9.12,2.74)<br>pC_29_1(-0.18,-2.78)<br>pC_31_1(0.29,4.11)<br>pC_34_1(2.27,13.40)<br>pC_34_2(-5.19,-10.99)<br>pC_34_3(6.92,7.43)<br>pC_34_7(12.07,4.21)<br>pC_35_1(-0.54,-6.44)<br>pC_36_6(4.86,2.74)<br>pC_37_1(-0.67,-8.65)	TRN:(100,89,81)<br>VAL:(100,85,71)<br>FT:(100,89,89)	TRN:(28,81,88)<br>VAL:(22,72,86)<br>FT:(19,85,85)
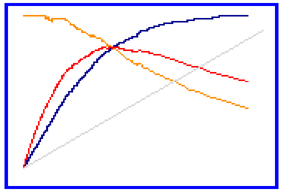	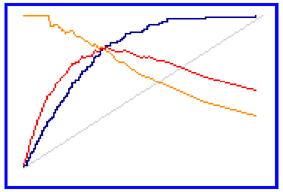	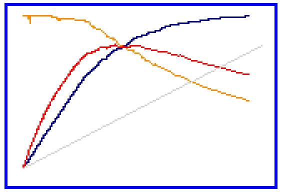	34. D,<br>Derzeitigebehandlung<br>(248,83,331)<br>k_XMISC_30<br>Ginni_TRN: 78<br>Ginni_VAL: 77<br>Ginni__FT: 79	Intercept(-3.55,-10.02)<br>k_30_28(0.47,3.39)<br>k_30_34(7.63,11.23)<br>k_30_37(-1.00,-6.71)<br>k_20_26(-0.79,-3.39)<br>k_20_27(-1.03,-2.29)<br>k_20_29(-1.12,-4.01)<br>k_5_35(-1.62,-6.20)<br>k_2_2(-0.91,-2.24)<br>k_2_34(-3.26,-4.63)	Intercept(-3.94,-11.60)<br>k_30_9(4.17,2.40)<br>k_30_13(-0.86,-3.04)<br>k_30_26(3.97,2.50)<br>k_30_28(0.56,3.20)<br>k_30_34(11.44,10.78)<br>k_30_37(-1.22,-7.29)<br>k_20_9(-3.80,-2.11)<br>k_20_26(-6.65,-3.44)<br>k_20_27(-1.45,-3.48)<br>k_20_29(-1.18,-4.40)<br>k_20_32(-0.90,-2.97)<br>k_20_36(-0.47,-2.00)<br>k_10_19(-1.18,-2.99)<br>k_10_34(-3.98,-3.30)<br>k_5_10(1.05,3.06)<br>k_5_26(2.40,2.58)<br>k_5_35(-1.48,-5.01)<br>k_2_17(-1.10,-2.70)<br>k_1_34(-2.19,-2.80)	TRN:(100,79,76)<br>VAL:(100,78,78)<br>FT:(100,84,77)	TRN:(25,80,82)<br>VAL:(31,81,81)<br>FT:(19,78,85)
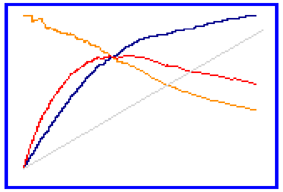	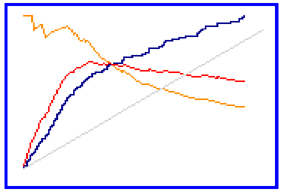	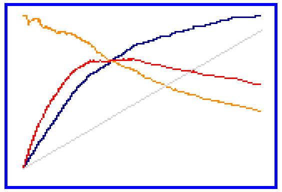	34. D,<br>Derzeitigebehandlung<br>(248,83,331)<br>k_SBC_5<br>Ginni_TRN: 75<br>Ginni_VAL: 72<br>Ginni__FT: 74	Intercept(-2.50,-9.47)<br>k_5_29(-0.99,-3.31)<br>k_5_32(3.06,2.57)<br>k_5_34(7.64,9.55)<br>k_2_28(0.62,4.64)<br>k_2_35(-1.19,-5.52)<br>k_1_32(-4.27,-3.17)<br>k_1_34(-4.21,-5.19)<br>k_1_37(-0.83,-5.08)	Intercept(-2.49,-11.08)<br>k_5_29(-0.95,-3.96)<br>k_5_34(6.99,10.64)<br>k_2_28(0.57,5.14)<br>k_1_32(-0.97,-3.09)<br>k_1_34(-3.35,-5.11)<br>k_1_35(-1.09,-5.57)<br>k_1_37(-0.77,-5.84)	TRN:(100,69,67)<br>VAL:(100,82,82)<br>FT:(100,65,65)	TRN:(9,79,82)<br>VAL:(10,60,60)<br>FT:(4,79,79)

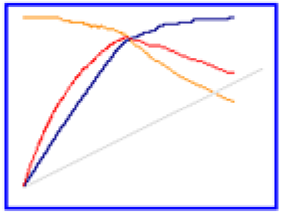	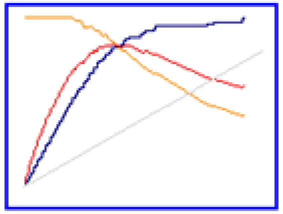	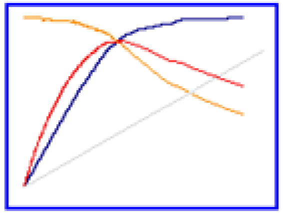	35. E. Ergebnisse<br>(232,78,310)<br>k_XMISC_40<br>Ginni_TRN: 82<br>Ginni_VAL: 80<br>Ginni__FT: 82	Intercept(-3.62,-7.71)<br>k_40_28(-5.23,-2.89)<br>k_40_35(12.67,10.19)<br>k_30_21(-10.35,-3.83)<br>k_30_29(-3.01,-3.85)<br>k_20_12(-2.46,-5.23)<br>k_20_21(9.22,3.35)<br>k_20_28(7.07,3.32)<br>k_20_30(-1.79,-3.56)<br>k_20_31(-0.82,-2.86)<br>k_10_2(-10.02,-3.94)<br>k_5_2(9.09,3.21)<br>k_5_18(-2.03,-2.43)<br>k_5_33(2.52,4.09)<br>k_2_3(-1.40,-2.60)<br>k_2_4(-11.27,-2.14)<br>k_2_28(-3.13,-3.07)<br>k_2_35(-5.97,-5.74)<br>k_2_38(1.78,3.95)<br>k_1_4(13.16,2.43)<br>k_1_29(3.02,2.74)	Intercept(-3.28,-9.29)<br>k_40_13(-0.86,-2.98)<br>k_40_21(-3.59,-4.08)<br>k_40_35(11.35,11.83)<br>k_20_6(-0.58,-2.54)<br>k_20_12(-1.84,-5.60)<br>k_10_2(-8.01,-3.80)<br>k_10_30(-4.10,-3.88)<br>k_5_2(7.74,3.36)<br>k_5_21(2.85,2.87)<br>k_5_33(1.74,3.90)<br>k_2_3(-1.15,-2.58)<br>k_2_28(-0.75,-4.43)<br>k_2_35(-6.13,-7.30)<br>k_1_4(1.03,3.38)<br>k_1_30(2.90,2.28)<br>k_1_38(1.24,3.48)	TRN:(100,90,90)<br>VAL:(100,86,78)<br>FT:(100,84,82)	TRN:(34,87,87)<br>VAL:(46,82,88)<br>FT:(10,89,90)
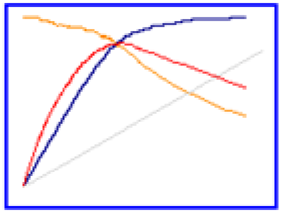	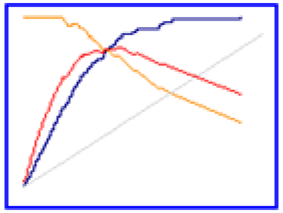	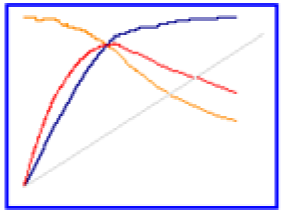	35. E. Ergebnisse<br>(232,78,310)<br>k_SBC_30<br>Ginni_TRN: 81<br>Ginni_VAL: 80<br>Ginni__FT: 81	Intercept(-2.84,-7.83)<br>k_30_12(-1.48,-4.27)<br>k_30_21(-8.41,-3.72)<br>k_30_35(9.29,9.88)<br>k_20_21(7.72,3.35)<br>k_20_30(-2.06,-4.83)<br>k_5_3(-1.37,-3.00)<br>k_5_16(-1.71,-2.88)<br>k_5_28(-0.66,-4.02)<br>k_5_33(2.27,4.54)<br>k_2_35(-3.62,-4.23)<br>k_2_38(1.42,3.84)<br>k_1_4(1.09,3.78)	Intercept(-2.72,-9.18)<br>k_30_21(-3.03,-3.53)<br>k_30_35(8.93,11.70)<br>k_20_12(-1.47,-6.16)<br>k_10_4(0.80,3.88)<br>k_10_28(-0.67,-5.12)<br>k_10_30(-3.72,-3.94)<br>k_5_21(2.79,2.87)<br>k_5_34(-1.35,-4.29)<br>k_2_3(-1.10,-2.77)<br>k_2_35(-4.52,-6.15)<br>k_1_30(2.78,2.43)	TRN:(100,83,81)<br>VAL:(100,78,78)<br>FT:(100,81,81)	TRN:(17,88,89)<br>VAL:(46,88,88)<br>FT:(10,89,89)
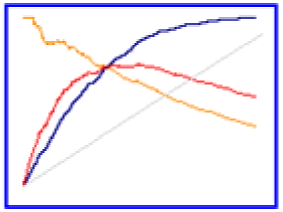	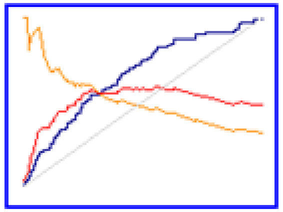	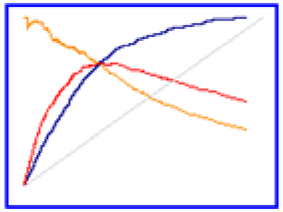	35. E. Ergebnisse<br>(232,78,310)<br>svd_SBC_40<br>Ginni_TRN: 76<br>Ginni_VAL: 66<br>Ginni__FT: 76	Intercept(-1.32,-10.32)<br>s2(4.94,4.96)<br>s4(10.95,7.05)<br>s5(-5.19,-4.73)<br>s10(-12.26,-7.55)<br>s15(7.34,4.66)<br>s18(12.94,7.96)<br>s21(-8.92,-5.95)<br>s27(-5.10,-3.30)<br>s51(-4.82,-2.91)<br>s54(-5.38,-3.45)<br>s60(4.67,2.83)<br>s64(4.61,2.91)<br>s70(-4.74,-2.82)<br>s80(5.47,3.24)<br>s122(-5.60,-3.08)<br>s185(-5.02,-2.69)<br>s200(-7.26,-3.99)<br>s233(-1.95,-2.65)<br>s240(1.92,2.65)<br>s382(-2.58,-2.96)<br>s384(3.73,3.93)<br>s401(-2.32,-2.58)	Intercept(-0.01,-0.02)<br>s1(-4.99,-3.08)<br>s2(4.05,4.67)<br>s4(7.34,5.75)<br>s5(-7.48,-7.06)<br>s8(3.40,2.76)<br>s9(-7.35,-5.60)<br>s10(-11.90,-8.20)<br>s13(-4.29,-3.27)<br>s15(8.66,6.10)<br>s18(9.48,7.26)<br>s21(-6.37,-5.13)<br>s48(4.37,3.23)<br>s51(-4.74,-3.33)<br>s64(4.49,3.31)<br>s73(-4.99,-3.51)<br>s80(5.17,3.46)<br>s94(-3.99,-2.75)<br>s122(-4.98,-3.31)<br>s143(-4.16,-2.88)<br>s212(4.99,3.13)<br>s217(-3.98,-2.60)<br>s298(-1.85,-2.60)	TRN:(100,62,62)<br>VAL:(100,46,60)<br>FT:(100,72,66)	TRN:(12,86,86)<br>VAL:(4,82,54)<br>FT:(4,74,81)

s384(3.73,3.93) s217(-3.98,-2.60)
s401(-2.32,-2.58) s298(-1.85,-2.60)

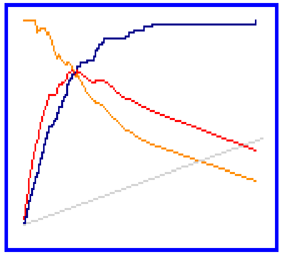	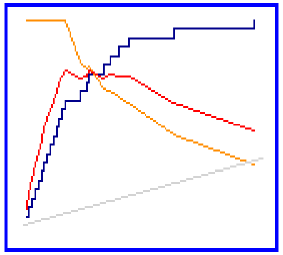	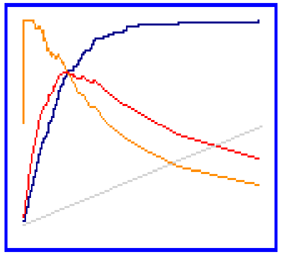	36. G, Gefuehle<br>(67,23,90)<br>k_SBC_2<br>Ginni_TRN: 93<br>Ginni_VAL: 93<br>Ginni__FT: 93	Intercept(-5.74,-9.80)<br>k_2_36(5.60,9.86)<br>k_1_11(1.05,2.61)<br>k_1_37(-1.80,-4.72)	Intercept(-5.50,-11.10)<br>k_2_36(6.09,11.08)<br>k_1_11(1.11,3.12)<br>k_1_37(-2.21,-5.94)	TRN:(100,78,58)<br>VAL:(100,100,60)<br>FT:(100,79,58)	TRN:(24,73,91)<br>VAL:(57,61,91)<br>FT:(23,71,91)
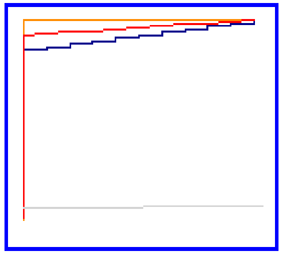	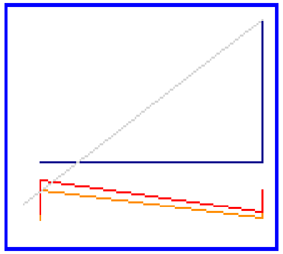	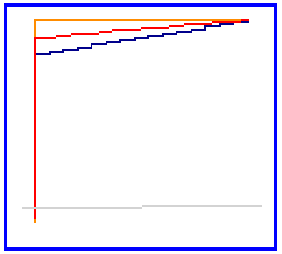	36. G, Gefuehle<br>(67,23,90)<br>svd_SBC_40<br>Ginni_TRN: 92<br>Ginni_VAL: 25<br>Ginni__FT: 92	Intercept(-36232,-31.55)<br>s2(-67452,-22.73)<br>s5(98167.4,6.07)<br>s6(77455.9,15.93)<br>s13(-58221,-2.89)<br>s15(76347.3,8.80)<br>s16(77224.6,7.73)<br>s31(74393.5,5.56)<br>s34(30230.4,3.10)<br>s36(-44618,.)<br>s44(-17115,-2.07)<br>s61(-45462,-7.43)<br>s63(-101627,.)<br>s79(-60764,-12.70)<br>s88(-74700,.)<br>s93(53627.4,4.56)<br>[illegible]	Intercept(-56754,-28.44)<br>s2(-116316,-20.10)<br>s3(106992,6.82)<br>s4(-70473,.)<br>s5(127819,36.93)<br>s13(-150799,-6.31)<br>s16(103850,.)<br>s19(57125.5,.)<br>s27(51642.3,5.76)<br>s31(109156,8.72)<br>s39(105519,.)<br>s42(90367.9,8.52)<br>s47(46447.9,.)<br>s61(-86571,.)<br>s63(-143344,-6.01)<br>s69(119128,14.50)<br>[illegible]	TRN:(100,100,100)<br>VAL:(17,17,17)<br>FT:(100,100,100)	TRN:(100,100,1[illegible]<br>VAL:(30,30,30)<br>FT:(100,100,10[illegible]
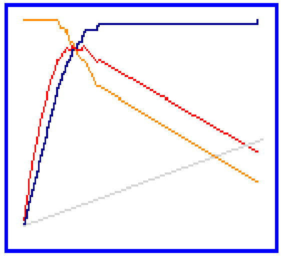	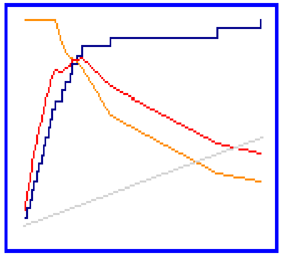	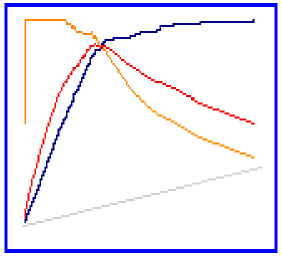	36. G, Gefuehle<br>(67,23,90)<br>k_AIC_30<br>Ginni_TRN: 85<br>Ginni_VAL: 92<br>Ginni__FT: 94	Intercept(-11.54,-6.79)<br>k_30_36(12.76,5.81)<br>k_20_14(-0.63,-1.94)<br>k_5_37(-2.32,-3.69)<br>k_2_17(-4.06,-3.21)<br>k_1_8(-5.35,-2.14)<br>k_1_27(-4.67,-2.60)<br>k_1_36(-6.20,-3.56)<br>k_1_38(-1.57,-2.17)	Intercept(-8.38,-9.09)<br>k_30_7(-4.08,-3.14)<br>k_30_36(15.11,4.17)<br>k_20_17(-2.30,-3.20)<br>k_20_36(-7.80,-2.24)<br>k_20_38(-1.70,-3.81)<br>k_5_27(-3.30,-2.96)<br>k_1_7(4.32,2.67)<br>k_1_11(1.18,2.71)<br>k_1_37(-2.18,-3.81)	TRN:(100,89,67)<br>VAL:(100,77,77)<br>FT:(100,92,62)	TRN:(67,85,99)<br>VAL:(57,87,87)<br>FT:(34,86,94)

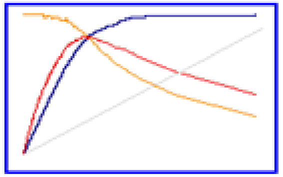	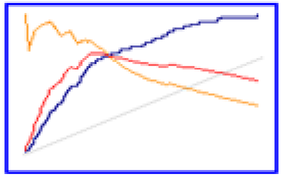	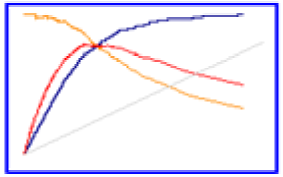	37. I, interpretation<br>(181,61,242)<br>k_AIC_40<br>Ginni_TRN: 85<br>Ginni_VAL: 80<br>Ginni__FT: 84	Intercept(-4.91,-8.33)<br>k_40_1(-3.34,-3.92)<br>k_40_12(6.52,3.55)<br>k_40_37(20.04,7.39)<br>k_30_13(-1.06,-2.38)<br>k_30_19(-2.52,-2.59)<br>k_30_28(-0.62,-2.68)<br>k_30_34(-2.10,-5.80)<br>k_20_6(2.35,2.66)<br>k_20_12(-5.40,-2.32)<br>k_20_37(-15.77,-4.80)<br>k_20_38(-2.76,-6.31)<br>k_10_30(-5.80,-2.64)<br>k_10_37(11.68,4.31)<br>k_5_5(-1.21,-4.35)<br>k_5_12(-3.76,-2.55)<br>k_5_14(-0.61,-1.97)<br>k_5_25(-0.99,-2.44)<br>k_5_30(5.82,2.49)<br>k_5_37(-9.67,-5.39)<br>k_2_6(-4.52,-4.13)<br>k_2_19(8.89,3.36)<br>k_2_31(5.64,2.29)<br>k_1_1(3.22,3.15)<br>k_1_19(-8.44,-3.31)<br>k_1_31(-7.62,-2.95)	Intercept(-4.65,-11.24)<br>k_40_1(-0.79,-3.93)<br>k_40_37(14.56,8.12)<br>k_30_4(-0.78,-2.91)<br>k_30_34(-1.94,-7.58)<br>k_20_6(2.11,3.11)<br>k_20_28(-0.87,-5.49)<br>k_20_37(-9.42,-4.37)<br>k_20_38(-2.00,-6.81)<br>k_10_37(6.39,3.45)<br>k_5_5(-0.56,-2.33)<br>k_5_37(-6.69,-5.22)<br>k_2_6(-7.77,-3.62)<br>k_2_12(-1.28,-3.90)<br>k_2_31(5.18,2.93)<br>k_1_6(4.06,2.16)<br>k_1_19(-2.51,-3.67)<br>k_1_31(-6.52,-3.50)	TRN:(100,83,82)<br>VAL:(100,78,75)<br>FT:(100,88,67)	TRN:(31,86,87)<br>VAL:(3,69,70)<br>FT:(13,72,89)
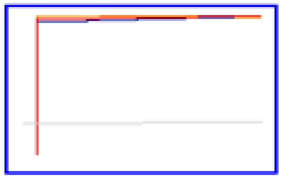	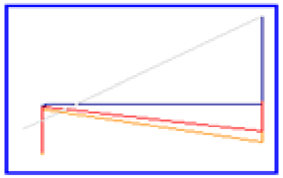	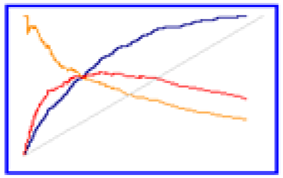	37. I, interpretation<br>(181,61,242)<br>svd_SBC_40<br>Ginni_TRN: 77<br>Ginni_VAL: 28<br>Ginni__FT: 74	Intercept(-41080,-21.73)<br>s3(-155102,.) s4(-90462,.)<br>s8(-122292,.) s9(-81881,.)<br>s10(279114,.)<br>s11(-41124,.)<br>s12(142011,.)<br>s15(228358,.)<br>s17(-128679,.)<br>s19(104061,.)<br>s20(129830,.)<br>s21(132053,.)<br>s26(59498.3,.)	Intercept(-1.22,-12.86)<br>s3(-5.08,-3.56)<br>s8(-2.97,-3.14)<br>s10(7.25,6.54)<br>s11(-3.02,-2.86)<br>s15(5.62,4.72)<br>s20(3.82,3.27)<br>s27(-3.45,-2.84)<br>s31(-3.50,-2.86)<br>s36(-4.31,-3.30)<br>s47(-3.84,-2.96)<br>s59(-4.57,-3.37)	TRN:(100,99,99)<br>VAL:(34,25,34)<br>FT:(100,52,48)	TRN:(99,100,10[illegible]<br>VAL:(36,100,38[illegible]<br>FT:(5,70,78)
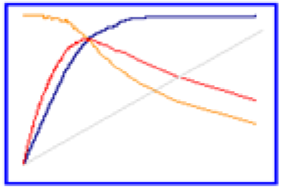	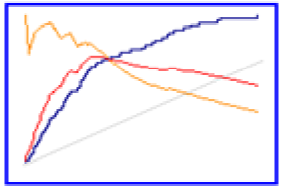	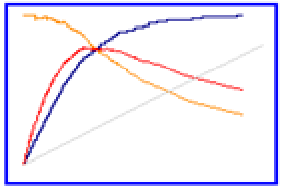	37. I, interpretation<br>(181,61,242)<br>k_AIC_40<br>Ginni_TRN: 85<br>Ginni_VAL: 80<br>Ginni__FT: 84	Intercept(-4.91,-8.33)<br>k_40_1(-3.34,-3.92)<br>k_40_12(6.52,3.55)<br>k_40_37(20.04,7.39)<br>k_30_13(-1.06,-2.38)<br>k_30_19(-2.52,-2.59)<br>k_30_28(-0.62,-2.68)<br>k_30_34(-2.10,-5.80)<br>k_20_6(2.35,2.66)<br>k_20_12(-5.40,-2.32)<br>k_20_37(-15.77,-4.80)<br>k_20_38(-2.76,-6.31)<br>k_10_30(-5.80,-2.64)<br>k_10_37(11.68,4.31)<br>k_5_5(-1.21,-4.35)<br>k_5_12(-3.76,-2.55)<br>k_5_14(-0.61,-1.97)<br>k_5_25(-0.99,-2.44)<br>k_5_30(5.82,2.49)<br>k_5_37(-9.67,-5.39)<br>k_2_6(-4.52,-4.13)<br>k_2_19(8.89,3.36)<br>k_2_31(5.64,2.29)<br>k_1_1(3.22,3.15)<br>k_1_19(-8.44,-3.31)<br>k_1_31(-7.62,-2.95)	Intercept(-4.65,-11.24)<br>k_40_1(-0.79,-3.93)<br>k_40_37(14.56,8.12)<br>k_30_4(-0.78,-2.91)<br>k_30_34(-1.94,-7.58)<br>k_20_6(2.11,3.11)<br>k_20_28(-0.87,-5.49)<br>k_20_37(-9.42,-4.37)<br>k_20_38(-2.00,-6.81)<br>k_10_37(6.39,3.45)<br>k_5_5(-0.56,-2.33)<br>k_5_37(-6.69,-5.22)<br>k_2_6(-7.77,-3.62)<br>k_2_12(-1.28,-3.90)<br>k_2_31(5.18,2.93)<br>k_1_6(4.06,2.16)<br>k_1_19(-2.51,-3.67)<br>k_1_31(-6.52,-3.50)	TRN:(100,83,82)<br>VAL:(100,78,75)<br>FT:(100,88,67)	TRN:(31,86,87)<br>VAL:(3,69,70)<br>FT:(13,72,89)

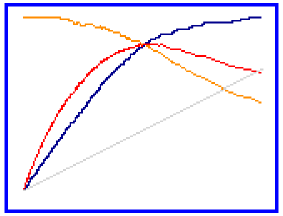	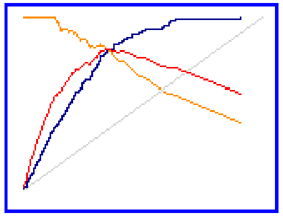	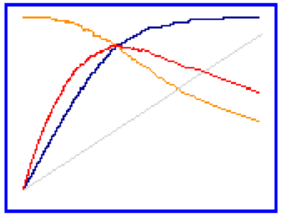	38. M, Moeglichkeiten<br>(263,88,351)<br>k_XMISC_40<br>Ginni_TRN: 79<br>Ginni_VAL: 77<br>Ginni__FT: 79	Intercept(-2.86,-7.72)<br>k_40_4(-0.80,-3.26)<br>k_40_17(-1.38,-4.01)<br>k_40_19(2.60,4.40)<br>k_40_21(-0.60,-2.63)<br>k_40_27(1.93,2.62)<br>k_40_38(10.39,5.67)<br>k_30_6(0.66,2.71)<br>k_30_15(-0.73,-3.13)<br>k_30_28(-2.20,-3.51)<br>k_30_37(-1.88,-6.26)<br>k_20_26(3.02,3.78)<br>k_20_38(7.20,3.13)<br>k_10_12(-0.45,-1.56)<br>k_10_36(-1.53,-4.49)<br>k_10_38(-6.02,-2.82)<br>k_5_38(-6.18,-4.04)<br>k_2_28(2.07,2.96)<br>k_1_19(-3.01,-2.95)<br>k_1_26(-3.18,-3.02)<br>k_1_27(-4.18,-3.13)	Intercept(-2.94,-8.92)<br>k_40_5(-0.44,-2.67)<br>k_40_19(3.32,6.18)<br>k_40_21(-4.10,-3.27)<br>k_40_28(-0.78,-6.14)<br>k_40_35(1.15,5.28)<br>k_40_38(14.38,11.73)<br>k_30_6(0.57,2.86)<br>k_30_12(-0.95,-3.86)<br>k_30_36(-1.35,-5.65)<br>k_20_17(-2.12,-5.53)<br>k_20_21(3.58,2.74)<br>k_20_26(2.33,3.50)<br>k_20_37(-1.92,-7.78)<br>k_10_38(-4.05,-2.52)<br>k_5_9(-0.65,-2.68)<br>k_5_38(-5.03,-3.90)<br>k_1_15(-0.94,-4.11)<br>k_1_19(-3.33,-3.43)<br>k_1_26(-2.68,-2.92)	TRN:(100,84,82)<br>VAL:(100,82,78)<br>FT:(100,84,84)	TRN:(30,85,87)<br>VAL:(30,81,84)<br>FT:(18,83,83)
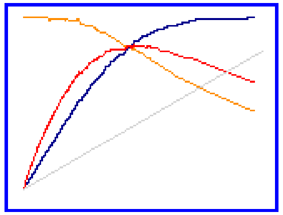	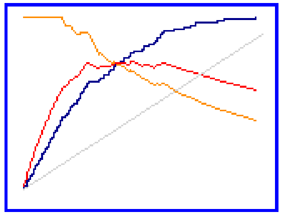	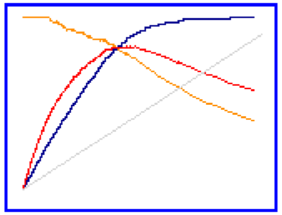	38. M, Moeglichkeiten<br>(263,88,351)<br>pc_SBC_40<br>Ginni_TRN: 79<br>Ginni_VAL: 75<br>Ginni__FT: 78	Intercept(-1.43,-8.93)<br>pC_8_3(1.16,2.59)<br>pC_11_2(1.88,3.74)<br>pC_14_2(1.82,3.16)<br>pC_14_6(10.64,3.58)<br>pC_15_1(-0.26,-3.48)<br>pC_17_1(-0.28,-4.38)<br>pC_19_1(0.31,4.28)<br>pC_19_2(-1.03,-4.00)<br>pC_25_5(-4.05,-2.75)<br>pC_27_2(-1.24,-4.36)<br>pC_27_4(3.01,4.20)<br>pC_28_1(-0.17,-2.81)<br>pC_28_2(2.29,3.07)<br>pC_30_6(-4.14,-2.49)<br>pC_35_5(5.89,2.71)<br>pC_36_1(-0.46,-5.72)<br>pC_37_1(-0.92,-7.01)<br>pC_38_1(2.70,11.68)<br>pC_38_2(-7.53,-9.84)<br>pC_38_3(10.34,6.70)	Intercept(-1.19,-9.90)<br>pC_15_1(-0.31,-5.19)<br>pC_17_1(-0.26,-4.75)<br>pC_19_1(0.40,6.15)<br>pC_19_2(-1.04,-4.81)<br>pC_22_2(-1.02,-3.25)<br>pC_24_5(-7.54,-3.79)<br>pC_27_2(-0.65,-3.03)<br>pC_27_4(2.65,4.60)<br>pC_28_1(-0.18,-3.47)<br>pC_32_4(-2.45,-2.70)<br>pC_33_7(-8.49,-2.81)<br>pC_36_1(-0.34,-5.34)<br>pC_37_1(-0.77,-7.96)<br>pC_38_1(2.24,13.84)<br>pC_38_2(-6.26,-11.26)<br>pC_38_3(10.00,8.17)	TRN:(100,82,79)<br>VAL:(100,69,62)<br>FT:(100,79,76)	TRN:(23,86,89)<br>VAL:(41,81,92)<br>FT:(18,88,91)
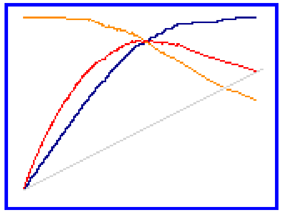	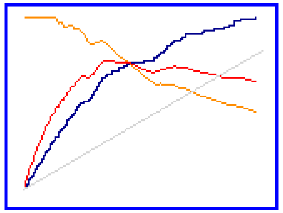	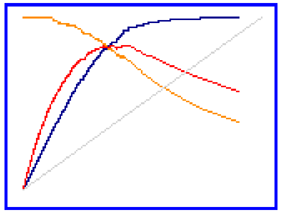	38. M, Moeglichkeiten<br>(263,88,351)<br>pc_XMISC_40<br>Ginni_TRN: 80<br>Ginni_VAL: 74<br>Ginni__FT: 79	Intercept(-1.70,-8.75)<br>pC_6_1(0.24,2.48)<br>pC_8_3(2.12,3.91)<br>pC_8_4(1.98,2.56)<br>pC_8_6(-3.24,-2.72)<br>pC_10_2(1.47,3.45)<br>pC_11_2(1.78,3.06)<br>pC_14_2(1.83,2.80)<br>pC_14_6(15.74,4.65)<br>pC_14_7(-17.76,-3.20)<br>pC_15_1(-0.44,-4.55)<br>pC_17_1(-0.38,-4.69)<br>pC_19_1(0.52,5.86)<br>pC_19_2(-1.39,-4.37)<br>pC_22_2(-1.71,-3.65)<br>pC_24_5(-8.14,-2.86)	Intercept(-1.24,-9.72)<br>pC_5_2(1.51,2.91)<br>pC_5_6(7.17,2.67)<br>pC_6_1(0.20,2.96)<br>pC_8_4(1.16,2.04)<br>pC_14_7(-11.45,-2.72)<br>pC_15_1(-0.34,-5.42)<br>pC_17_1(-0.32,-4.95)<br>pC_19_1(0.36,5.26)<br>pC_19_2(-1.06,-4.48)<br>pC_22_2(-1.12,-3.22)<br>pC_24_5(-7.20,-3.39)<br>pC_26_1(0.18,2.79)<br>pC_26_2(-0.98,-3.07)<br>pC_27_2(-0.64,-2.75)<br>pC_27_4(2.29,3.66)	TRN:(100,88,84)<br>VAL:(100,84,84)<br>FT:(100,77,75)	TRN:(35,85,89)<br>VAL:(30,67,67)<br>FT:(18,92,94)
